# Supplementary material for: Unveiling the mechanism of triphos-Ru catalysed C–O bond disconnections in polymers
Source: Nat Commun. 2024 Jul 5;15:5656. doi: 10.1038/s41467-024-50083-9 (PMC11226426; doi:10.1038/s41467-024-50083-9)
Supplement: Supplementary file 1 — Supplementary Informations [file 41467_2024_50083_MOESM1_ESM.pdf]

Supplementary Information

Unveiling the mechanism of triphos-Ru catalysed C–O bond disconnections in polymers

Alexander Ahrens[1]*, Gabriel Martins Ferreira Batista[1], Hans Christian D. Hammershøj[1], Emil Vincent Schwibinger[1], Ainara Nova[2]*, Troels Skrydstrup[1]*

[1]Department of Chemistry and Interdisciplinary Nanoscience Center (iNANO), Aarhus University, Gustav Wieds Vej 14, 8000 Aarhus C, Denmark.

[2]Department of Chemistry, Hylleraas Centre for Quantum Molecular Sciences and Centre for Materials Science and Nanotechnology, University of Oslo, N-0315 Oslo, Norway.

*Corresponding author. Email: aahrens@inano.au.dk, a.n.flores@kjemi.uio.no, ts@chem.au.dk

Contents

1. General Information 2

2. Experimental Section 4

2.1 Synthesis of compounds 4

triphos-Ru-TMM 4

triphos[xyl] ((2-((bis(3,5-dimethylphenyl)phosphaneyl)methyl)-2-methylpropane-1,3-diyl)bis(bis(3,5-dimethylphenyl)phosphane)) 4

triphos[xyl]-Ru-Cl_2 5

Me-BPA (4-(2-(4-methoxyphenyl)propan-2-yl)phenol) 5

Model 1 (1,3-bis(4-(2-(4-methoxyphenyl)propan-2-yl)phenoxy)propan-2-ol) 5

Ketone III (1-(4-(2-(4-methoxyphenyl)propan-2-yl)phenoxy)propan-2-one) 6

Lignin model (2-phenoxy-1-phenylethan-1-ol) 6

Model 2 (1-(4-(2-(4-methoxyphenyl)propan-2-yl)phenoxy)-3-(phenethylamino)propan-2-ol) 6

2.2 Catalytic deconstructions 7

General Procedure 7

Deconstruction of Model 2 8

2.3 Reactivity of the TMM-precatalyst 9

2.4 Role of ruthenium phenolates 13

Observation of triphos-Ru-(Me-BPA)$_2$ 13

Synthesis of Ru-1 (triphos-Ru-(2-fluorophenolate)$_2$) 13

Synthesis of Ru-2 (triphos-Ru-(2,6-difluorophenolate)$_2$) 15

Reacting Ru-1 with isopropanol 16

Reaction of Ru-2 with potassium 1-phenylethanolate 16

Synthesis of I7[xyl] 18

3. NMR Spectra 21

Diaurated 2-methylleneallyl ruthenium complex 21

Ru-1 (triphos-Ru-(2-fluorophenolate)$_2$) 23

Ru-2 (triphos-Ru-(2,6-difluorophenolate)$_2$) 25

Model 2 (1-(4-(2-(4-methoxyphenyl)propan-2-yl)phenoxy)-3-(phenethylamino)propan-2-ol) 27

Amine 1 (1-(isopropyl(phenethyl)amino)propan-2-ol) (mixed with amine 2) 28

Amine 2 (1-(isopropyl(phenethyl)amino)propan-2-ol) (mixed with amine 1) 29
triphos^{xyl}-Ru-Cl_2 30
4. Theoretical section 32
4.1 Computational details 32
4.2 Mechanism of the catalyst activation (energy profiles and discussion) 34
4.3 Formulation of catalytic cycle (energy profiles and discussion) 36
4.4 Reported data for individual species and cycles 42
4.5 Intrinsic reaction coordinate (IRC) calculations for computed transition states 48
5. X-Ray crystallographic section 59
6. References 64

1. General Information

Unless otherwise stated, all reactions were set-up and worked up in a glovebox under an atmosphere of argon. All chemicals were purchased from Sigma-Aldrich, Tokyo Chemical Industry (TCI) or Strem Chemicals and used as received. Tetrakis(dimethylsulfoxide)dichlororuthenium(II) was donated by Heraeus Precious Metals and used as received. THF, toluene, CH_2Cl_2 and MeCN were retrieved from a MBraun SP-800 purification system, degassed using argon and stored over 3 Å molecular sieves. The remaining solvents were purchased from Sigma-Aldrich degassed using argon, stored over 3 Å molecular sieves and used without further purification.

Thin layer chromatography (TLC) was carried out on pre-coated aluminium sheets ALUGRAM® Xtra SIL G/UV254 purchased by Macherey-Nagel. Visualisation of the products was achieved by UV-light irradiation (366 nm) and / or staining with a potassium permanganate in water.

Automated flash column chromatography (AFCC) was carried out with Interchim PuriFlash XS520Plus with 30 µm prepacked columns. Celite®545, coarse, was used for filtration.

Gas chromatography - mass spectrometry (GC-MS) were measured with an Agilent 8890 gas chromatograph coupled with an Agilent 5977B mass selective detector.

High resolution mass spectrometry (HRMS): ESI(+) spectral analysis were measured with a Bruker Maxis Impact Spectrometer. MALDI spectral analysis were measured on a Bruker Autoflex maX MALDI-TOF MS spectrometer using a MTP 384 target plate polished steel BC.

Nuclear Magnetic Resonance spectroscopy: ^{1}H NMR, ^{13}C NMR, ^{19}F NMR and ^{31}P NMR spectra were recorded on a Bruker 400 MHz Ascend spectrometer. Chemical shifts were given as δ value (ppm) with reference to residual solvent signal of the deuterated solvent. The peak patterns are indicated as follows: s, singlet; d, doublet; t, triplet; m, multiplet; q, quartet. Multiplicities reported for ^{13}C NMR spectra were assigned using APT, DEPT-90 and DEPT-135 spectra. Chemical structures were identified with the help of ^{1}H, ^{1}H COSY; ^{1}H,^{13}C HSQC; ^{1}H,^{13}C HMBC and ^{1}H,^{31}P HMBC NMR experiments. The coupling constants, *J*, are reported in Hertz (Hz). The spectra were calibrated to the residual solvent signals[1]. NMR spectra were processed with MestReNova Version 14.2.1-27684.

Reaction set up: Operando monitoring experiments using NMR spectroscopy were set up in an Argon charged glovebox using Low Pressure Vacuum NMR tubes (5 mm O.D., 177,8 mm) purchased from SP Wilmad-LabGlass. Reactions were heated in a silicone oil bath. Preparative / deconstruction reactions were set up in an Argon charged glovebox using a 10 ml COtube (pressure tube approved up to 5 bar) sealed with PTFE/silicon seals purchased from SyTracks as reaction vessel, a Teflon-coated stirring bar. Reactions were stirred in metal heating blocks at 650 rpm.

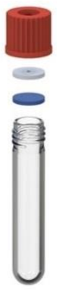

Supplementary Figure 1 | 10 ml COtube with screw cap, Teflon disc and septum.

2. Experimental Section

2.1 Synthesis of compounds

triphos-Ru-TMM

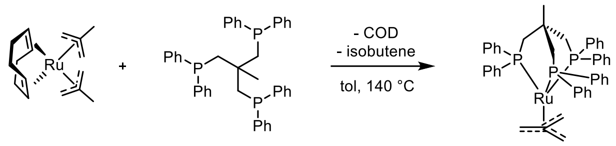

In an Argon-charged glovebox in a 10 ml COtube, 128 mg (0.40 mmol, 1.0 equiv) of (COD)Ru(2-methylallyl)$_2$ and 250 mg (0.40 mmol, 1.0 equiv) of 1,1,1 tris(diphenylphosphinomethyl)ethane were suspended in 3 ml of toluene. The reaction mixture was stirred at 140 °C over-night in a metal block, forming a grey to yellow suspension. After letting the reaction cool to room temperature, 3 ml of pentane were added and the reaction mixture placed at in a freezer - 30 °C. Afterwards, the solution was centrifuged, decanted off and the precipitate washed 2 ml of pentane / toluene 1/1. The process was repeated until the product was obtained as an off white to colourless solid, in a yield of 262 mg (0.34 mmol, 84%).

^{1}H NMR (CD$_2$Cl$_2$, 400 MHz, 25 °C): δ = 7.12 – 7.01 (m, 18H), 6.95 - 6.91 (m, 12H), 2.24 - 2.22 (m, 6H), 1.62 (s, 6H), 1.39 (s, 3H) ppm; ^{31}P NMR (CD$_2$Cl$_2$, 162 MHz, 25 °C): δ = 34.5 ppm. The NMR spectra are in agreement with reported data[1].

triphos^{xyl} ((2-((bis(3,5-dimethylphenyl)phosphaneyl)methyl)-2-methylpropane-1,3-diyl)bis(bis(3,5-dimethylphenyl)phosphane))

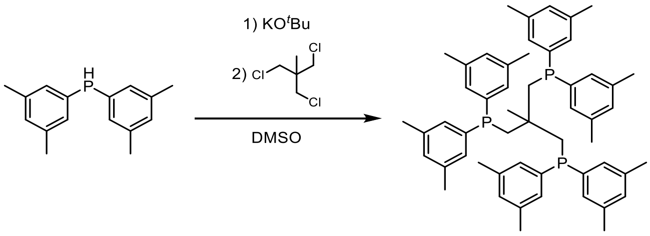

In an argon charged glovebox, 500 mg (2.06 mmol, 4.0 equiv) of bis(3,5-dimethylphenyl)phosphane was dissolved in 8 ml of DMSO in a 40 ml COtube. At rt, 231 mg (2.06 mmol, 4.0 equiv) of potassium *tert*-butoxide was added in portions, upon which the reaction mixture turns deep red immediately. After stirring for 30 min at rt, 71.1 µl (90.4 mg, 0.52 mmol, 1.0 equiv) of 1,3-dichloro-2-(chloromethyl)-2-methylpropane dissolved in 2 ml of DMSO were added, and the reaction mixture was stirred for 3 d at rt. Subsequently, the reaction mixture was carefully quenched with 10 ml of degassed water, and the crude product extracted with 3 times 4 ml of ethyl ether. The organic phase was passed through a pipette packed with MgSO$_4$ and the solvent removed *in vacuo*. The residue was taken up in 1 ml of toluene, overlayered with 3 ml of pentane and placed in a freezer at – 30 °C. The 254 mg of the product were obtained as colourless crystalline solid. A second crop of crystalls (135 mg) was obtained from the mother liquor in the same manner, resulting in an overall yield of 94% (389 mg, 491 µmol).

^{1}H NMR (toluene-d_8, 400 MHz, 25 °C): δ = 7.27 (s, 12H), 6.67 (s, 6H), 2.84 (d, J = 2.9 Hz, 6H), 2.08 (s, 36H), 1.29 (s, 3H); ^{31}P NMR (toluene-d_8, 162 MHz, 25 °C): δ = - 26.0 ppm. The NMR spectra are in agreement with reported data[2].

triphos^{xyl}-Ru-Cl_2

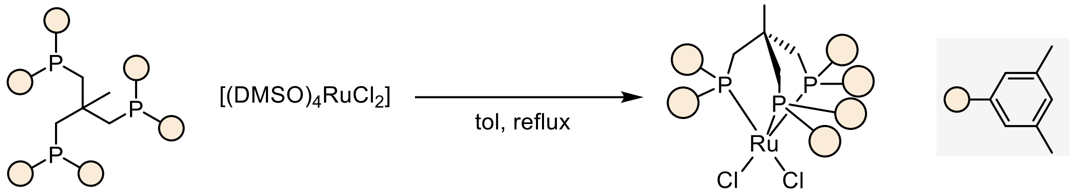

In an argon-charged glovebox in 10 ml pressure vial, 290 mg (0.37 mmol, 1 equiv) of triphos^{xyl}, 177 mg (0.37 mmol, 1 equiv) of $[(DMSO)_4RuCl_2]$ and 2 ml of toluene were mixed. The reaction vessel was sealed and stirred at 140 °C over-night, resulting in bright yellow precipitate. 1 ml of pentane was added, the pressure vial centrifuged and decanted. The complex was washed two times with a 2:1 tol:pentane mixture in the same manner, and then dissolved in DCM and recrystallised with pentane, affording triphos^{xyl}-Ru-Cl_2 as bright yellow solid (turning red during drying) in a yield of 44% (304 mg, 0.16 mmol, first batch). Unlike the airstable $[triphos\text{-}Ru\text{-}Cl_2]_2$[8], triphos^{xyl}-Ru-$Cl_2$ was found to slowly decompose under air, possibly due to the lack of dimerisation.

^{1}H NMR (CD_2Cl_{2k}, 400 MHz, 25 °C): δ = 7.04 – 7.02 (m, 12H), 6.76 (s, 6H), 2.30 – 2.28 (m, 6H), 2.05 (s, 36H), 1.62 (d, J = 3.1 Hz, 3H) ppm; ^{13}C NMR (CD_2Cl_2, 162 MHz, 25 °C): δ = 137.3 (s, 12C), 135.9 (s, 6C), 132.0 (d, 6C), 130.5 (d, 12C), 40.1 (s, 1C), 37.8 (q, 1C), 35.0 (t, 3C), 21.4 (q, 12H) ppm; ^{31}P NMR (CD_2Cl_2, 162 MHz, 25 °C): δ = 50.1 (s, 3P) ppm

Me-BPA (4-(2-(4-methoxyphenyl)propan-2-yl)phenol)

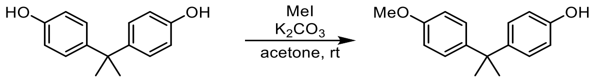

11.4 g (50.0 mmol, 1.0 equiv) of bisphenol A were dissolved in 100 ml of acetone in a 250 ml round bottom flask under air. Under stirring, 10.4 g (75.0 mmol, 1.5 equiv) of potassium carbonate were added, forming a suspension. Then, 3.11 ml (7.10 g, 50.0 mmol, 1 equiv) of methyl iodide were added. The reaction mixture was stirred over-night at room temperature. Afterwards, the suspension was filtered over a plug of silica. The solvent was removed *in vacuo*. Column chromatography over silica gel using a gradient of 15/1 pentane/ethyl acetate to 10/1 pentane/ethyl acetate afforded Me-BPA as colourless highly viscous oil in a yield of 53% (6.40 g, 52.8 mmol).

^{1}H NMR ($CDCl_3$, 400 MHz, 25 °C): δ = 7.16 -7.14 (m, 2H), 7.11 - 7.09 (m, 2H), 6.83 - 6.81 (m, 2H), 6.74- 6.72 (m, 2H), 4.76 (s, 1H), 3.80 (s, 3H), 1.64 (s, 6H) ppm. The NMR spectra are in agreement with reported data[1].

Model 1 (1,3-bis(4-(2-(4-methoxyphenyl)propan-2-yl)phenoxy)propan-2-ol)

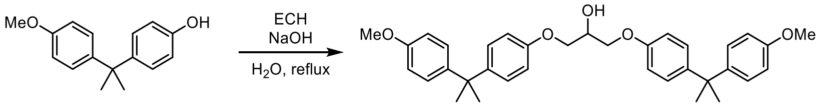

model 1

1.43 g (5.90 mmol, 2.0 equiv) of Me-BPA were suspended in 50 ml of water in a 100 ml round bottom flask under air. 236 mg (5.90 mmol, 2 equiv) of sodium hydroxide were added and the mixture was stirred at room temperature for 10 min. Then, 231 µl (273 mg, 2.95 mmol, 1 equiv) of epichlorohydrin (ECH) were added. The flask was equipped with a reflux condenser and the mixture was heated to reflux over-night under vigorous stirring. The reaction mixture was allowed to cool to room temperature and 30 ml of DCM were added. The crude product was extracted using three times 30 ml of DCM. The combined organic phases were dried over $MgSO_4$, filtered, and the solvent removed *in vacuo*. Column chromatography over silica gel using a gradient of 10/1 pentane/ethyl acetate to 5/1 pentane/ethyl acetate, afforded model 1 as colourless highly viscous oil in a yield of 66% (1.05 g, 1.94 mmol). Some but not all batches of model 1 crystallised slowly over the course of days to weeks.

^{1}H NMR ($CDCl_3$, 400 MHz, 25 °C): δ = 7.18 - 7.15 (m, 8H), 6.86 - 6.82 (m, 8H), 4.38 (h, *J* = 5.3 Hz, 1H), 4.19 – 4.08 (m, 4H), 3.80 (s, 6H), 2.65 (d, *J* = 5.3 Hz, 1H), 1.66 (s, 12H) ppm. The NMR spectra are in agreement with reported data[1].

Ketone III (1-(4-(2-(4-methoxyphenyl)propan-2-yl)phenoxy)propan-2-one)

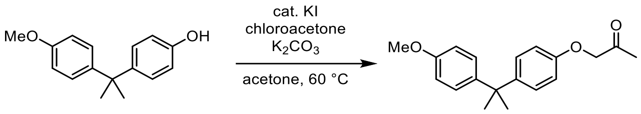

1.21 g (5.0 mmol, 1.0 equiv) of Me-BPA were dissolved in 30 ml of acetone in a round bottom flask. 1.38 g (10.0 mmol, 2.0 equiv) of potassium carbonate, 83.0 mg (0.50 mmol, 0.1 equiv) of potassium iodide and lastly 694 mg (7.50 mmol, 1.5 equiv) of chloroacetone were added and the reaction mixture stirred for 3 h at 60 °C. After the reaction was complete, as confirmed by TLC, it was allowed to cool to rt, and the suspension filtered over a glass frit with acetone. The solvent was removed *in vacuo*. Automated column chromatography using a gradient from heptane 95% / ethyl acetate 5% to heptane 80% / ethyl acetate 20% over silica afforded the product as a colourless oil in a yield of 68 % (1.02 g, 3.42 mmol).

^{1}H NMR ($CDCl_3$, 400 MHz, 25 °C): δ = 7.19 – 7.10 (m, 4H), 6.83 – 6.74 (m, 4H), 4.51 (s, 2H), 3.78 (s, 3H), 2.28 (s, 3H), 1.64 (s, 6H) ppm. The NMR spectra are in agreement with reported data[1].

Lignin model (2-phenoxy-1-phenylethan-1-ol)

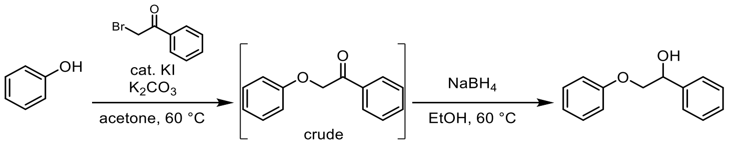

113 mg (1.2 mmol, 1.2 equiv) of phenol were dissolved in 5 ml of acetone in a round bottom flask. 276 mg (2.0 mmol, 2.0 equiv) of potassium carbonate, 16.6 mg (0.10 mmol, 0.1 equiv) of potassium iodide and lastly 199 mg (1.00 mmol, 1.0 equiv) of 2-bromoacetophenone were added and the reaction mixture stirred for over-night at 60 °C. After the reaction was complete, as confirmed by TLC, it was allowed to cool to rt, and the suspension filtered over a glass frit with acetone. The solvent was removed *in vacuo*. The residue was taken up in 10 ml of ethanol, and 75.7 mg (2.0 mmol, 2.0 equiv) of sodium borohydride added in portions at rt with a water bath for cooling. The reaction mixture stirred 10 min at rt and then 1 h at 60 °C. After the reaction was complete, as confirmed by TLC, it was allowed to cool to rt, Celite was added and the solvent removed *in vacuo*. Flash column chromatography using a gradient from pentane / ethyl acetate 20/1 to pentane / ethyl acetate 10/1 over silica afforded the product as a colourless highly viscous oil in a yield of 58 % (125 mg, 0.58 mmol).

^{1}H NMR (toluene-d_8, 400 MHz, 25 °C): δ = 7.30 – 7.24 (m, 2H), 7.19 – 7.12 (m, 2H), 7.12 – 7.04 (m, 3H), 6.81 (dd, *J* = 8.1, 6.8 Hz, 1H), 6.68 – 6.63 (m, 2H), 4.84 – 4.80 (m, 1H), 3.69 – 3.63 (m, 2H) ppm. ppm. The NMR spectra are in agreement with reported data[3].

Model 2 (1-(4-(2-(4-methoxyphenyl)propan-2-yl)phenoxy)-3-(phenethylamino)propan-2-ol)

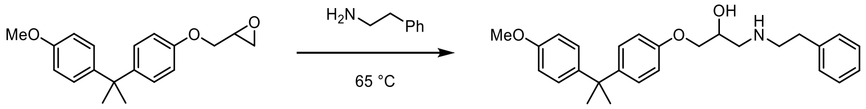

model 2

In a round bottom flask under air, 307 mg (1.03 mmol, 1 equiv) of 2-((4-(2-(4-methoxyphenyl)propan-2-yl)phenoxy)methyl)oxirane were mixed with 1.30 ml (1.25 g, 10.3 mmol, 10 equiv) of 2-phenylethan-1-amine and stirred at 65 °C over-night, then cooled to room temperature. The residue was taken up in 20 ml of ethyl acetate and washed five times with 10 ml of brine, dried over $MgSO_4$ and filtered. The solvent volume was reduced to a few ml *in vacuo,* overlayered with pentane and then stored in a freezer at -30 °C. The product precipitated as colourless solid over the course of a few days. The mother liquor

was decanted off and the solid washed with pentane, affording the product in a yield of 65% (282 mg, 0.67 mmol).

^{1}H NMR (400 MHz, DCM-*d*$_2$): δ 7.31 – 7.26 (m, 2H), 7.23 – 7.17 (m, 3H), 7.14 – 7.10 (m, 4H), 6.82 – 6.75 (m, 4H), 3.97 (dtd, *J* = 10.8, 5.8, 3.0 Hz, 1H), 3.93 – 3.89 (m, 2H), 3.76 (s, 3H), 3.00 – 2.69 (m, 6H), 1.62 (s, 6H).; ^{13}C NMR ($CDCl_3$, 101 MHz, 25 °C): δ = 157.9 (s, 1C), 157.0 (s, 1C), 143.9 (s, 1C), 143.5 (s, 1C), 140.4 (s, 1C), 129.1(d, 2C), 128.8 (d, 2C), 128.1 (d, 2C), 128.0 (d, 2C), 126.5 (d, 1C), 114.2 (d, 2C), 113.5 (d, 2C), 70.9 (t, 1C) , 68.4 (d, 1C), 55.5 (q, 1C), 51.9 (t, 1C), 51.3 (t, 1C), 41.9 (s, 1C), 36.6 (t, 1C), 31.1 (q, 2C) ppm; HRMS (ESI+): calculated $[M+H]^+$ = $[C_{27}H_{33}NO_3+H]^+$ 420.2533; found 420.2611.

2.2 Catalytic deconstructions

General Procedure

For catalytic deconstruction reactions, 43.3 mg (0.08 mmol, 1 equiv) of **model 1** *or* 23.9 mg (0.08 mmol, 1 equiv) of **ketone III** were dissolved in 0.2 ml of toluene-*d*$_8$ and 18.3 µl (14.4 mg, 0.24 mmol, 3 equiv) of isopropanol, then 3 mol% (2.40 µmol) catalyst were added. After sealing the reaction vessel, the mixtures were stirred outside of the glovebox in an aluminium heating block at 160 °C. After the given reaction time (16 h as standard reaction time), 1,3,5-trimethoxybenzene was added to the reaction mixture under argon. Yields were determined by ^{1}H NMR and ^{31}P NMR spectroscopy of the crude mixture with 1,3,5-trimethoxybenzene as internal standard in toluene-*d*$_8$. GC-MS was used to confirm the products detected *via* ^{1}H NMR spectroscopy for all entries.

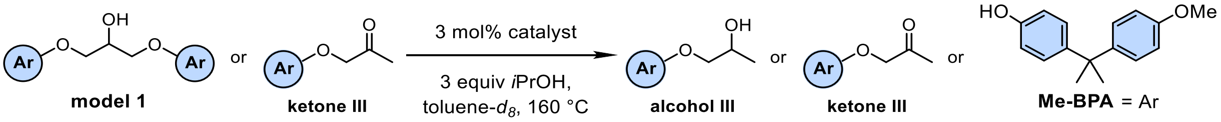

Compounds monitored in reaction mixture:

model 1 ^{1}H NMR (toluene-*d*$_8$, 400 MHz, 25 °C): δ = 7.10 – 7.06 (m, 8H), 6.77 – 6.67 (m, 9H), 4.01 – 3.96 (m, 1H), 3.58 – 3.46 (m, 4H), 3.36 (s, 6H), 1.56 (s, 12H) ppm.

ketone III[1] ^{1}H NMR (toluene-*d*$_8$, 400 MHz, 25 °C): δ = 7.07 – 7.01 (m, 4H), 6.72 – 6.70 (m, 2H), 6.61 – 6.59 (m, 2H), 3.96 (s, 2H), 1.80 (s, 3H), 1.55 (s, 6H) ppm.

alcohol III ^{1}H NMR (toluene-*d*$_8$, 400 MHz, 25 °C): δ = 7.11 – 7.07 (m, 4H), 6.74 – 6.68 (m, 4H), 3.96 – 3.91 (m, 1H), 3.58 – 3.49 (m, 2H), 1.59 (s, 6H), 1.09 (d, J = 6.4 Hz, 3H) ppm.

Me-BPA[1] ^{1}H NMR (toluene-*d*$_8$, 400 MHz, 25 °C): δ = 7.11 – 7.03 (m, 4H), 6.85 – 6.78 (m, 2H), 6.72 – 6.65 (m, 2H), 3.37 (s, 3H), 1.55 (s, 6H) ppm.

1,3,5-trimethoxybenzene ^{1}H NMR (toluene-*d*$_8$, 400 MHz, 25 °C): δ = 6.13 (s, 3H), 3.35 (s, 9H) ppm.

For measuring kinetic profiles, **model 1** was reacted nine times with different reaction times each. 43.3 mg (80.0 µmol, 1 equiv, as stock solution) of **model 1** and 0.2 ml of toluene-*d*$_8$ were given into a 10 ml COtube in an Argon charged glovebox. Potential additives were also added in that stock solution. 3 mol% (1.87 mg, 2.40 µmol) of triphos-Ru-TMM and 3 equiv (14.4 mg, 18.3 µl, 240 µmol) of isopropanol were added. After sealing the reaction vessel, the mixtures were stirred at 650 rpm outside of the glovebox in aluminium heating blocks at 160 °C. After the given reaction time, the reaction was cooled using a water/ice bath. Then, 1,3,5-trimethoxybenzene (as stock solution in toluene-*d*$_8$) was added to the reaction mixture. Yields were determined by ^{1}H NMR spectroscopy of the reaction mixture with 1,3,5-trimethoxybenzene as internal standard. GC-MS was used to confirm the products detected *via* ^{1}H NMR spectroscopy for all entries.

Deconstruction of Model 2

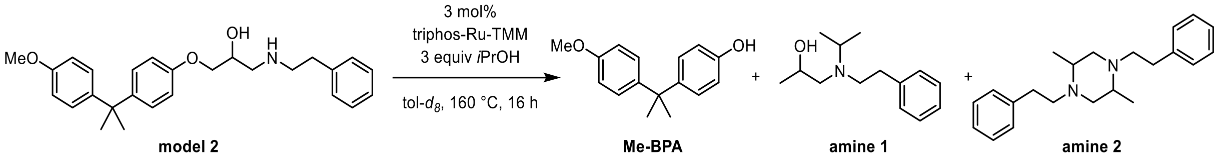

model 2 **Me-BPA** **amine 1** **amine 2**

In an argon-charged glovebox in a J Young NMR tube, 67.1 mg (0.16 mmol, 1 equiv) of **model 2** and 3.74 mg (4.80 µmol, 3 mol%) of triphos-Ru-TMM were dissolved in 0.5 ml of toluene-d_8 and 36.7 µl (0.48 mmol, 3 equiv) of isopropanol. The tube was sealed and heated in an oil bath to 160 °C for 16 h, after which full conversion was observed by NMR spectroscopy. The solvent was removed *in vacuo* and the products mixture submitted to automated flash column chromatography over a 4 g silica column using a gradient from heptane to ethyl acetate to methanol.

Me-BPA[1] eluted at 40% heptane 60% ethyl acetate and was isolated as an off white oil in a yield of 84% (32.6 mg, 135 µmol): ^{1}H NMR (toluene-d_8, 400 MHz, 25 °C): δ = 7.11 – 7.03 (m, 4H), 6.85 – 6.78 (m, 2H), 6.72 – 6.65 (m, 2H), 3.37 (s, 3H), 1.55 (s, 6H) ppm.

A mixture of **amine 1** and **amine 2** eluted at 10% ethyl acetate and 90% methanol as a pale brown oil in yields of 8% (2.74 mg, 12.4 µmol) of **amine 1** and 34% (8.75 mg, 80.0 µmol) of **amine 2.** Respective yields determined by ^{1}H NMR spectroscopy.

amine 1 ^{1}H NMR ($CDCl_3$, 400 MHz, 25 °C): δ = 7.33 – 7.27 (m, 2H), 7.23 – 7.19k (m, 3H), 4.22 (dt, *J* = 7.0, 6.1 Hz, 1H), 3.16 (dd, J = 8.6, 6.2 Hz, 1H), 2.98 – 2.95 (m, 2H), 2.67 – 2.64 (m, 2H), 2.57 (dd, *J* = 8.6, 7.1 Hz, 1H), 1.27 (d, *J* = 6.1 Hz, 4H), 1.19 (d, *J* = 8.1 Hz, 6H) ppm; ^{13}C NMR (101 MHz, $CDCl_3$, 25 °C): δ = 140.5 (s 1C), 128.7 (d, 2C), 128.5 (d, 2C), 126.2 (d, 1C), 71.1 (d, 1C), 65.2 (d, 1C), 57.2 (t, 1C), 51.4 (t, 1C), 50.7, 36.5 (d, 1C), 24.0 (q 1C), 21.1 (q, 2C) ppm; HRMS (ESI+): calculated [M+*i*PrOH +H]$^+$ = [$C_{14}H_{23}NO$+*i*PrOH+H]$^+$ 282.2433; found 282.2794.

amine 2 ^{1}H NMR ($CDCl_3$, 400 MHz, 25 °C): δ = 7.33 – 7.27 (m, 4H), 7.24 – 7.18 (m, 6H), 3.79 (dqd, *J* = 9.4, 6.2, 3.0 Hz, 2H), 2.98 – 2.86 (m, 4H), 2.85 – 2.79 (m, 4H), 2.73 (dd, *J* = 12.1, 3.0 Hz, 2H), 2.44 (dd, *J* = 12.1, 9.6 Hz, 2H), 1.14 (d, *J* = 6.2 Hz, 6H) ppm; ^{13}C NMR (101 MHz, $CDCl_3$, 25 °C): δ = 139.6 (s, 2C), 128.8 (d, 4C), 128.7 (d, 4C), 126.5 (d, 2C), 65.2 (d, 2C),56.5 (t, 2C), 50.7 (t, 2C), 36.1 (t, 2C), 20.6 (q, 2C) ppm; HRMS (ESI+): calculated [M+2H]$^{2+}$ = [$C_{22}H_{30}N_2$+2H]$^{2+}$ 162.1277; found 162.1279.

2.3 Reactivity of the TMM-precatalyst

For studies on catalyst activation, experiments were set up in an Argon-charged glovebox and conducted in a J Young NMR tube using ^{31}P NMR and ^{1}H NMR spectroscopy. For the first experiment, 3.74 mg (4.80 µmol) of triphos-Ru-TMM were suspended in 0.5 ml of toluene-d_8 and heated to 160 °C for 16 h. No conversion could be observed in the corresponding ^{31}P NMR or ^{1}H NMR spectra.

For the second experiment, 3.74 mg (4.80 µmol) of triphos-Ru-TMM were suspended in 0.5 ml of toluene-d_8, 36.8 µl (24.8 mg, 0.48 mmol) of isopropanol were added, and the mixture heated to 160 °C for 16 h. No consumption of the complex could be observed in the corresponding ^{31}P NMR or ^{1}H NMR spectra.

For the third experiment, 86.6 mg (0.16 mmol, 1 equiv) of **model 1** were dissolved in 0.5 ml of toluene-d_8, 3.74 mg (4.80 µmol, 3 mol%) of triphos-Ru-TMM were added, and the mixture heated to 160 °C for 16 h. No consumption of the complex or starting material could be observed in the corresponding ^{31}P NMR or ^{1}H NMR spectra.

For the fourth experiment, 86.6 mg (0.16 mmol, 1 equiv) of **model 1** were dissolved in 0.5 ml of toluene-d_8, 36.8 µl (24.8 mg, 0.48 mmol, 3 equiv) of isopropanol and 3.74 mg (4.80 µmol, 3 mol%) of triphos-Ru-TMM were added, and the mixture heated to 160 °C for 16 h. Full consumption of the complex and formation of new species were observed in the corresponding ^{31}P NMR spectrum (Supplementary Figure 2), including deactivated ruthenium complexes[1]. **Model 1** was also completely consumed under formation of <99% of **Me-BPA**, as determined by 1H NMR spectroscopy using 1,3,5-trimethoxybenzene as internal standard.

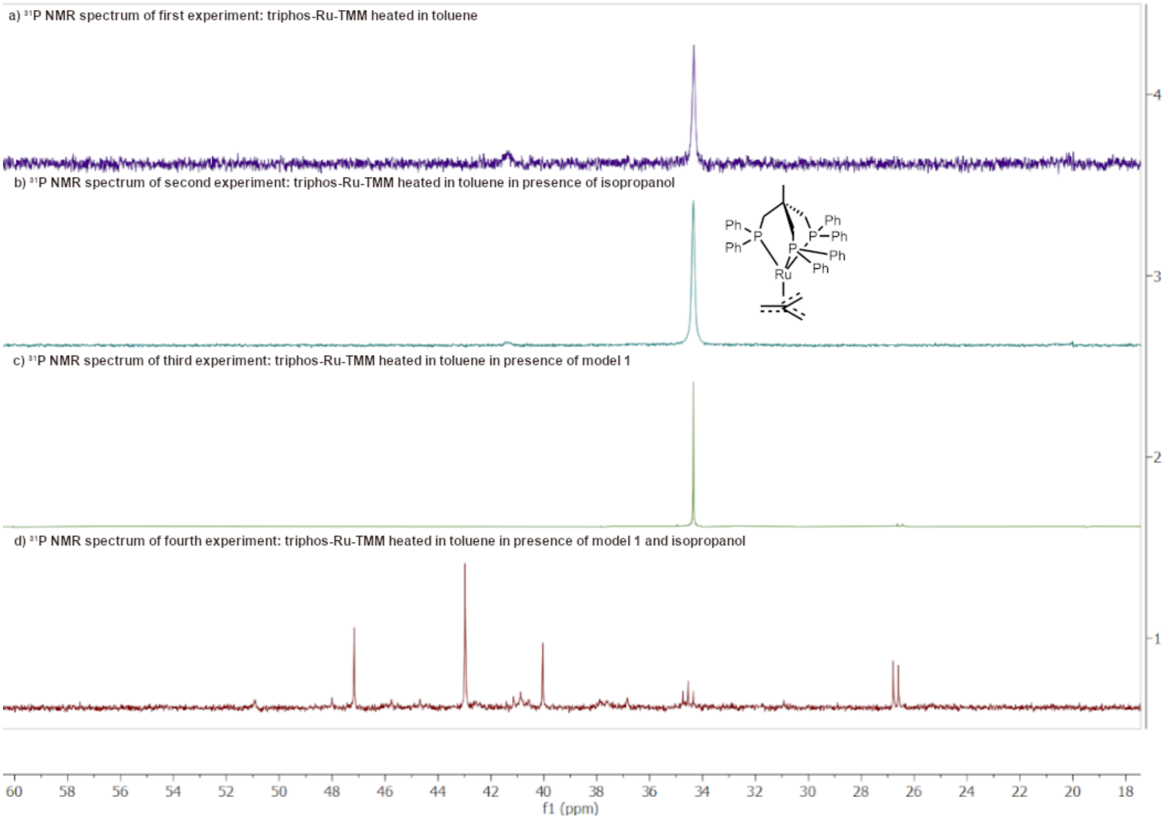

Supplementary Figure 2 | ^{31}P NMR spectra after reacting triphos-Ru-TMM in toluene-d_8 at 160 °C for 16 h. in presence of different compounds. **a,** Complex heated in toluene. **b,** Complex heated in toluene and isopropanol. **c,** Complex heated in toluene together with the epoxy model compound. **d,** Complex heated in toluene and isopropanol together with the epoxy model compound. *TMM* trimethylenemethane, *triphos* 1,1,1-tris(diphenyl-phosphinomethyl)ethane

For the reaction with gold salts: In a J Young NMR tube, 9.75 mg (12.5 µmol, 1.0 equiv) of triphos-Ru-TMM was dissolved in 0.5 mol of THF-d_8. 16.2 mg (18.7 µmol, 1.5 equiv) of $IPrAuNTf_2$ was added and the mixture left the react at rt. The reaction was monitored using ^1H and ^{31}P NMR spectroscopy. After 1 h at rt two new species had formed, each with a singlet peak at 48.4 ppm and 38.9 ppm, respectively, in the ^{31}P NMR spectrum. Residues of triphos-Ru-TMM could be observed at 34.7 ppm. Addition of another 16.2 mg (18.7 µmol, 1.5 equiv) of $IPrAuNTf_2$ and another hour of reaction time resulted in complex with a peak at 48.4 ppm being the main species (Supplementary Figure 3).

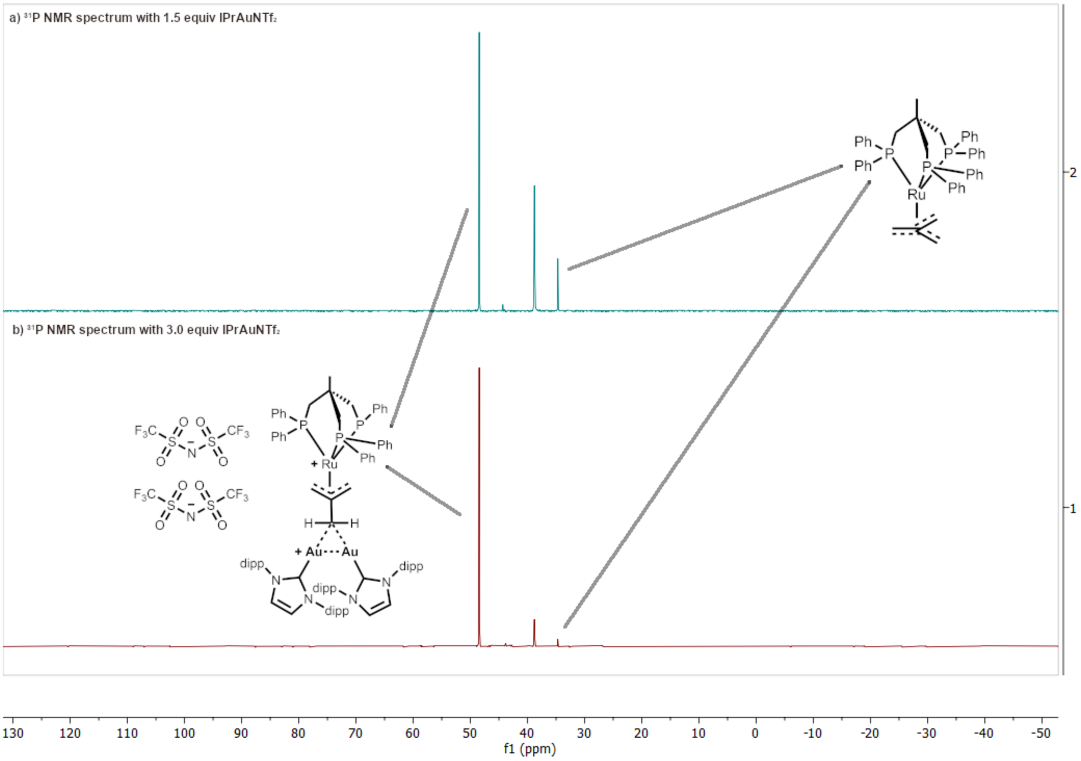

Supplementary Figure 3 | ^1H NMR and ^{31}P NMR spectrum after reacting triphos-Ru-TMM with $IPrAuNTf_2$. a, Reacted with 1.5 equiv $IPrAuNTf_2$ in THF-d_8. ***b,*** Reacted with and 3.0 equiv $IPrAuNTf_2$ in THF-d_8. *dipp* 2,6-diisopropylphenyl, *TMM* trimethylenemethane, *triphos* 1,1,1-tris(diphenyl-phosphinomethyl)ethane

The structure corresponding to the ^{31}P NMR signal at 48.4 ppm is proposed to be a diaurated 2-methylleneallyl ruthenium complex. This conclusion was drawn from several considerations: a) A singlet phosphine signal rules out a complexation of one of the phosphines by a gold centre. b) triphos-Ru-TMM was reacted with one equiv of $HNTf_2$ in THF-d_8, forming the structurally related [triphos-Ru-(2-methylallyl)]NTf_2 complex, which displays a similar chemical shift at 48.6 ppm in its ^{31}P NMR spectrum. c) That the methylene is diaurated and not mono aurated was concluded from the shift towards a main species as the ratio of gold salt exceeded 2 equiv., with both IPr fragments having equivalent chemical environments. The ^{31}P NMR signal at 38.9 ppm could potentially correspond to an intermediary mono aurated complex. Diaurated species are often favourable due to aurophilic interactions often observed for gold(I) complexes[4].

Attempts to grow crystalls suitable for X-Ray crystallography failed due to decomposition.

Reacting the diaurated species with KI in THF-d_8 at rt led to an immediate and quantitative regeneration of triphos-Ru-TMM.

When 9.75 mg (12.5 µmol, 1.0 equiv) of triphos-Ru-TMM was reacted with 32.4 mg (37.4 µmol, 3 equiv) of $IPrAuNTf_2$ in 0.5 ml of THF-d_8 under 1 atm of CO (released *ex situ* in a two-chamber set up from SilaCOgen[5]) at rt. Here, a colourless solution was obtained within 15 min of reaction times, which started to turn grey under formation of nanoparticles over hours at rt. Analysis by ^{31}P NMR spectroscopy

revealed two discreet species. Over two days, one of the species decayed under formation of elemental metal (Supplementary Figure 4).

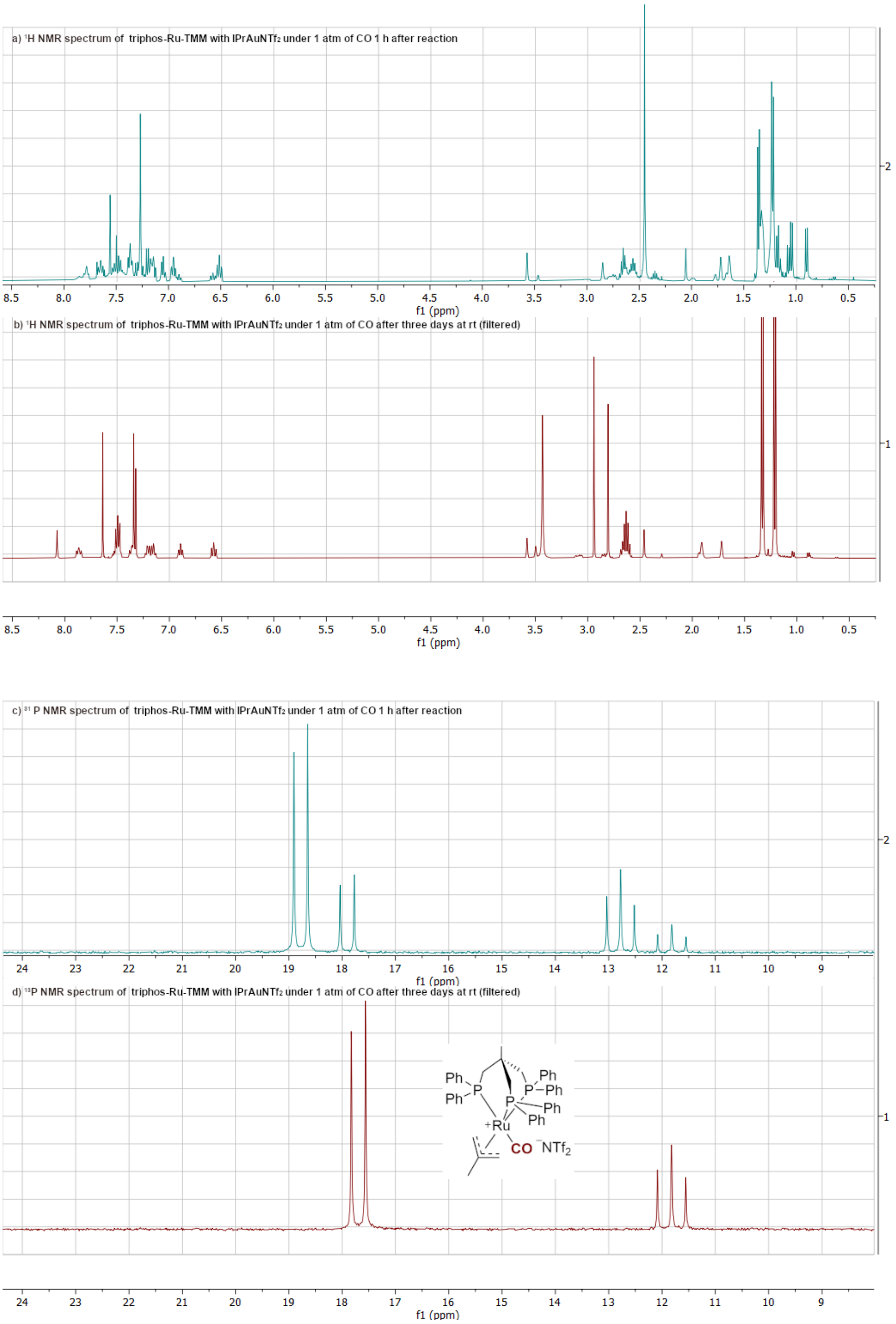

Supplementary Figure 4 | Stacked NMR spectra of triphos-Ru-TMM reacted with 3.0 equiv IPrAuNTf₂. under 1 atm of CO, and decomposition of species in THF-*d*₈. a, ^{1}H NMR spectrum after 1 h reaction time. **b,** ^{1}H NMR spectrum after decomposition of aurated species. **c,** ^{13}P NMR spectrum after 1 h reaction time. **d,** ^{13}P NMR spectrum after decomposition of aurated species.

Carbonyl ligand marked red. *dipp* 2,6-diisopropylphenyl, *TMM* trimethylenemethane, *triphos* 1,1,1-tris(diphenyl-phosphinomethyl)ethane

Here, it was possible to grow single crystals suitable for X-ray crystallography by over layering the crude reaction mixture with pentane (vapor diffusion) in a freezer. However, the structure showed a decomposition product, which nonetheless indirectly supports the proposed structure.

For deuteration experiments, in an argon charged glovebox 6.24 mg (8.00 µmol, 10 mol%) of triphos-Ru-TMM was given into a J Young NMR tube. 5.88 µl (80.0 µmol, 1.0 equiv) of acetone-d_6 and 6.12 µl (80.0 µmol, 1.0 equiv) of isopropanol-d_8 were added together with 0.5 ml of benzene. The reaction mixture was refluxed at 120 °C in an oil bath for 16 h, after which a ^{2}H NMR spectrum was measured, showing a singlet peak at 2.36 ppm, corresponding to the CH_2 group of the TMM ligand. The integrals imply a deuterium incorporation of roughly 85% (determined *via* ^{1}H NMR spectroscopy).

The experiment was repeated using benzene-d_6 as solvent. ^{1}H NMR spectra before and after the reaction confirmed the deuteration of the TMM ligand (Supplementary Figure 5). In both cases the ^{31}P NMR spectra showed no peaks besides the precatalyst.

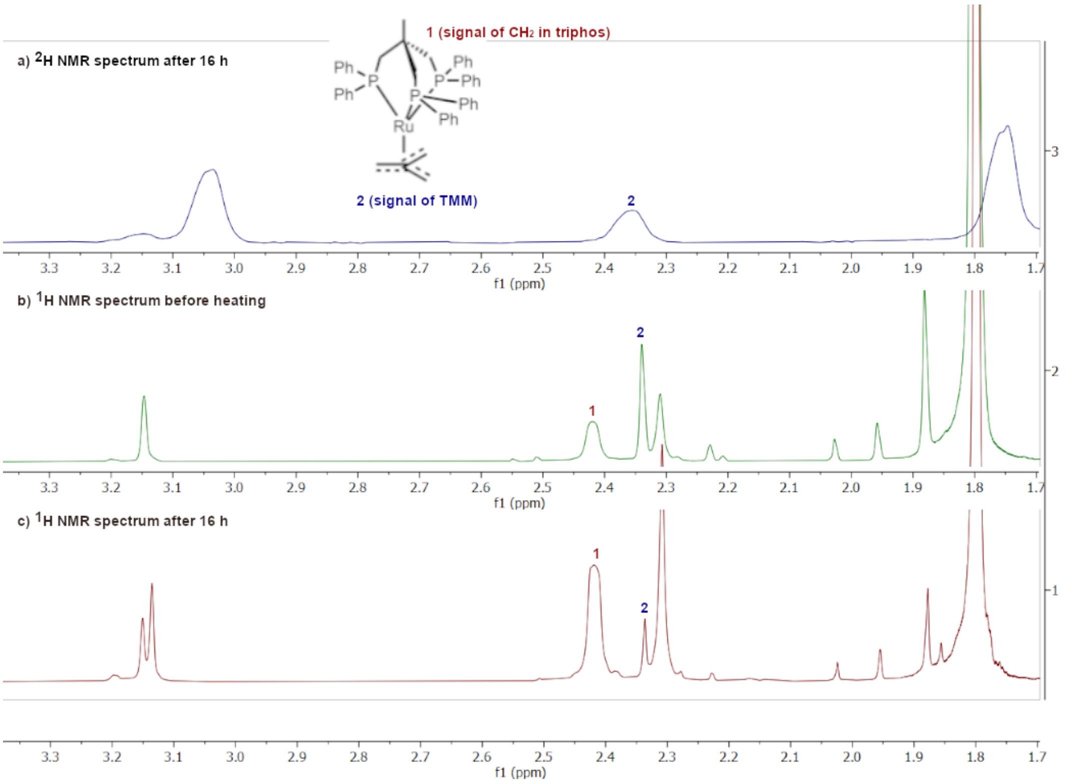

Supplementary Figure 5 | Stacked NMR spectra of reacting triphos-Ru-TMM with isopropanol-*d*$_8$ and acetone-*d*$_6$. a, ^{2}H NMR spectrum after 16 h in benzene. **b,** ^{1}H NMR spectrum before heating in benzene-d_6. **c,** ^{1}H NMR spectrum after heating in benzene-d_6. Proton signals corresponding the TMM ligand marked with a blue 2. Proton signals corresponding to the CH_2 linker in the triphos ligand marked with a red 1. *TMM* trimethylenemethane, *triphos* 1,1,1-tris(diphenyl-phosphinomethyl)ethane

2.4 Role of ruthenium phenolates

Observation of triphos-Ru-(Me-BPA)$_2$

In a J Young NMR tube, 16.1 mg (20.75 µmol, 1.0 equiv) of triphos-Ru-TMM was dissolved in 0.5 mol of toluene-d_8. 20.1 mg (82.9 µmol, 4.0 equiv) of Me-BPA was added and the mixture left the react over-night at 160 °C. The reaction was monitored using ^{31}P NMR spectroscopy. An additional 40.1 mg (166 µmol, 8 equiv) of Me-BPA were added and the mixture left the react over-night at 160 °C. A new main species corresponding to a triphos-Ru-(Me-BPA)$_2$ shows a signal at 49.6 ppm. Two minor signal at 40.5 ppm and 39.9 ppm could not be assigned (Supplementary Figure 6). Attempts to grow crystalls suitable for X-Ray crystallography failed.

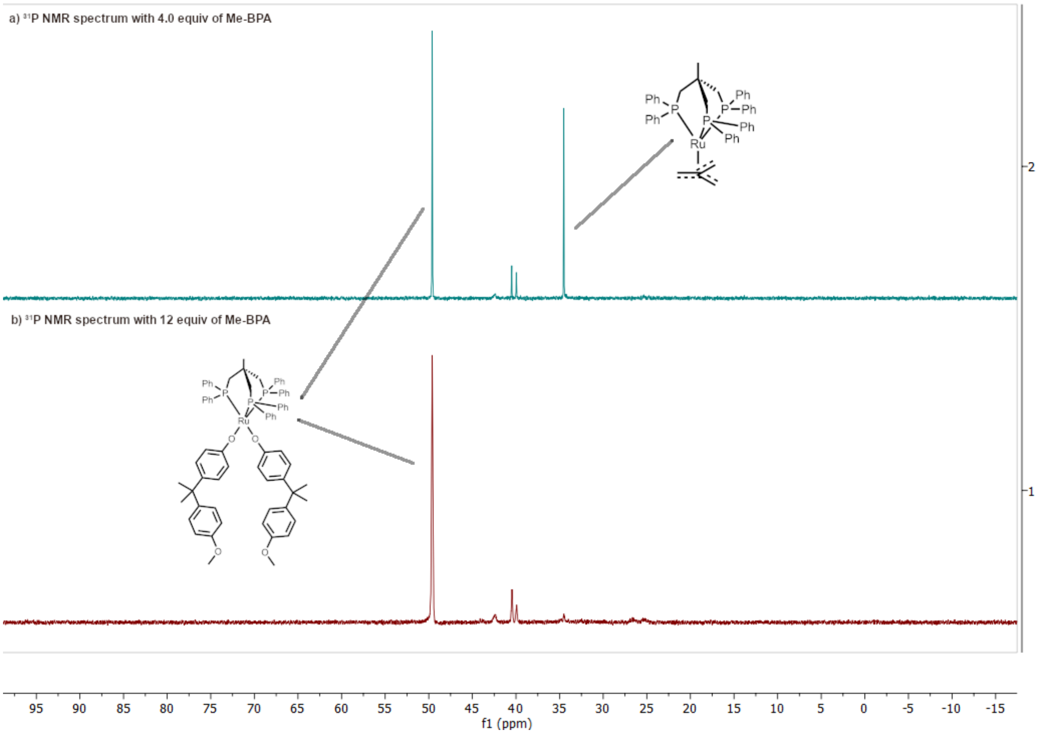

Supplementary Figure 6 | Reaction of triphos-Ru-TMM with an excess of Me-BPA in toluene-d_8. a, ^{31}P NMR spectrum with 4 equiv Me-BPA. **b,** ^{31}P NMR spectrum with 12 equiv Me-BPA. *Me-BPA* O-methyl bisphenol A

Synthesis of Ru-1 (triphos-Ru-(2-fluorophenolate)$_2$)

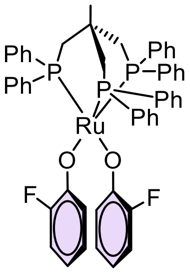

In a J Young NMR tube, 9.75 mg (12.5 µmol, 1.0 equiv) of triphos-Ru-TMM was dissolved in 0.5 mol of toluene-d_8. 4.69 µl (5.61 mg, 50.0 µmol, 4.0 equiv) of 2-fluorophenol was added and the mixture left the react at 60 °C. Over the course of 4 h the starting complex dissolved slowly, yielding a deep red solution. ^{31}P NMR spectroscopy of the reaction mixture revealed a main product with a singlet signal at 49.5 ppm. ^{1}H and ^{13}C NMR spectroscopy allowed the detection of isobutene[6] (Supplementary Figure 7).

Isobutene ^{1}H NMR (toluene-d_8, 400 MHz, 25 °C): δ = 4.72 (sept, J = 1.2 Hz, 2H), 1.60 (t, J = 1.2 Hz, 6H) ppm; ^{13}C NMR (toluene-d_8, 400 MHz, 25 °C): δ = 111.1 (t, 1C), 24.0 (q, 2C) ppm. The quaternary carbon could not be observed.

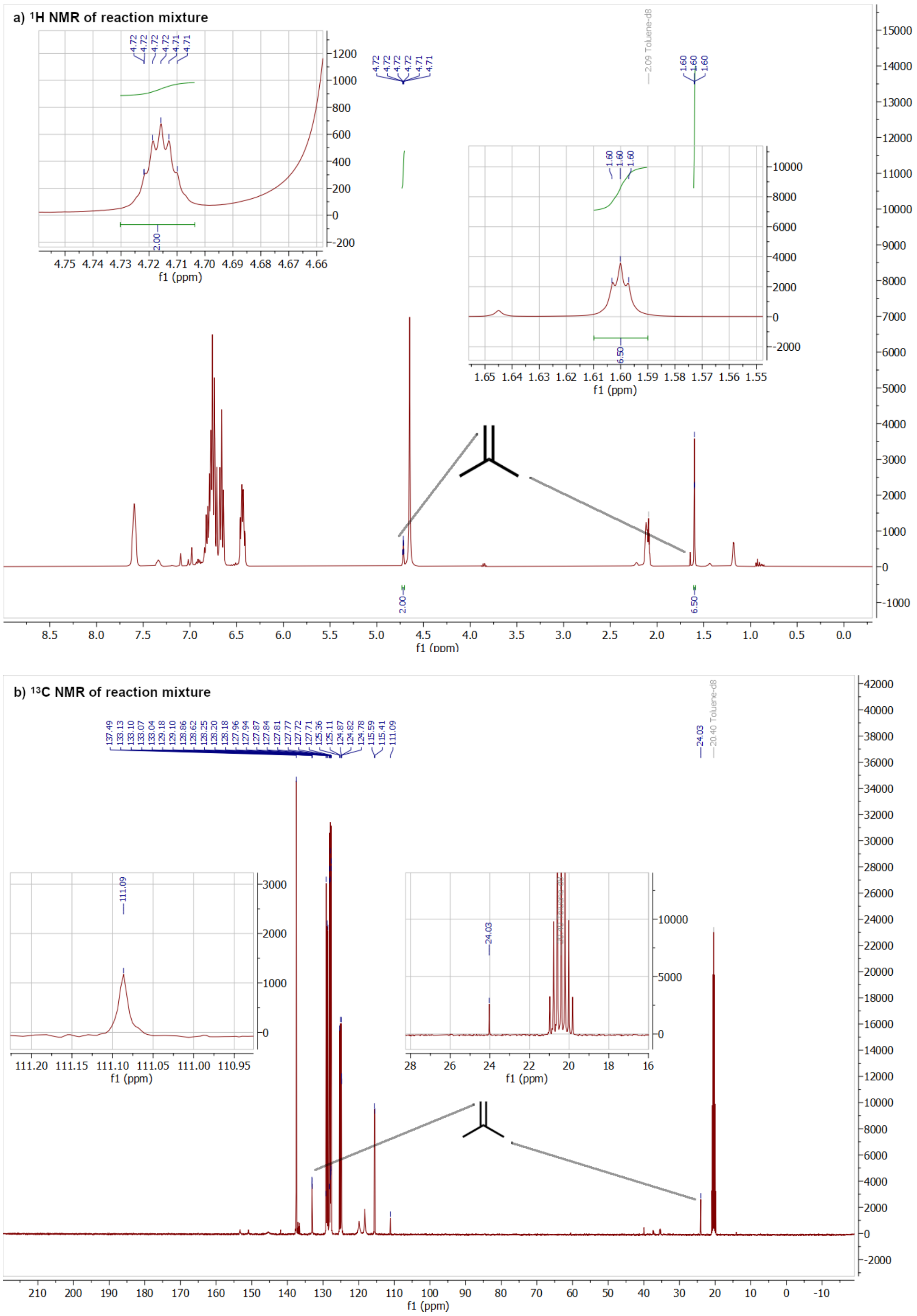

Supplementary Figure 7 | Observation of isobutene in NMR spectra of reaction mixture. **a,** ^{1}H NMR spectrum in toluene-d_8. **b,** ^{13}C NMR spectrum in toluene-d_8.

For the isolation of **Ru-1**, the obtained solution was transferred into a vial in an argon-charged glovebox and overlayered with pentane. The title complex crystallised as an air-sensitive orange solid.

Ru-1 ^{1}H NMR (toluene-d_8, 400 MHz, 25 °C): δ = 7.64 – 7.57 (m, 12H), 6.85 – 6.66 (m, 39H), 6.45 – 6.38 (m, 6H), 2.12 (dd, J = 5.8, 3.0 Hz, 6H), 1.18 (d, J = 2.9 Hz, 3H) ppm; ^{13}C NMR (CD_2Cl_2, 101 MHz, 25 °C): δ = 136.3 (s, 6C), 132.7 (d, 12C), 129.0 (d, 6C), 127.6 (d, 12C), 123.4 (d, 2C), 114.0 (d, 2C),

40.1 (s, 1C), 37.8 (q, 1C). 35.4 (t, 3C) ppm [2 times 4 carbons of the phenolate could not be found]; ^{19}F NMR (toluene-d_8, 376 MHz, 25 °C): δ = - 140.6 ppm; ^{31}P NMR (toluene-d_8, 162 MHz, 25 °C): δ = 49.9 ppm.

Surprisingly, an attempt to take spectra of the complex in THF-d_8 resulted in the spontaneous decomposition of the phenolate, as the colour changed from red to yellow within seconds of dissolving the complex. NMR analysis revealed quantitative formation of a new species with a singlet ^{31}P NMR signal at 50.1 ppm as well as the formation of 2-fluorophenol (- 137.6 ppm in^{19}F NMR spectroscopy[7]) in THF-d_8. Under argon, the solution containing this new species was overlayered with pentane and placed in a freezer, yielding crystals suitable for X-Ray crystallography, revealing a Ru-propionate (Supplementary Figure 8). After taking the measured crystal samples up in THF-d_8 under argon gave a consistent ^{31}P NMR spectrum.

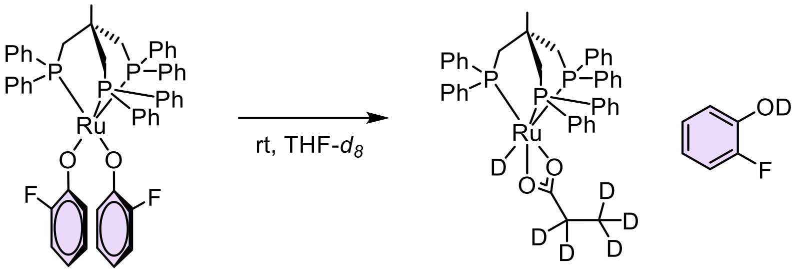

Supplementary Figure 8 | Reaction of **Ru-1** with THF-d_8 yielding **Ru-propionate**.

Transformation of **Ru-1** was also observed in acetone-d_6 under formation of an unidentified species with a with a singlet ^{31}P NMR signal at 40.1 ppm.

Synthesis of Ru-2 (triphos-Ru-(2,6-difluorophenolate)$_2$)

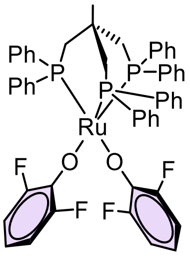

In a 10 ml COtube, 78.0 mg (0.10 mmol, 1.0 equiv) of triphos-Ru-TMM was dissolved in 1 mol of toluene. 52 mg, 0.40 mmol, 4.0 equiv) of 2,6-difluorophenol was added and the mixture left the react for 1 h at 60 °C. The reaction mixture was allowed to cool to room temperature and overlayered with 2 ml of pentane, resulting in orange precipitate. Decanting the solution and washing the precipitate afforded **Ru-2** in a yield of 93% (91.5 mg, 93.0 µmol)

^{1}H NMR (toluene-d_8, 400 MHz, 25 °C): δ = 7.65 – 7.64 (m, 12H), 6.86 – 6.79 (m, 18H), 6.60 – 6.56 (m, 4H), 6.05 – 5.98 (m, 2H), 2.12 (s, 6H), 1.15 (d, J = 3.0 Hz, 3H) ppm; ^{31}P NMR (toluene-d_8, 162 MHz, 25 °C): δ = 49.8 (s, 3P) ppm; ^{19}F NMR (toluene-d^8, 376 MHz, 25 °C): δ = - 133.8 (s, 4F) ppm.

^{1}H NMR (toluene-d_8, 400 MHz, 25 °C): δ = 7.65 – 7.64 (m, 12H), 6.86 – 6.79 (m, 18H), 6.60 – 6.56 (m, 4H), 6.05 – 5.98 (m, 2H), 2.12 (s, 6H), 1.15 (d, J = 3.0 Hz, 3H) ppm; ^{13}C NMR (CD_2Cl_2, 101 MHz, 25 °C): δ = 156.7 (s_{CH}, dd_{CF}, J_{CF} = 237, 10.0 Hz, 4C), 145.2 (s_{CH}, t_{CF}, J_{CF} = 15.0 Hz, 2C), 136.4 (s, 6C), 133.0 (d, 12C), 129.4 (d, 6C), 127.8 (d, 12C), 110.4 - 110.1 (d, 6C), 40.0 (s, 1C), 37.5 (q, 1C), 35.5 - 35.2 (t, 3C) ppm; ^{31}P NMR (toluene-d_8, 162 MHz, 25 °C): δ = 49.8 (s, 3P) ppm; ^{19}F NMR (toluene-d_8, 376 MHz, 25 °C): δ = - 133.8 (s, 4F) ppm.

Reacting Ru-1 with isopropanol

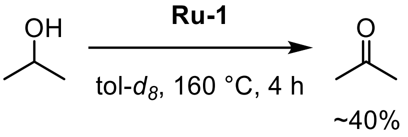

In an argon-charged glovebox in a J Young tube, 8.00 mg (0.008 mmol, 1 equiv) of **Ru-1** were suspended in 0.5 ml of toluene-*d₈*. 12.4 µl (9.77 mg, 0.06 mmol, 20 equiv) of isopropanol were added. The reaction mixture was heated to 160 °C for 4 h. ^{1}H NMR spectroscopy revealed that approximately 40% of isopropanol had been converted to acetone (Supplementary Figure 9), while ^{31}P NMR spectroscopy showed no new species formed from **Ru-1**.

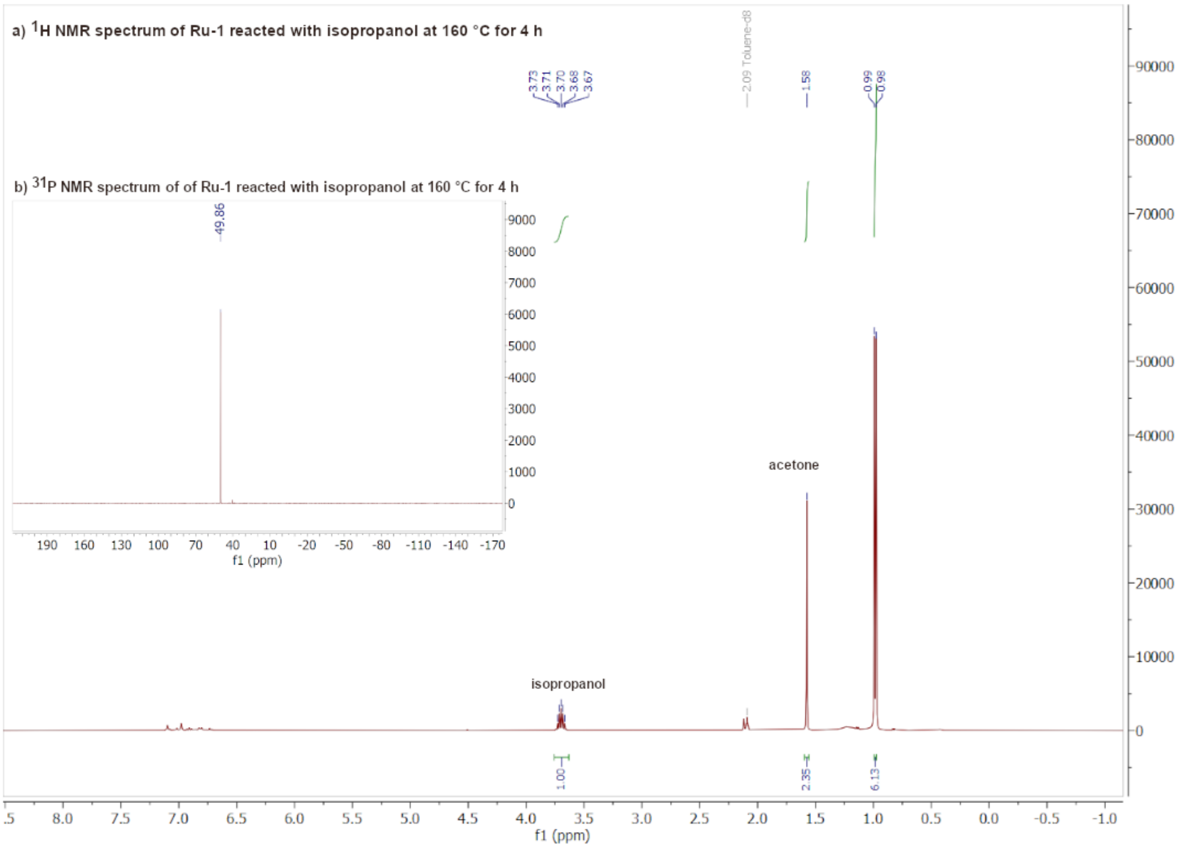

Supplementary Figure 9 | NMR spectra of Ru-1 reacted with isopropanol. a, ^{1}H NMR spectrum in toluene-*d₈*. **b,** ^{31}P NMR spectrum in toluene-*d₈*.

Reaction of Ru-2 with potassium 1-phenylethanolate

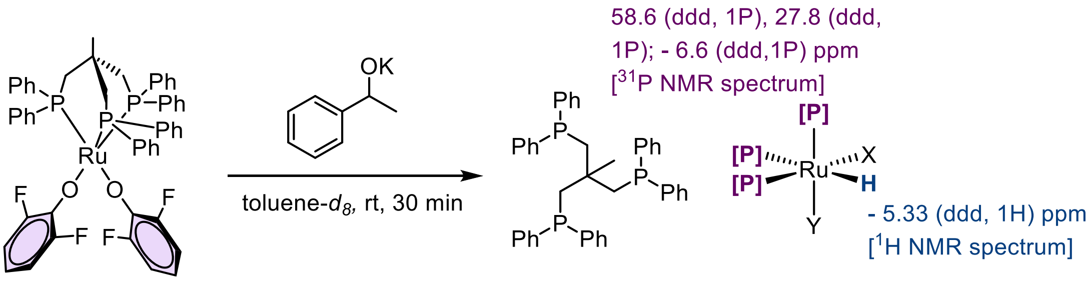

In an argon-charged glovebox in a J Young tube, 14.8 mg (0.015 mmol, 1 equiv) of **Ru-2** were suspended in 0.5 ml of toluene-*d₈*. 9.62 mg (0.06 mmol, equiv) of potassium 1-phenylethan-1-olate were added. Over the course of 30 min **Ru-2** dissolved under occasional shaking of the NMR tube. The

colour changed rapidly to a pale brown solution. Multicore and 2D NMR spectroscopy (Supplementary Figure 10) revealed the ruthenium hydrides species **Ru-H**. Attempts to isolate the complex from the reaction mixture or growing crystals suitable for analysis by X-Ray crystallography failed due to decay of the species so far.

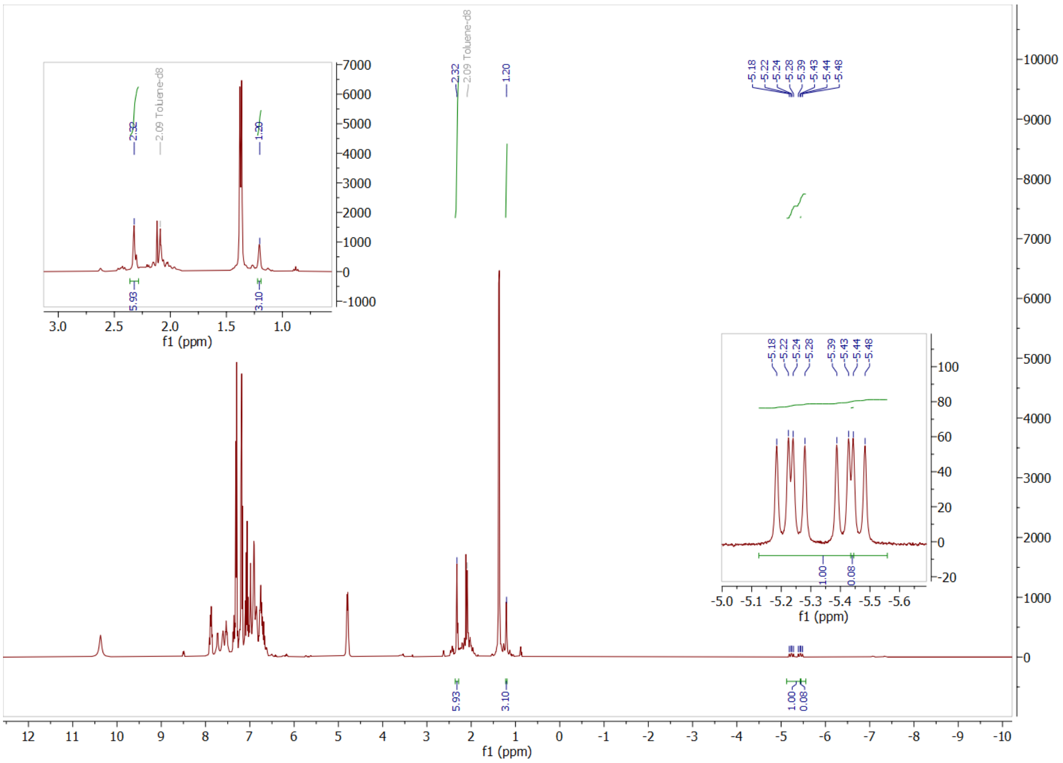

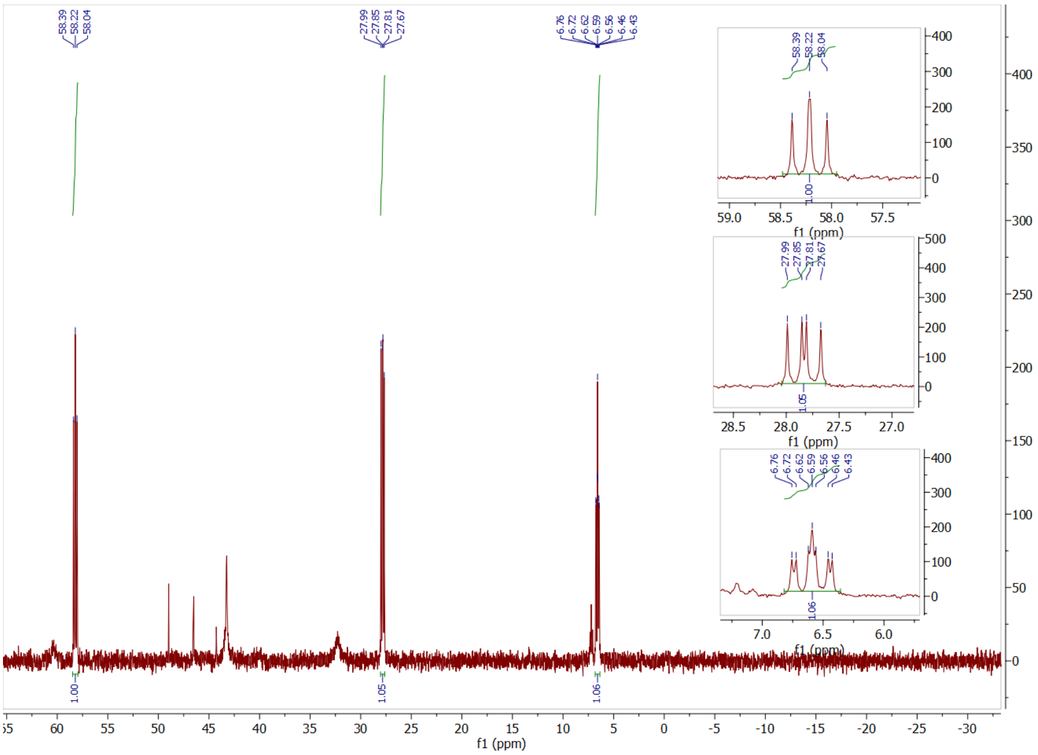

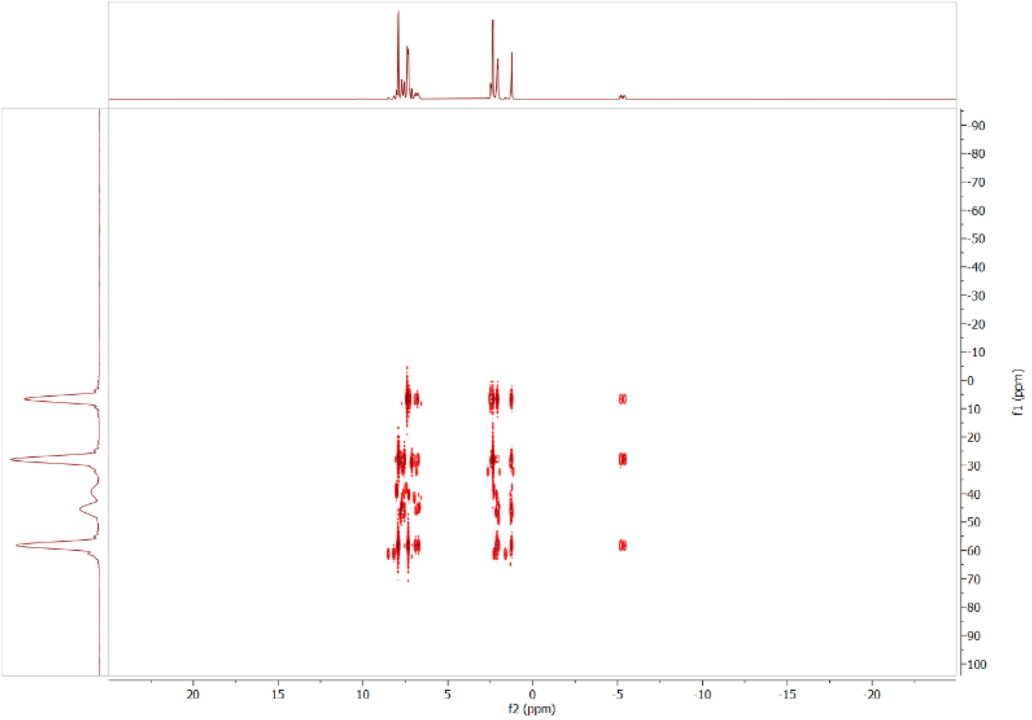

Supplementary Figure 10 | ^{1}H NMR, ^{31}P NMR and ^{1}H, ^{31}P HMBC spectra confirming the structure of **Ru-H** in THF-d_8.

Synthesis of I7^{xyl}

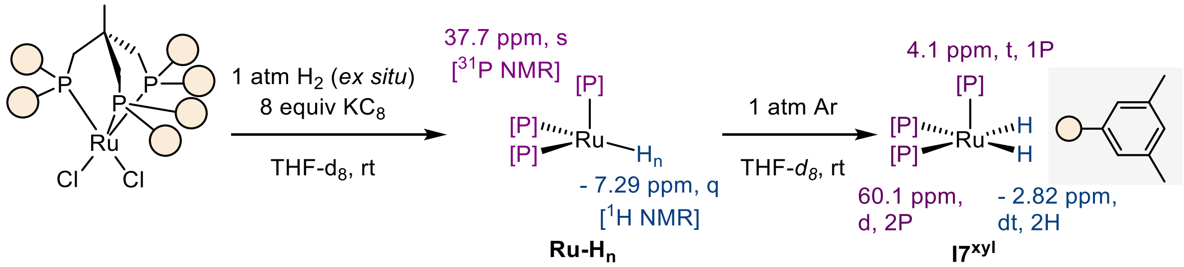

Through Ru(0): In an argon-charged glovebox in a 20 ml two chamber set up, 9.65 mg (5.00 µmol, 1 equiv) of triphos^{xyl}-Ru-Cl_2 and 5.41 mg (40.0 µmol, 8 equiv) of KC_8 were suspended in 0.5 ml of THF-d_8 in chamber A. In chamber B 1 atm of dihydrogen was released by suspending 48.4 mg of zinc in 1 ml of ethylene glycol and adding 0.24 ml of 6 M hydrochloric acid.[9] The reaction mixture was stirred at rt for 3 h, resulting in a deep black solution. The reaction mixture was filtered into a J Young tube through a pipette packed with Celite. Initial NMR analysis showed **Ru-H$_n$** as major product, which slowly converted to **I7^{xyl}** over the course of hours to days.

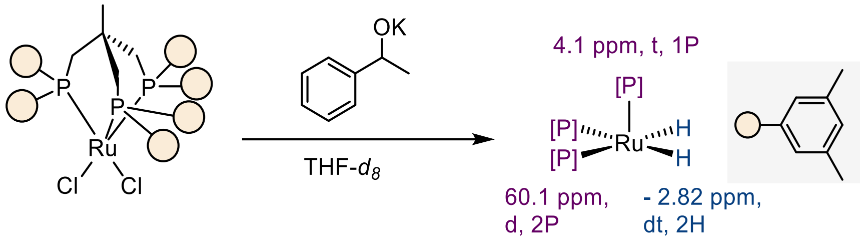

Through β hydride elimination: In an argon-charged glovebox in a J Young tube, 10.2 mg (5.30 µmol, 1 equiv) of triphos^{xyl}-Ru-Cl_2 and 3.40 mg (21.2 µmol, 4 equiv) of potassium 1-phenylethan-1-olate were

suspended in 0.5 ml of THF-d_8 and heated to 80 °C in an oil bath for 4 h, resulting in a turbid red solution. triphos^{xyl}-Ru-H_2-CO was detected as a minor side product.

Crystals suitable for X-Ray crystallography could not be grown successfully so far.

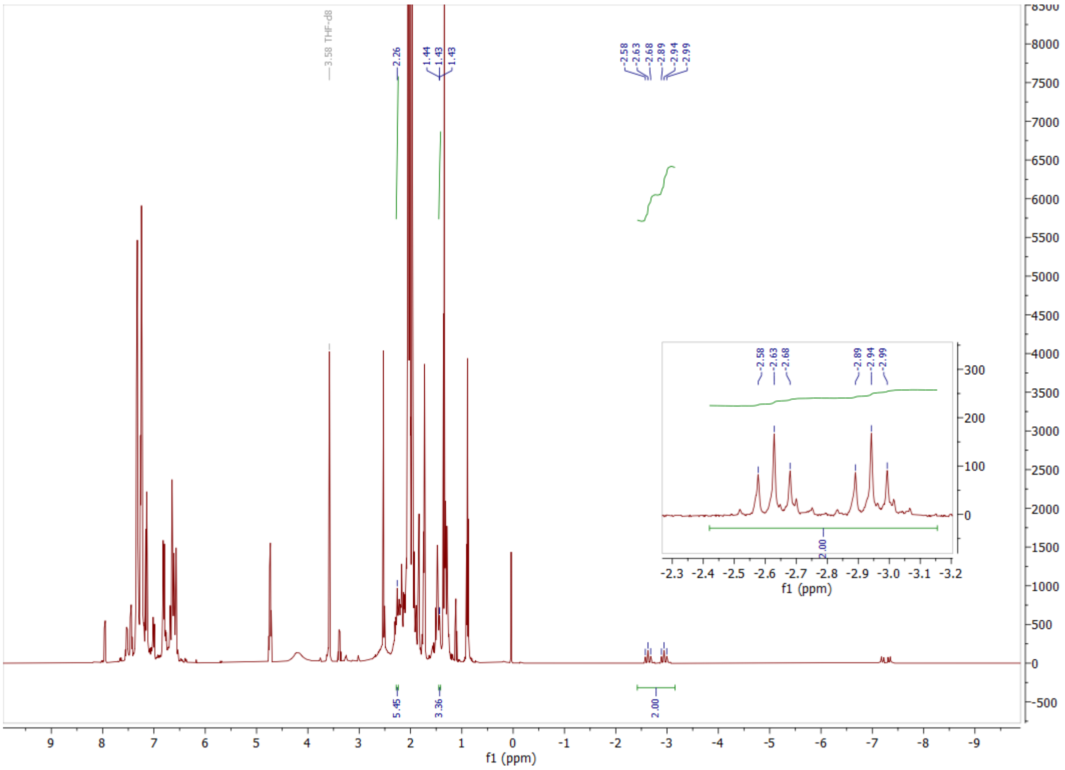

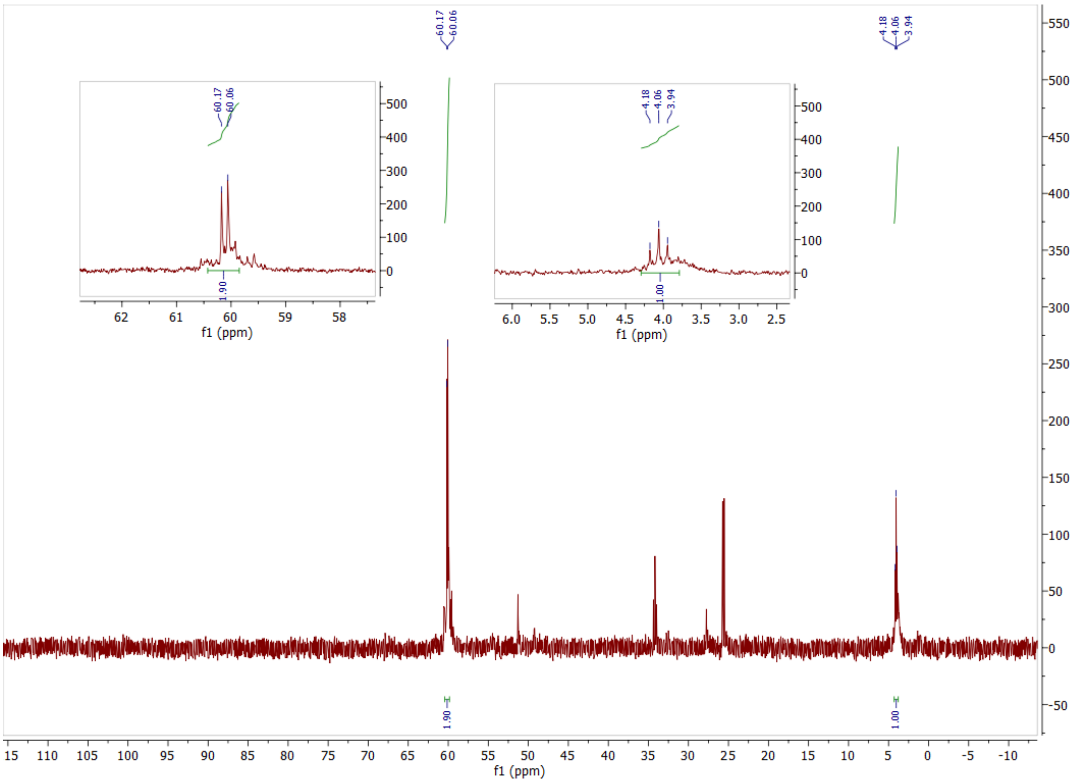

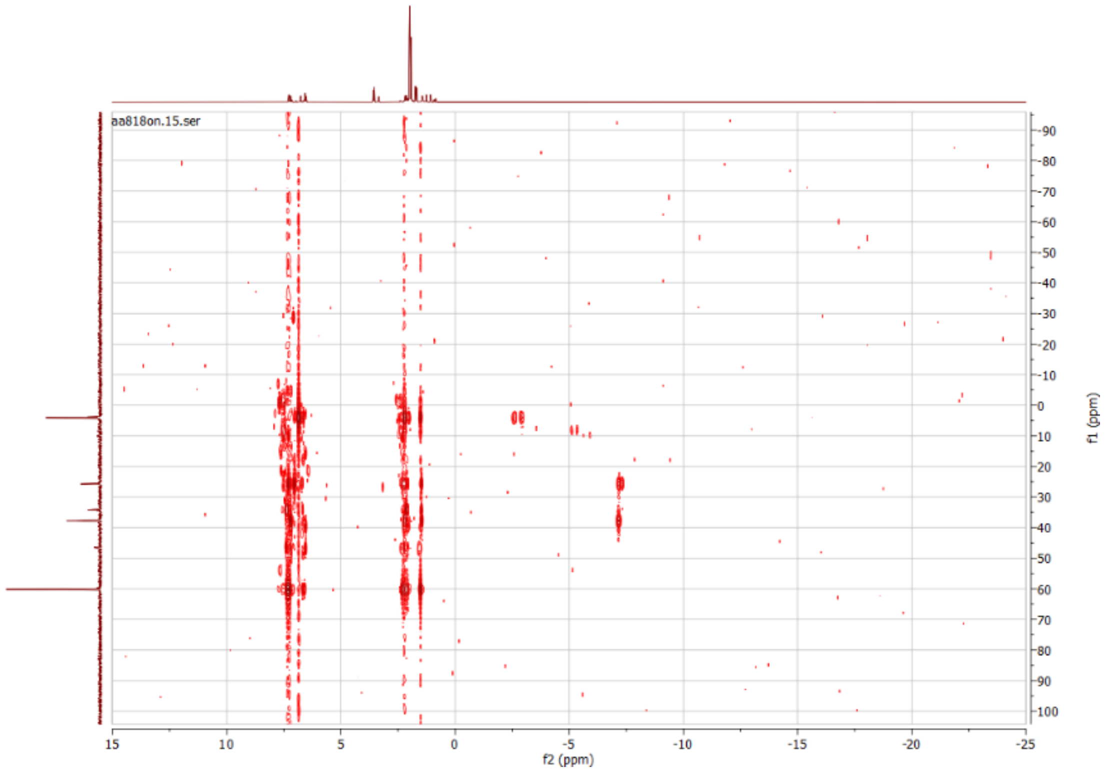

Supplementary Figure 11 | ^{1}H NMR, ^{31}P NMR and ^{1}H, ^{31}P HMBC spectra of **I7^{xyl}** in THF-d_8.

3. NMR Spectra

Diaurated 2-methylleneallyl ruthenium complex

a) ^{1}H NMR spectrum in THF-d_8

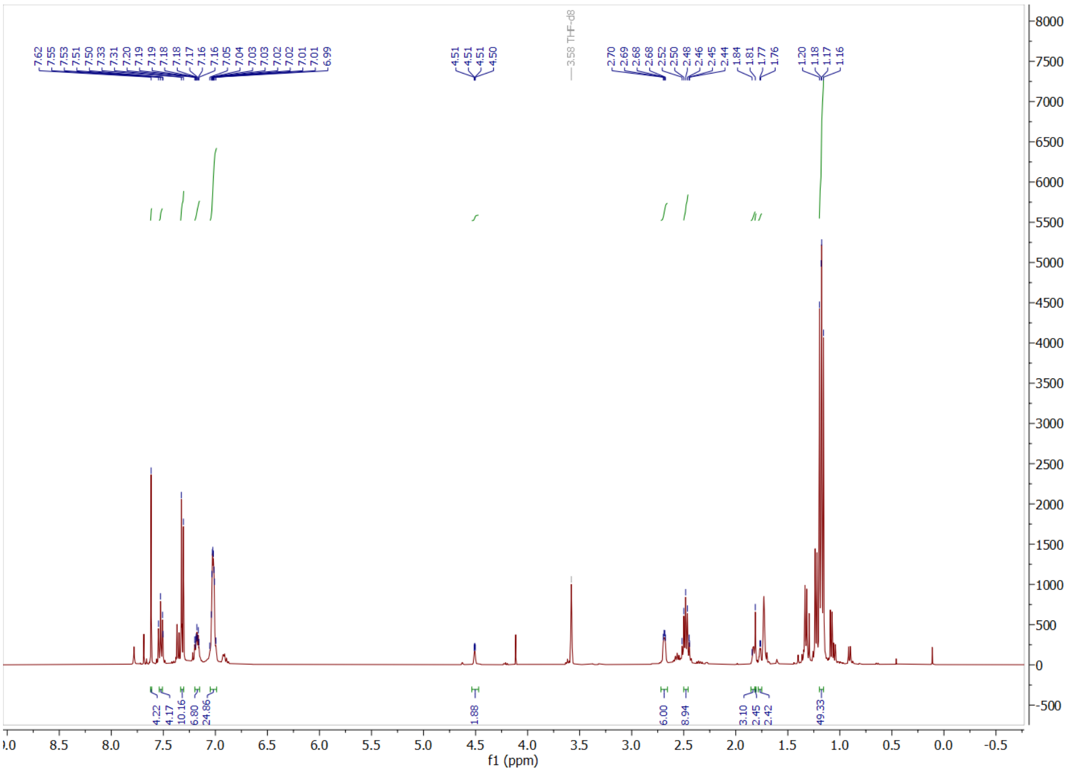

b) ^{13}C APT NMR spectrum in THF-d_8

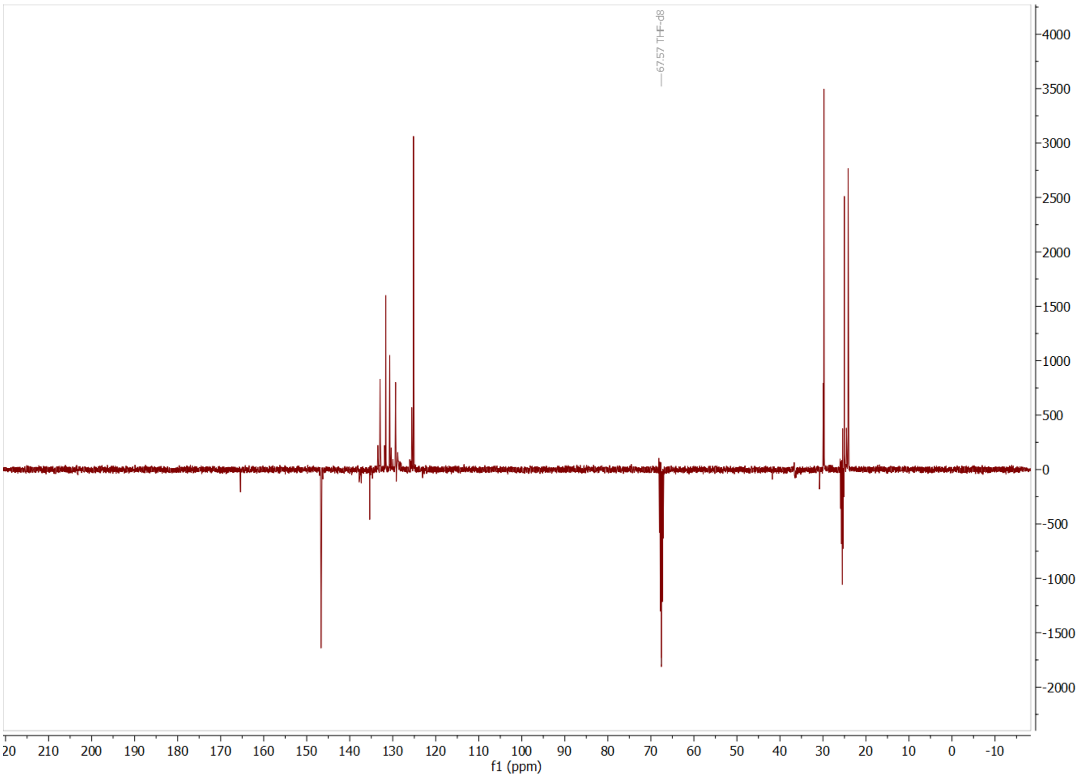

c) ^{31}P NMR spectrum in THF-d_8

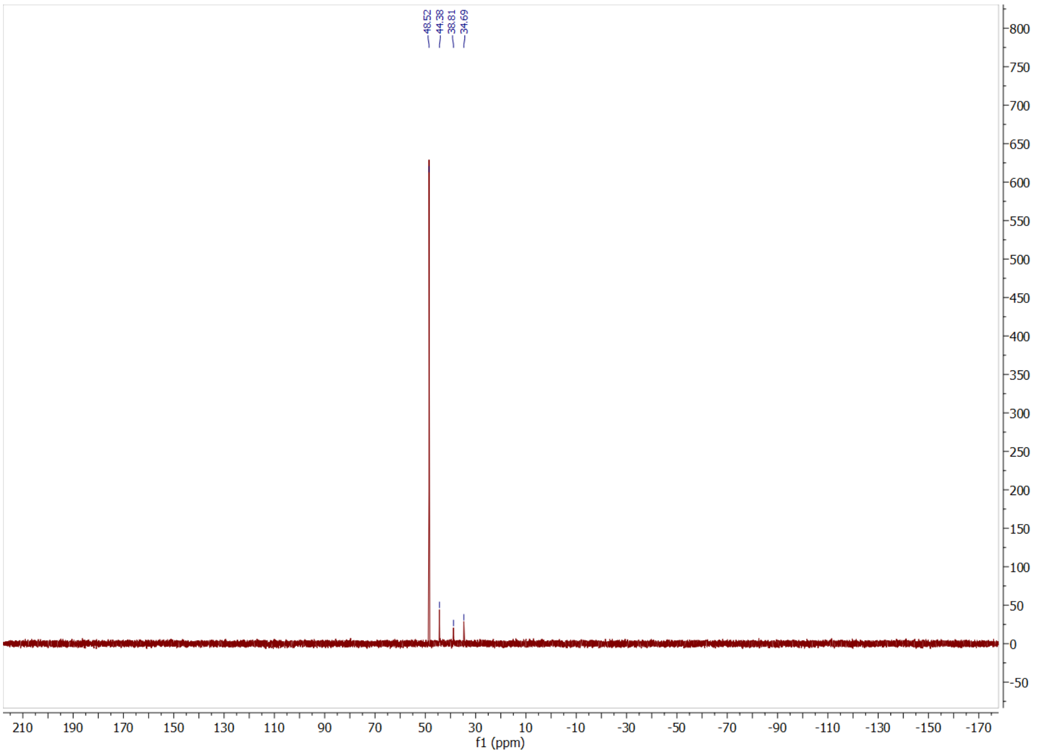

d) $^{1}H,^{31}P$ HMBC NMR spectrum in THF-d_8

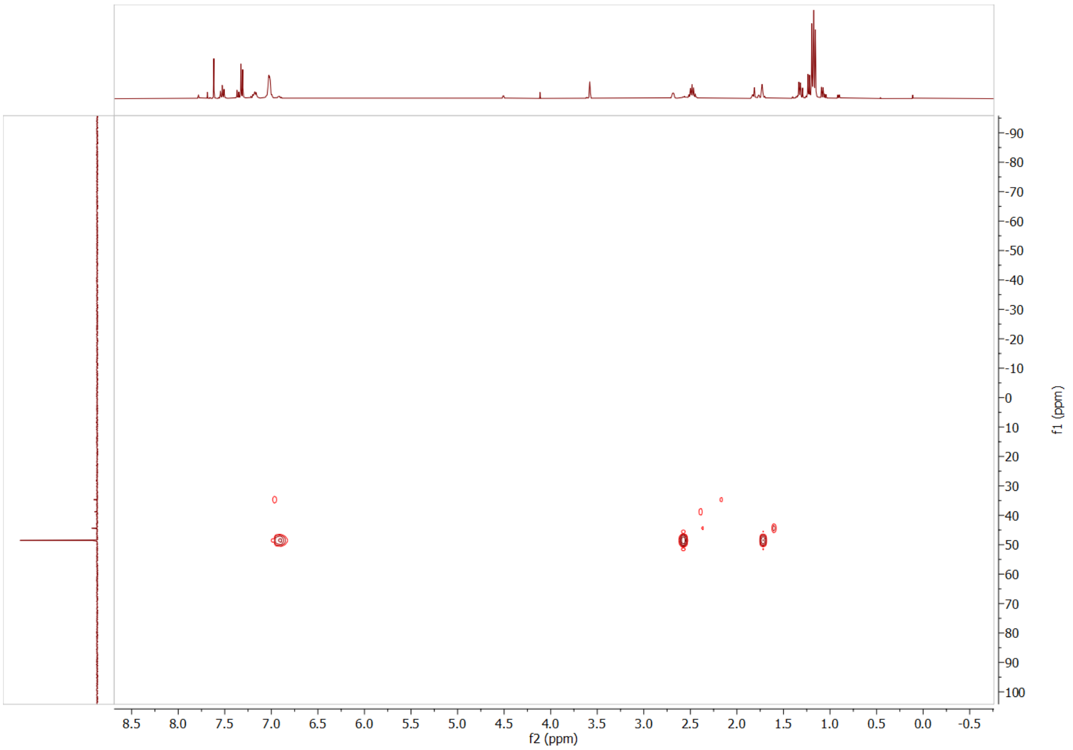

Ru-1 (triphos-Ru-(2-fluorophenolate)$_2$)

a) ^{1}H NMR spectrum in toluene-d_8

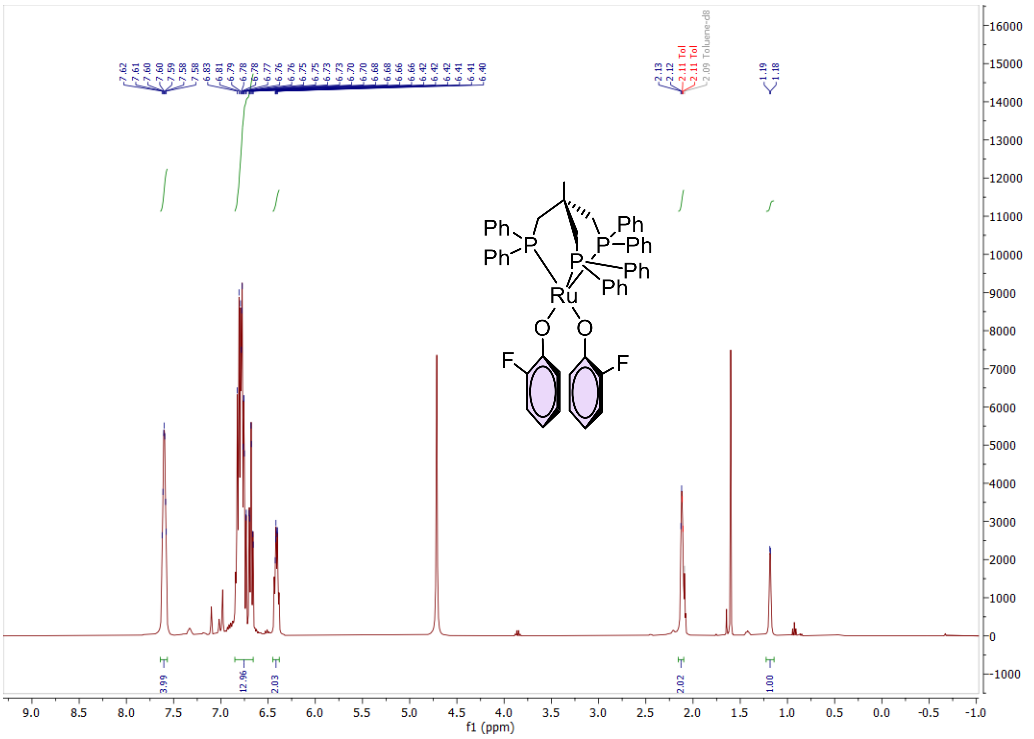

b) ^{13}C APT NMR spectrum in toluene-d_8

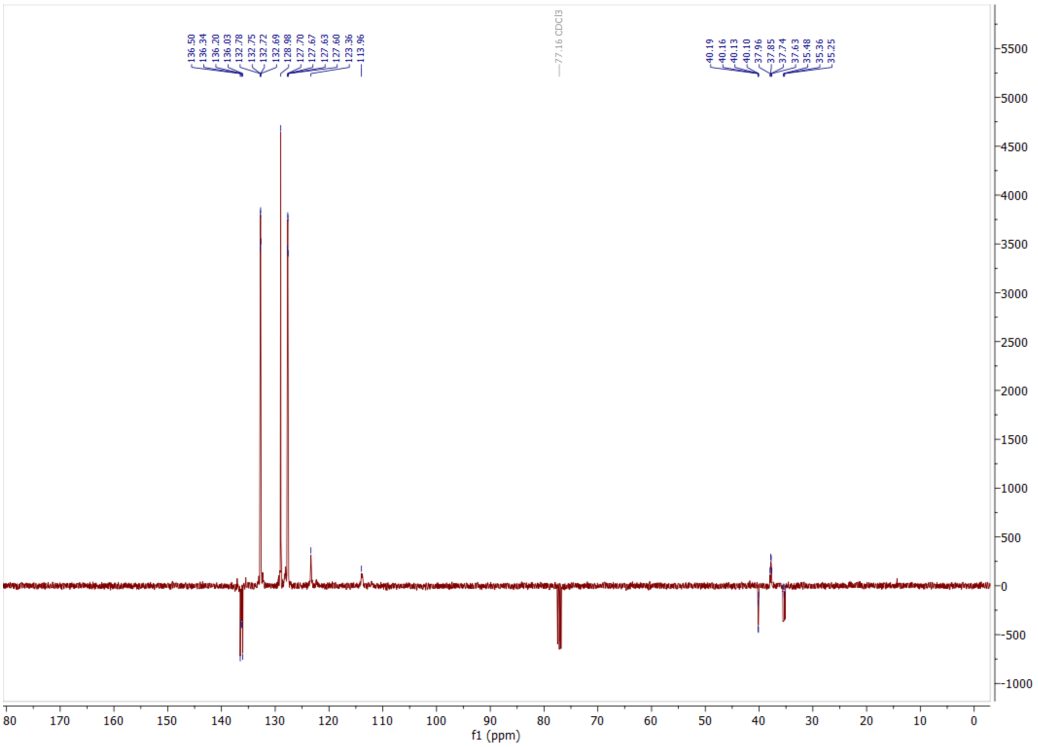

c) ^{19}F NMR spectrum in toluene-d_8

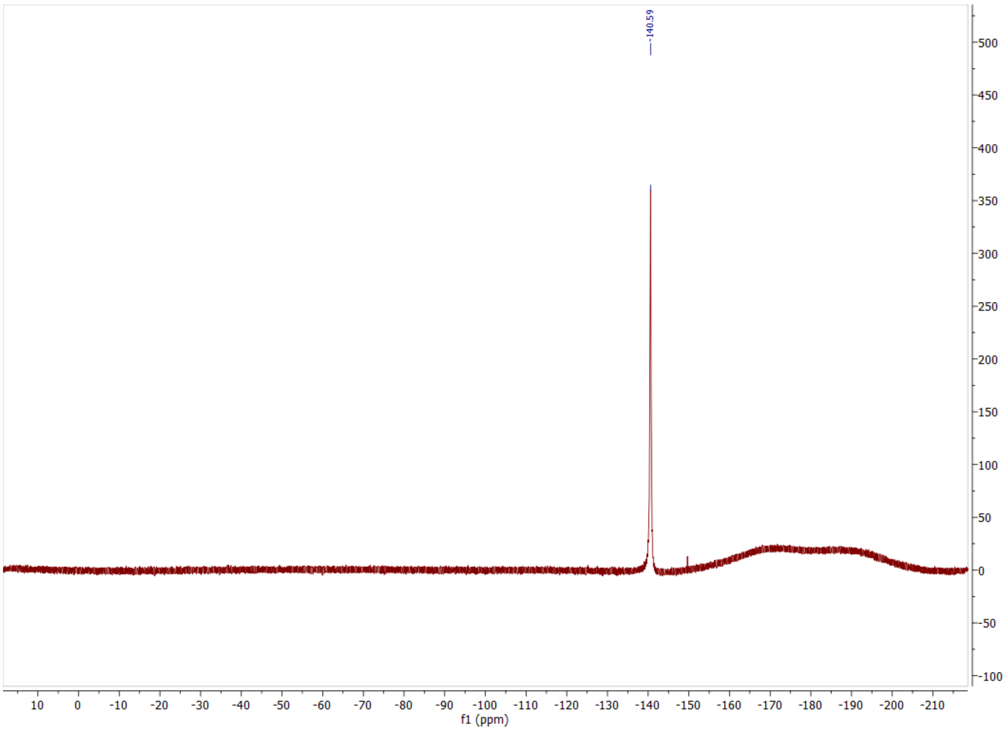

d) ^{13}P NMR spectrum in toluene-d_8

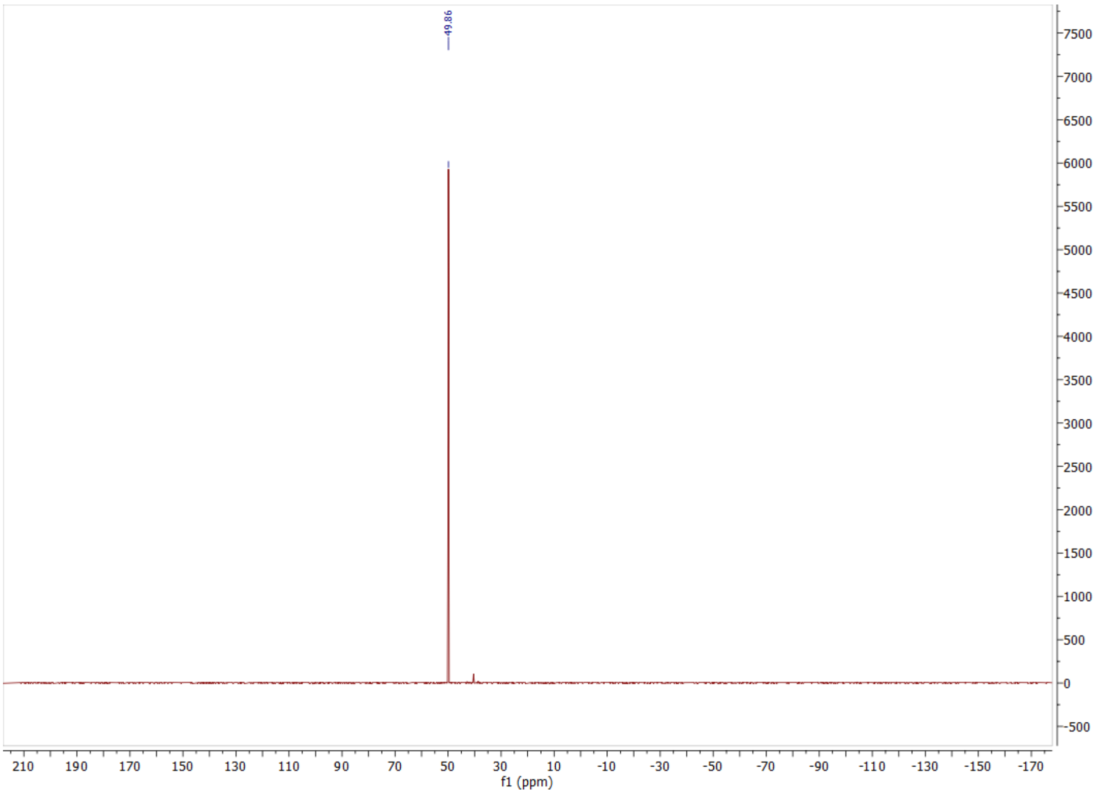

Ru-2 (triphos-Ru-(2,6-difluorophenolate)$_2$)

a) ^{1}H NMR spectrum in toluene-d_8

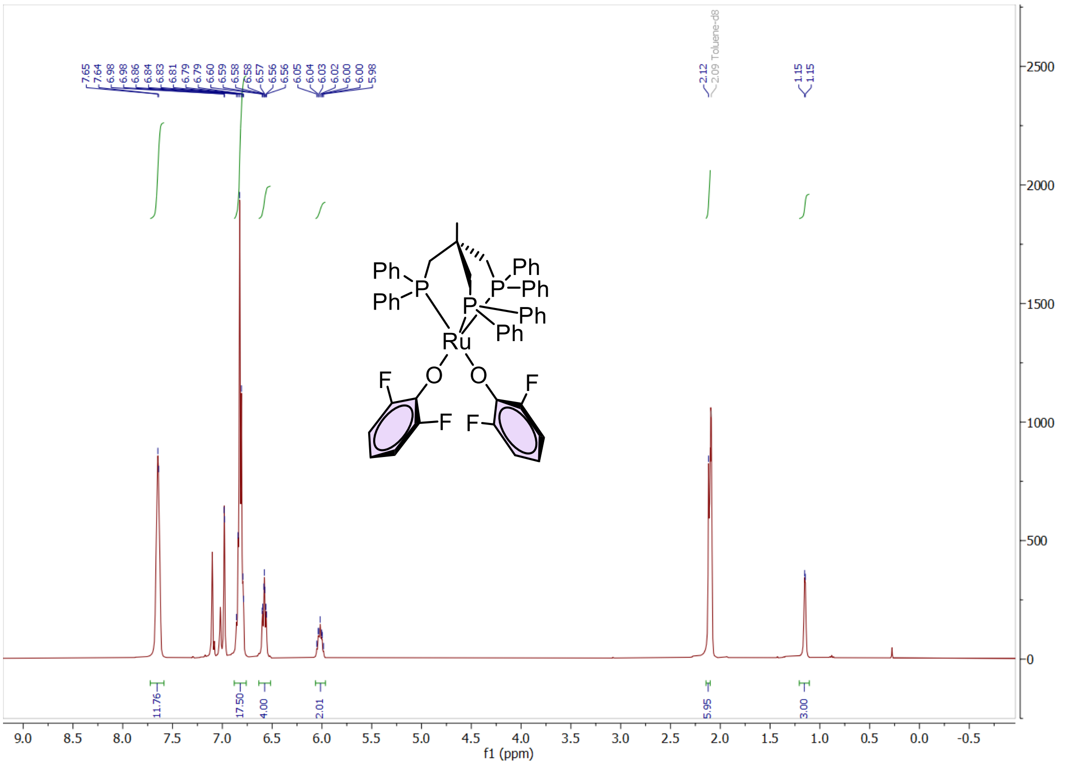

b) ^{13}C APT NMR spectrum in toluene-d_8

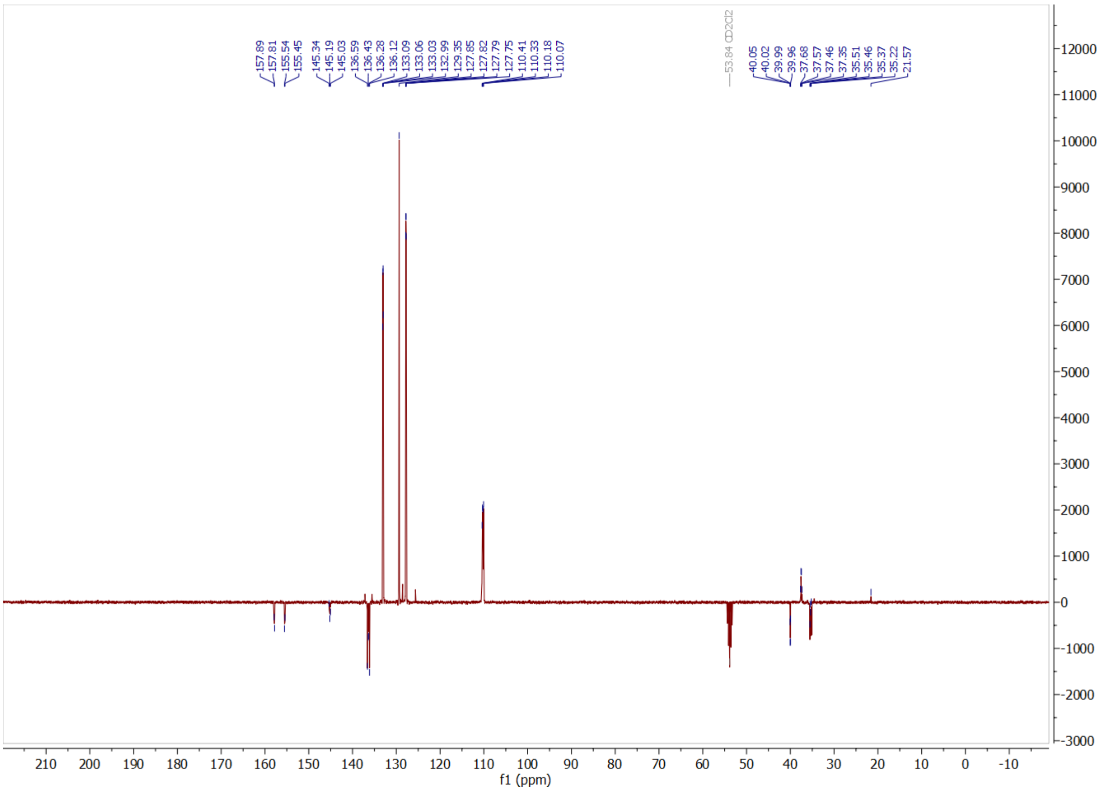

c) ^{31}P NMR spectrum in toluene-d_8

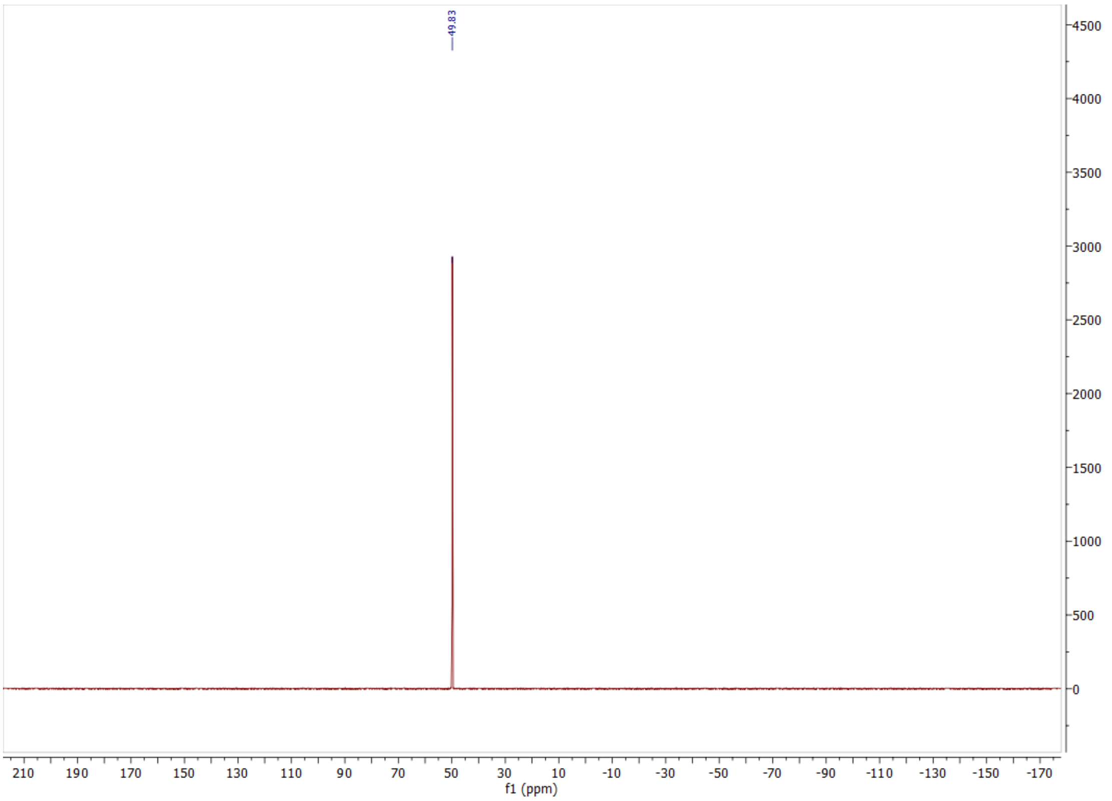

cd) ^{19}F NMR spectrum in toluene-d_8

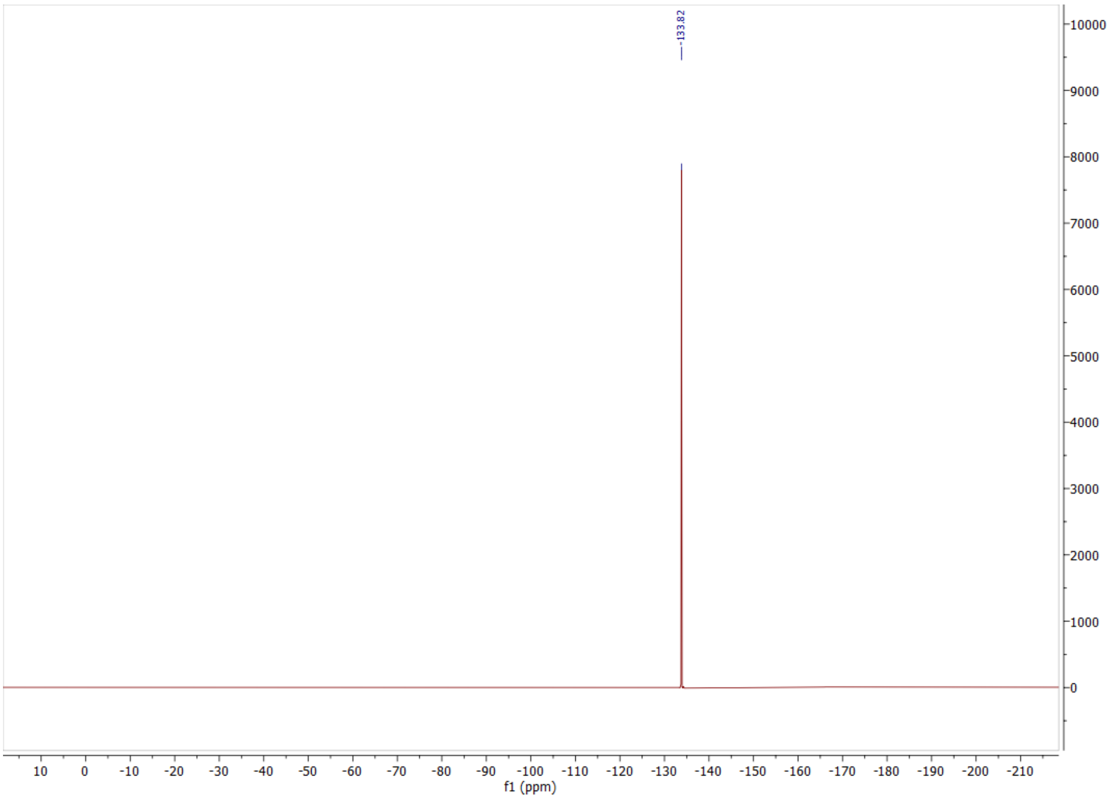

Model 2 (1-(4-(2-(4-methoxyphenyl)propan-2-yl)phenoxy)-3-(phenethylamino)propan-2-ol)

a) ^{1}H NMR spectrum in CD_2Cl_2

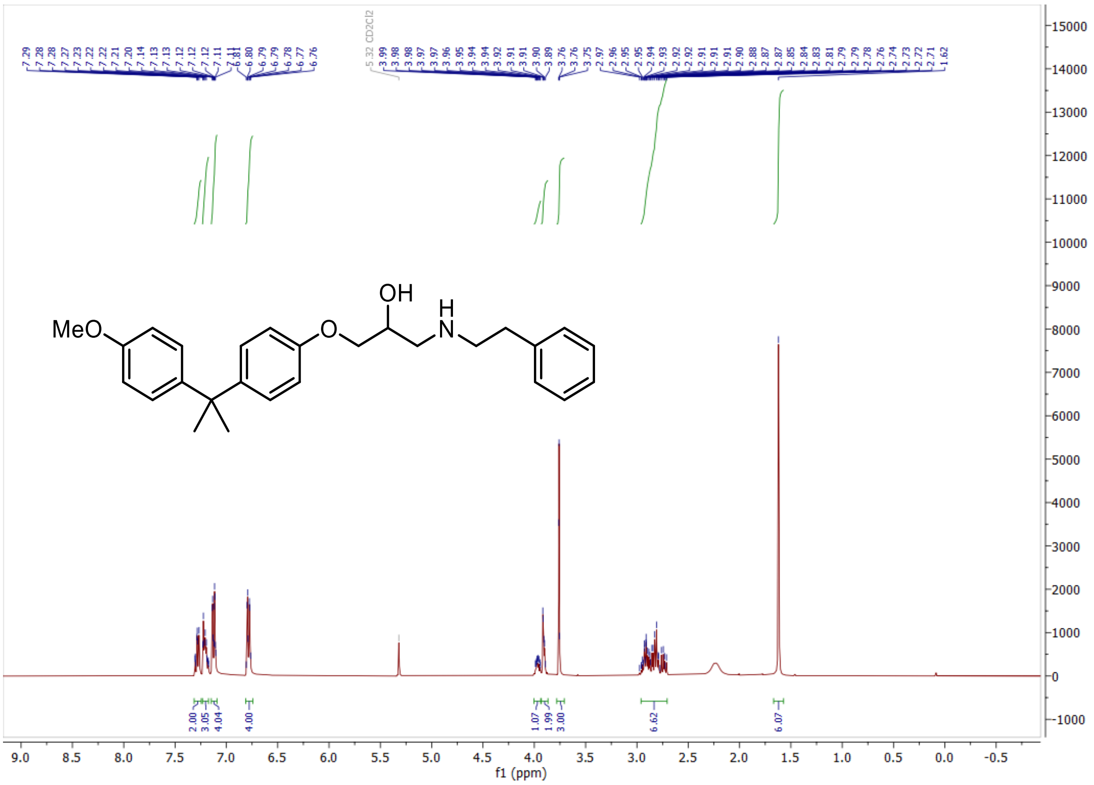

b) ^{13}C NMR spectrum in CD_2Cl_2

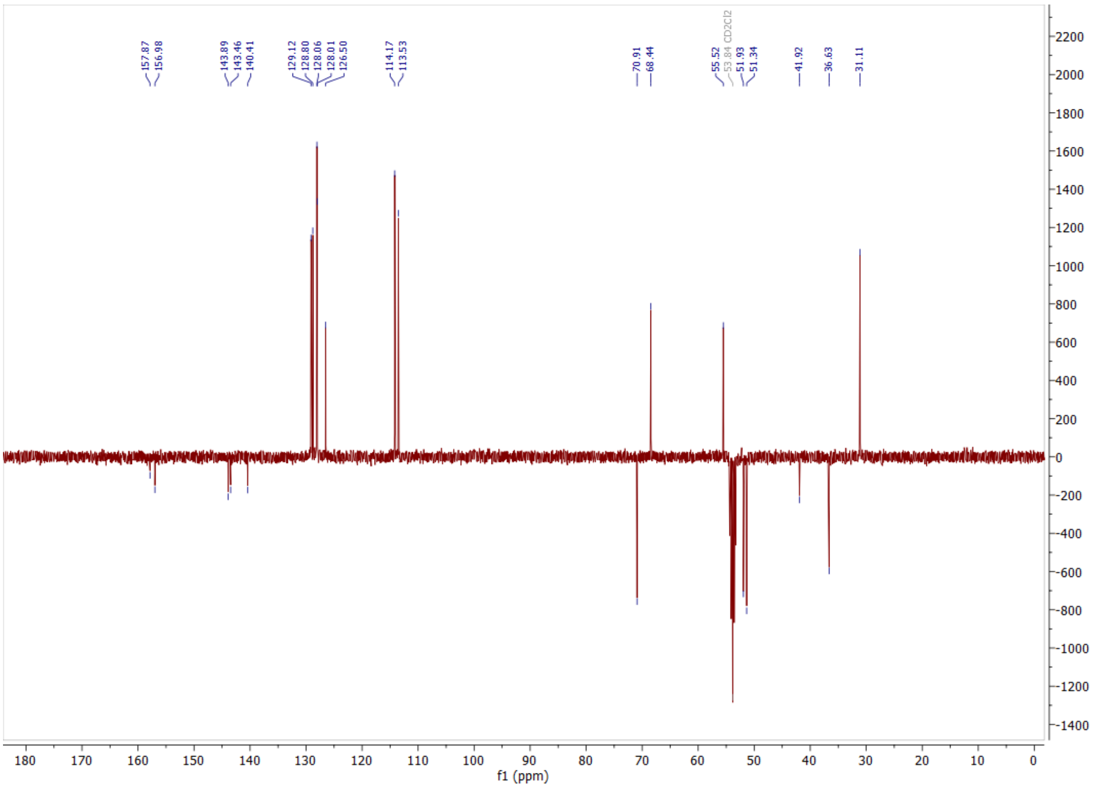

Amine 1 (1-(isopropyl(phenethyl)amino)propan-2-ol) (mixed with amine 2)

a) ^{1}H NMR spectrum in $CDCl_3$

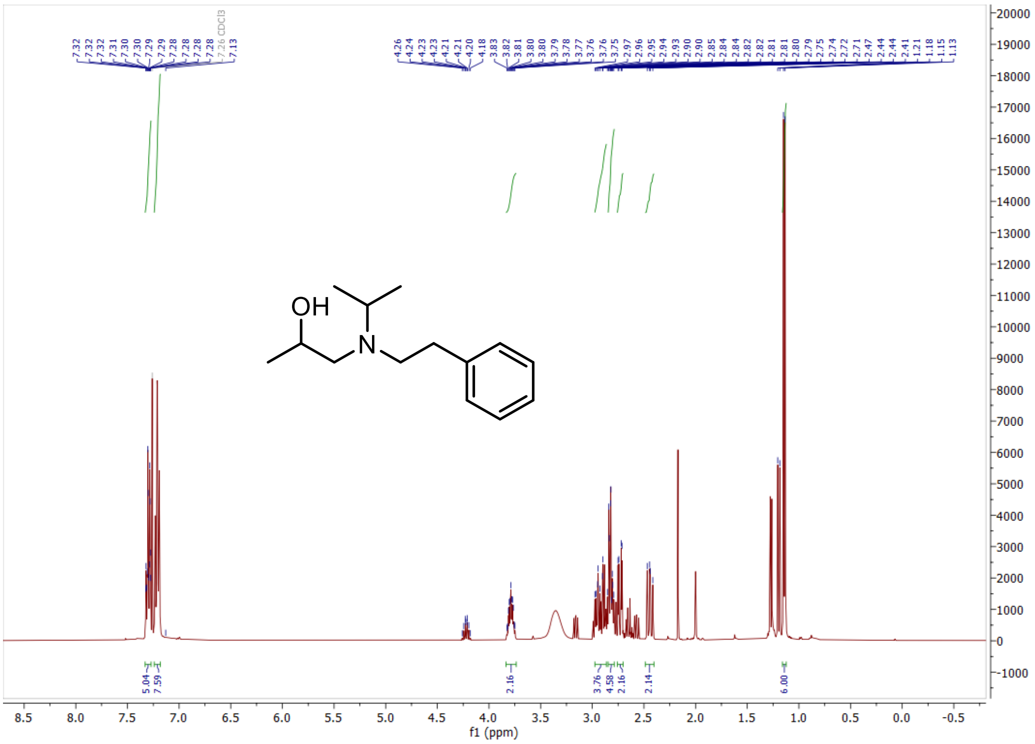

b) ^{13}C APT NMR spectrum in $CDCl_3$

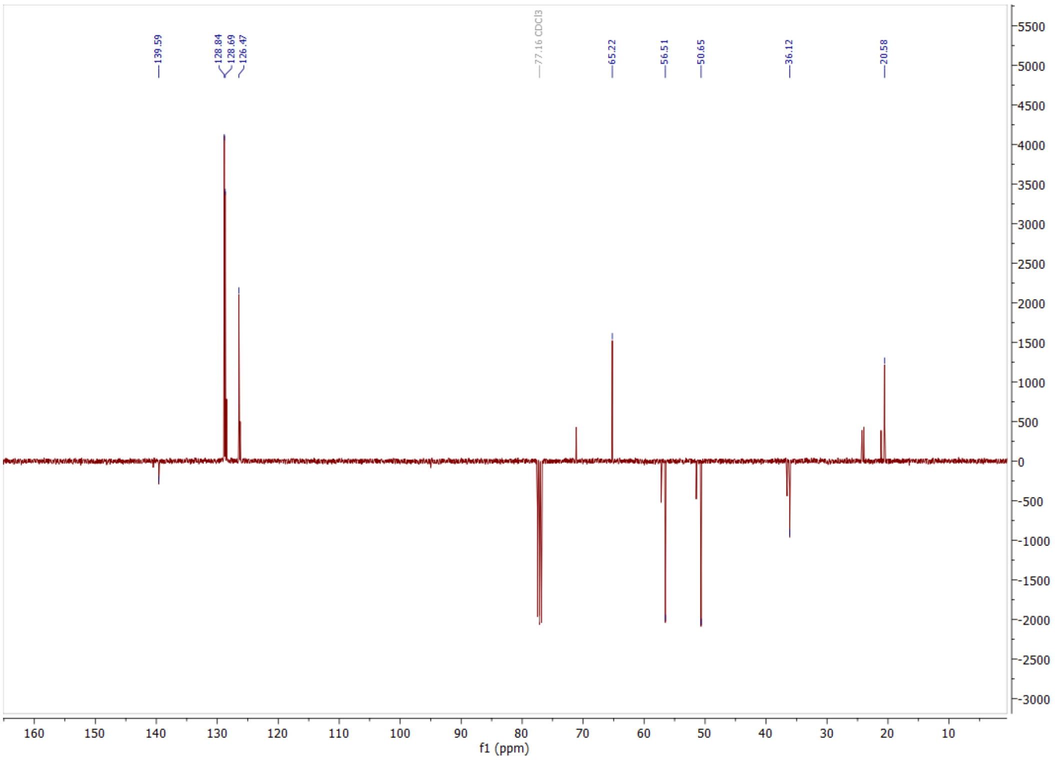

Amine 2 (1-(isopropyl(phenethyl)amino)propan-2-ol) (mixed with amine 1)

a) ^{1}H NMR spectrum in $CDCl_3$

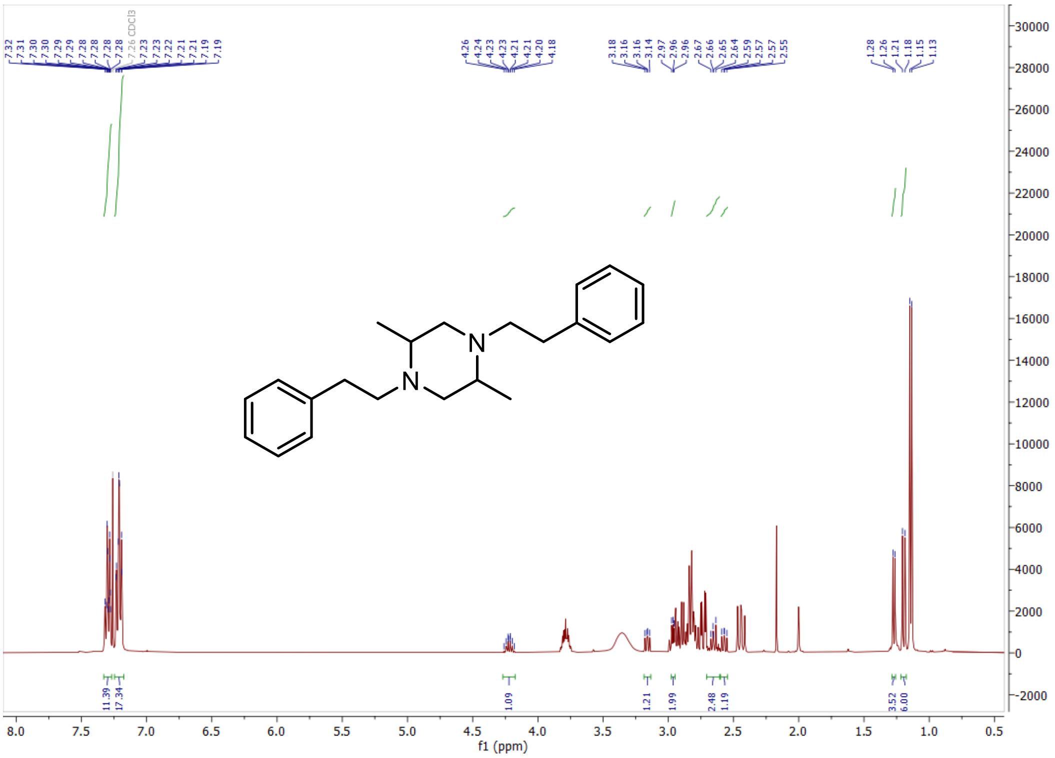

b) ^{13}C APT NMR spectrum in $CDCl_3$

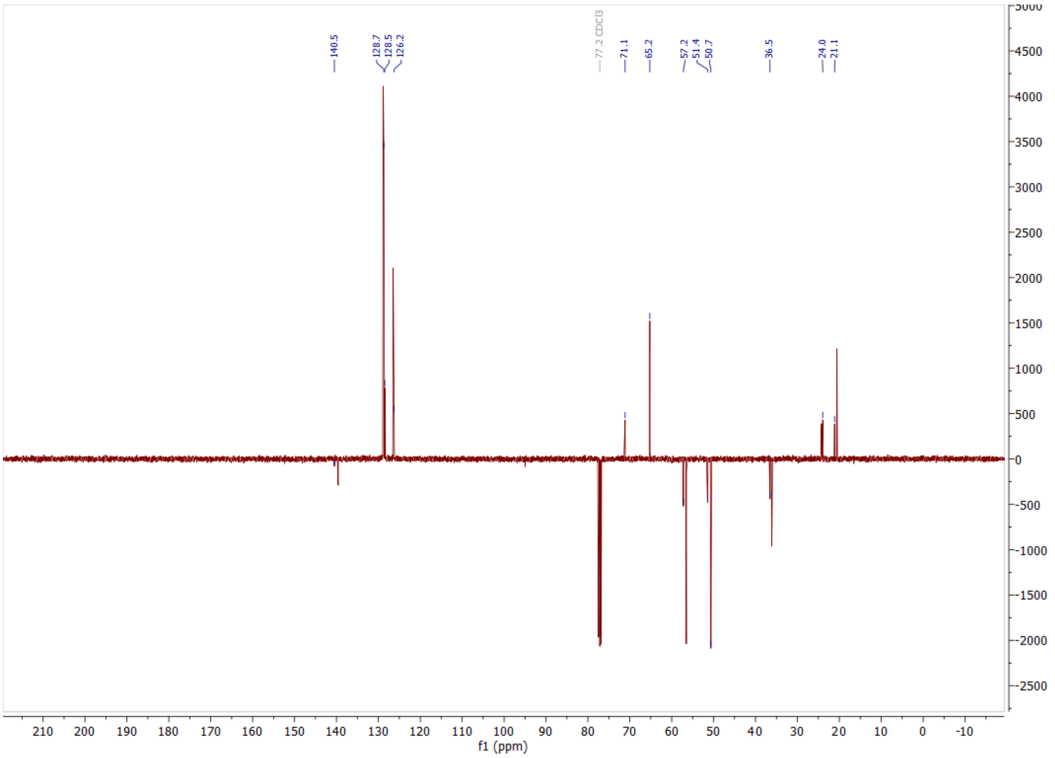

triphos^{xyl}-Ru-Cl_2

a) ^{1}H NMR spectrum in CD_2Cl_2

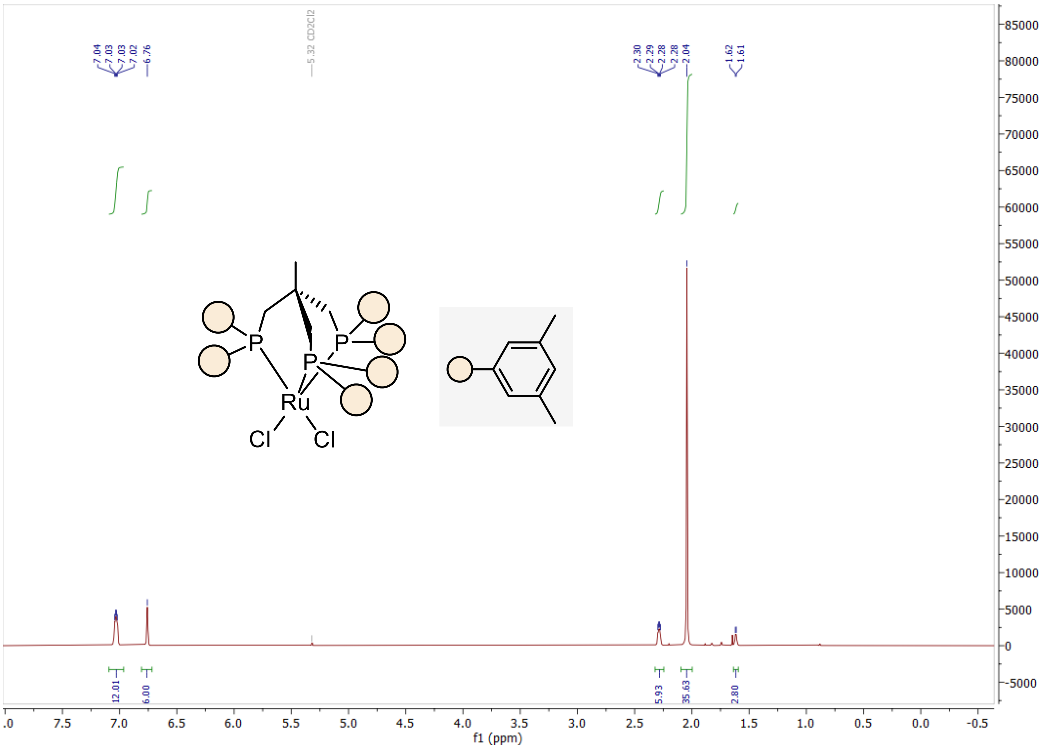

b) ^{13}C APT NMR spectrum in CD_2Cl_2

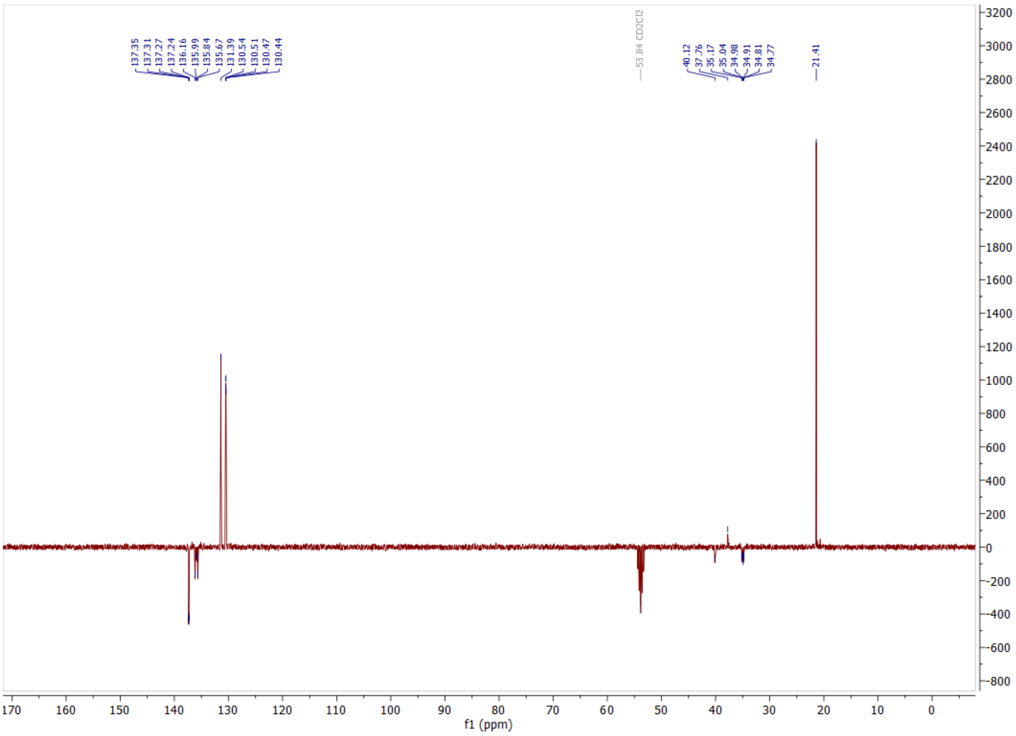

c) ^{31}P NMR spectrum in CD_2Cl_2

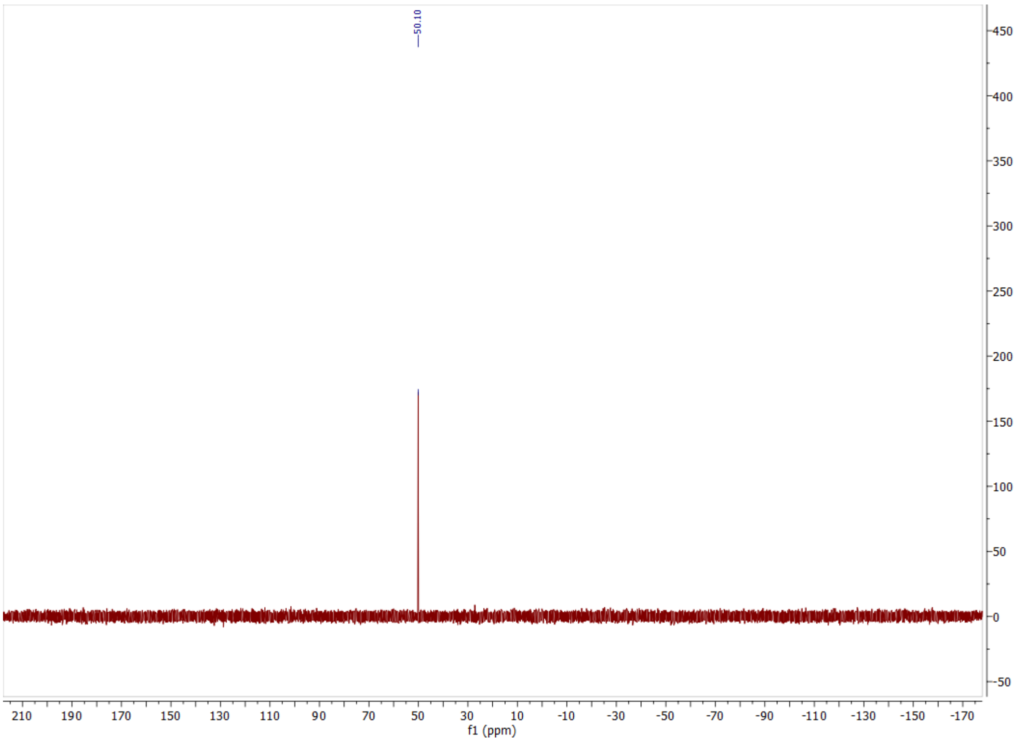

4. Theoretical section

4.1 Computational details

All density functional theory (DFT) results presented in this paper were performed in the Gaussian 16 package, at 433.15 K and 1 atm pressure.[10] The geometry optimisation of all molecules was executed at the M06-D3/def2SVP level of theory with an ultrafine grid and no symmetry constraints.[11-15] The energy refinement was performed with a single-point calculation at the M06-D3/def2TZVP level of theory with the SMD solvation model for toluene. To properly assign all stationary points as minima or saddle points, vibrational analysis was performed at the same level of theory as for geometry optimisation. This analysis was also used for the Gibbs free energy corrections. Besides the vibrational analysis, the transition states were further confirmed through IRC calculations. Conformational analysis of relevant intermediates and transition states was performed with the CREST program and the best conformation was optimised at the described DFT level.[16]

Due to the high computational costs involved with the use of the full triphos-Ru complexes, a compatible model substrate for the epoxy backbone was selected (Supplementary Figure 12).

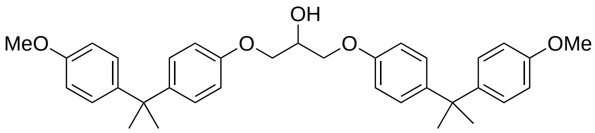

Model 1 - Used in the experimental studies

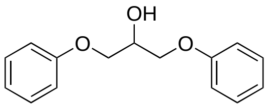

Model S - Used in the computational studies

Supplementary Figure 12 | Details in the computational section; definition of the model compound used for the experimental and computational work.

The energy barrier from I7 to I8 was selected for comparing results using different basis sets (Supplementary Figure 13). Our results show that def2-QZVP for energy refinement returns no changes in the energy barriers. Additionally, using a triple-zeta def2-TZVP basis set for geometry optimisation led to a small change in the barrier (1 kcal/mol), albeit at a much higher computational cost. These results indicate that the accuracy and computational cost for the selected methodology are well balanced.

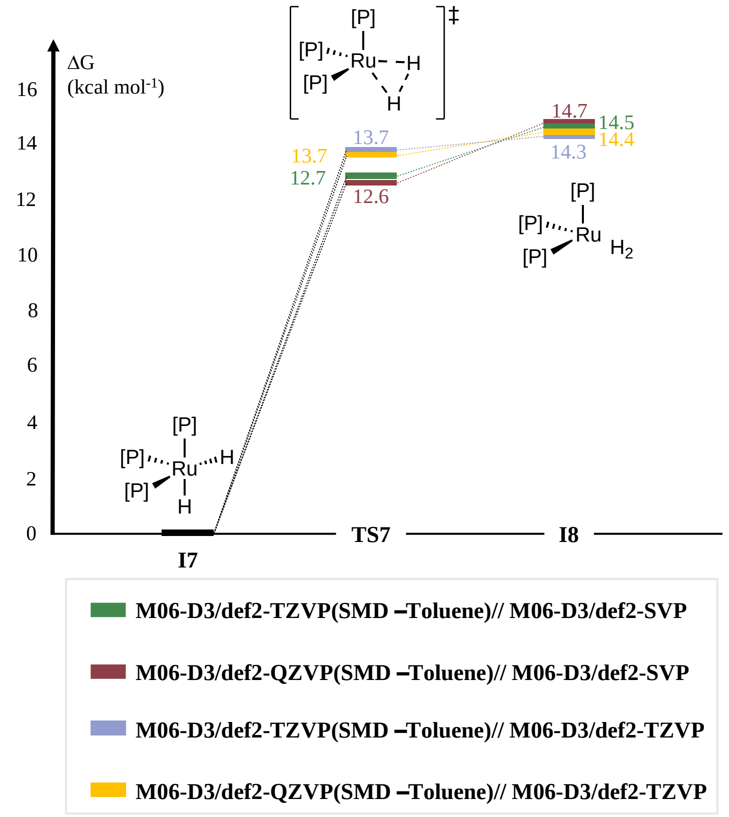

Supplementary Figure 13 | Comparison of the ΔG‡ for **TS7** at different computational levels.

Furthermore, we analyzed the possibility of organic molecules present in the medium to coordinate with intermediates, which could lead to lower energy complexes (Supplementary Figure 14). This analysis was performed using I1, I3, and the coordinating molecules present in the system (iPrOH, PhOH, Model S, Ketone S, Acetone). Despite the coordination ability of these organic molecules, the higher-order coordination complexes were less stable than the separate molecules. Analysis of **I7** with Ketone S or acetone led to the same conclusion. The same was observed in the complex of **I4** with phenol.

1) Example of calculation using I1 and PhOH

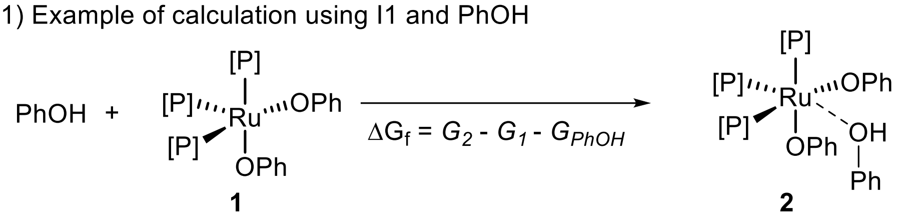

1 **2**

2) Data for different intermediates

**2**	ΔG_f
**I1** + **iPrOH**	**16.2**
**I1** + **PhOH**	**3.5**
**I1** + **Acetone**	**14.9**
**I1** + **ModelS**	**10.4**
**I1** + **KetoneS**	**12.7**
**I3** + **iPrOH**	**2.4**
**I3** + **PhOH**	**3.7**
**I3** + **Acetone**	**3.5**
**I3** + **ModelS**	**2.5**
**I3** + **KetoneS**	**3.0**
**I7** + **KetoneS**	**1.8**
**I7** + **Acetone**	**7.1**
**I4** + **PhOH**	**2.6**

Supplementary Figure 14 | Computed ΔG for the formation complexes **Ru-S** or **I3** with one additional ligand: iPrOH, PhOH, Model S, Ketone S, or Acetone.

4.2 Mechanism of the catalyst activation (energy profiles and discussion)

In the previous publication, an induction period of 2 hours was observed before catalytic cleavage of the epoxy model compounds could be observed.[1] This indicates that catalyst activation is a bottleneck, thus the rate-determining step before an active species enters the catalytic cycle. Additionally, no product is observed in the absence of iPrOH, indicating a critical role of iPrOH in the catalysis. In order to elucidate the role of iPrOH in the activation of the triphos-Ru-TMM pre-catalyst a comparison of the energies for the first protonation in the presence of iPrOH or **Model S** was computed (Supplementary Figure 15). The barrier for iPrOH ($\Delta G^{\ddagger}$ = 32.2 kcal mol^{-1}) is in good agreement with the 2h induction period and significantly lower than the one for **Model S** ($\Delta G^{\ddagger}$ = 38.2 kcal mol^{-1}), giving a good indication of the role of iPrOH in the catalyst activation.

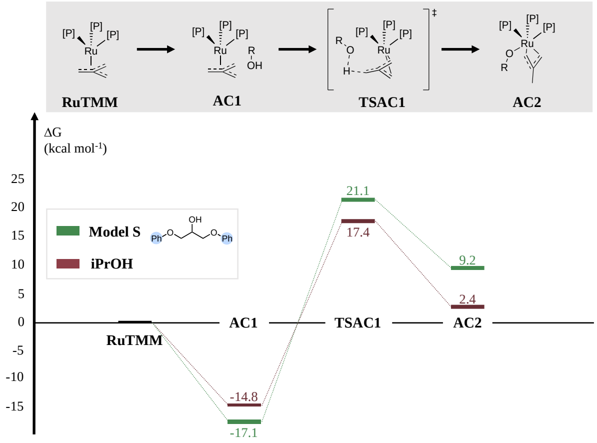

Supplementary Figure 15 | First protonation of the TMM ligand by isopropanol, model S and Benzol.

After the first protonation, **AC2** is obtained, which can lead to **AC5** *via* proton transfer with another isopropanol (Supplementary Figure 16). This step is highly energetic demanding (ΔG‡ = 49.3 kcal mol^{-1}), thus it is unlikely that catalyst activation occurs via this pathway. The reductive elimination of an alkoxy-isobutene species from **AC2** could lead to the formation of Ru(0) species **AC9**, which is capable of entering the catalytic cycle (Supplementary Figure 16). This reductive elimination is another improbable pathway due to the high activation barrier (ΔG‡ = 61.1 kcal mol^{-1}).

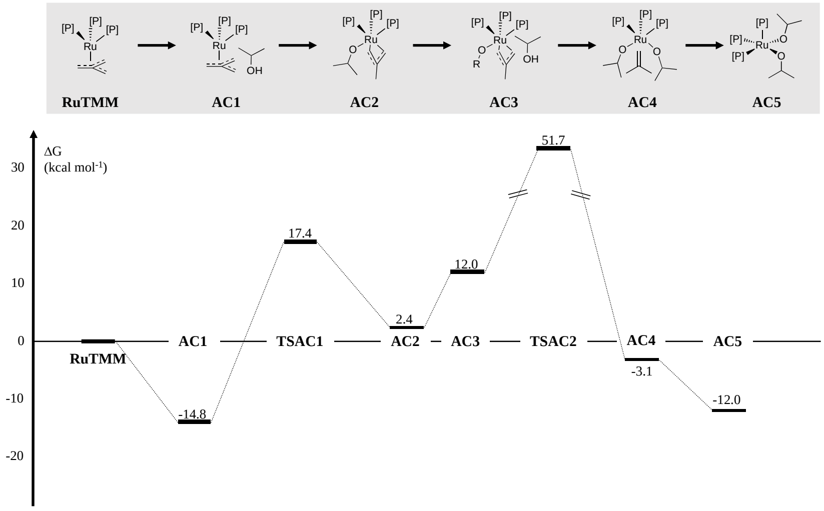

Supplementary Figure 16 | Activation in the presence of isopropanol, consecutive double protonation.

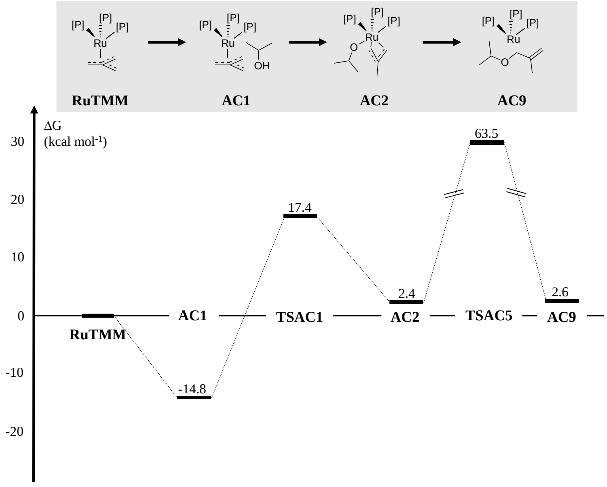

Supplementary Figure 17 | Activation in the presence of isopropanol, elimination of i-Propyl-2-methylallylaether.

The ruthenium complex **AC2** can also undergo beta-hydride elimination for the formation of acetone and a ruthenium-hydride allyl complex **AC7**. From **AC7**, the reductive elimination of isobutene leads to **AC8**, which is able to enter the catalytic cycle as exemplified here by the oxidative addition into a phenol (Supplementary Figure 18).

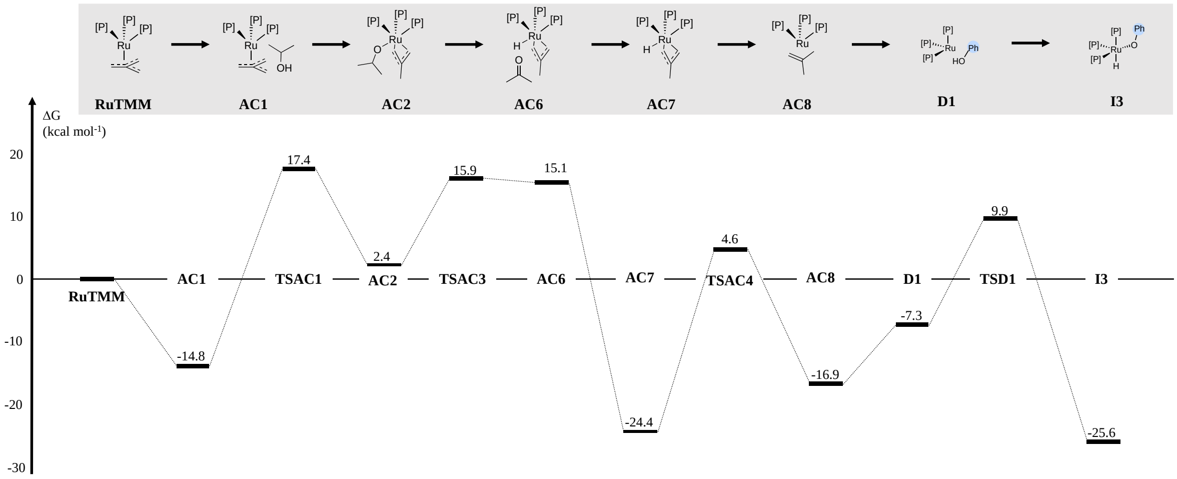

Supplementary Figure 18 | Activation in the presence of isopropanol through a Ru(0).

4.3 Formulation of catalytic cycle (energy profiles and discussion)

Alternative to an oxidative addition, it was considered if a ruthenium hydride complex can perform a hydride attack in an SN_2-like reaction into either **Model S** or **Ketone S**. The starting complex **I1** can undergo ligand exchange with isopropanol leading to **I2** that upon beta-hydride elimination leads to ruthenium hydride complex **I3**. The hydride attack of **I3** into either **Model S** or **Ketone S** (Supplementary Figure 19 and Supplementary Figure 20, respectively) is very energetic demanding, thus unlikely the correct mechanism.

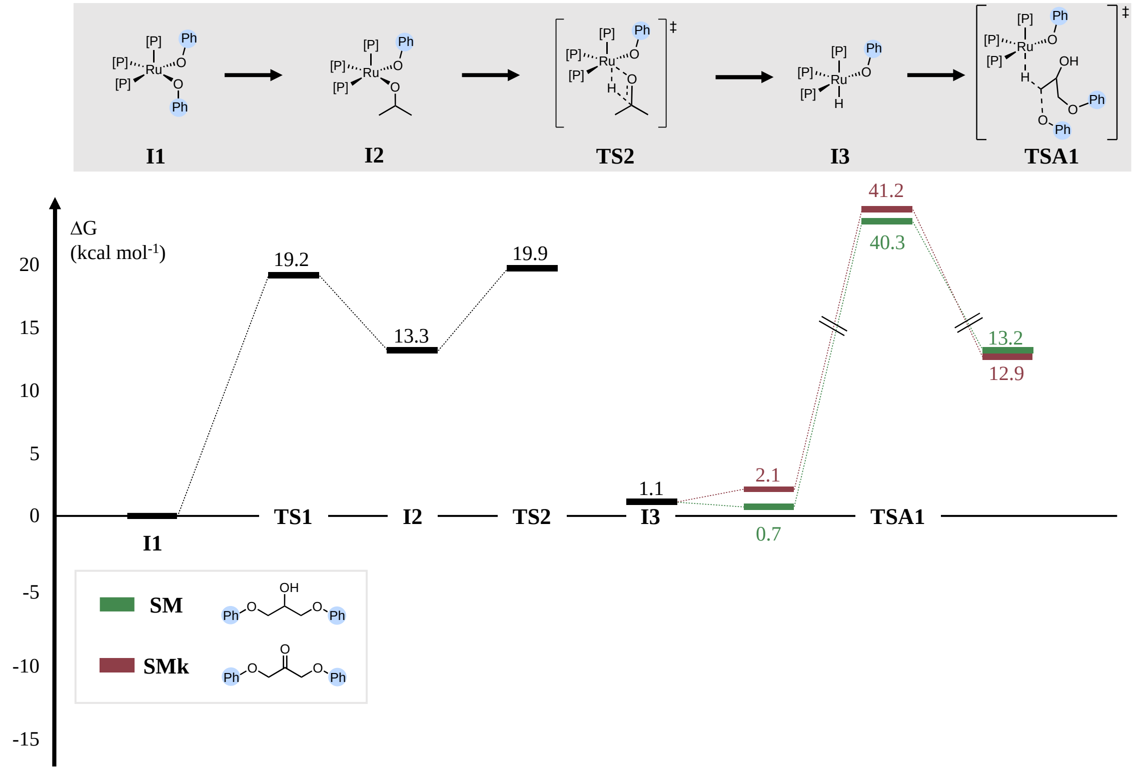

Supplementary Figure 19 | SN2-type C–O bond scission from I3 (mechanism A and B).

Although **I3** is unable to perform the cleavage we decided to further investigate if this was possible with **I4**, which is formed after a second ligand exchange from **I3**. The obtained barrier is in the same range as the ones obtained for **TSA1** and thus also an unlikely mechanism.

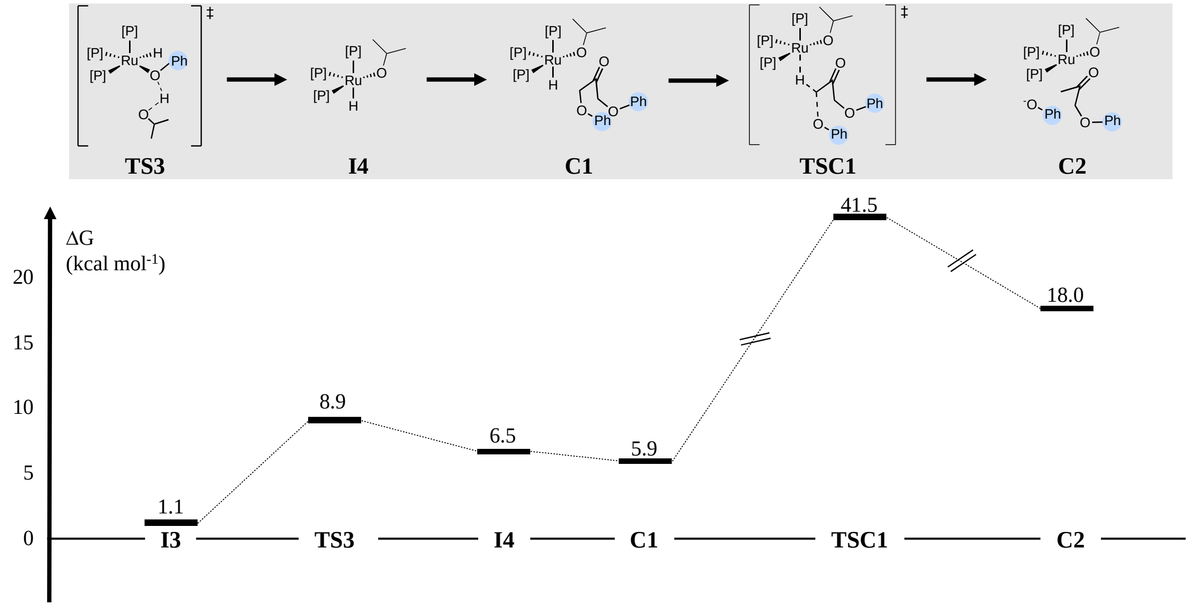

Supplementary Figure 20 | SN2-type C–O bond scission from I4 (mechanism C).

The oxidative addition of a Ru(0) into the C-O bond, as previously proposed, first requires the formation of a ruthenium(0). The reductive elimination of phenol from **I3** was investigated, but unfeasible barriers were obtained (Supplementary Figure 21). Alternatively, oxidisation of an isopropanolate to acetone was considered (Supplementary Figure 22). This pathway is also very energetically demanding and thus implausible to be the correct one.

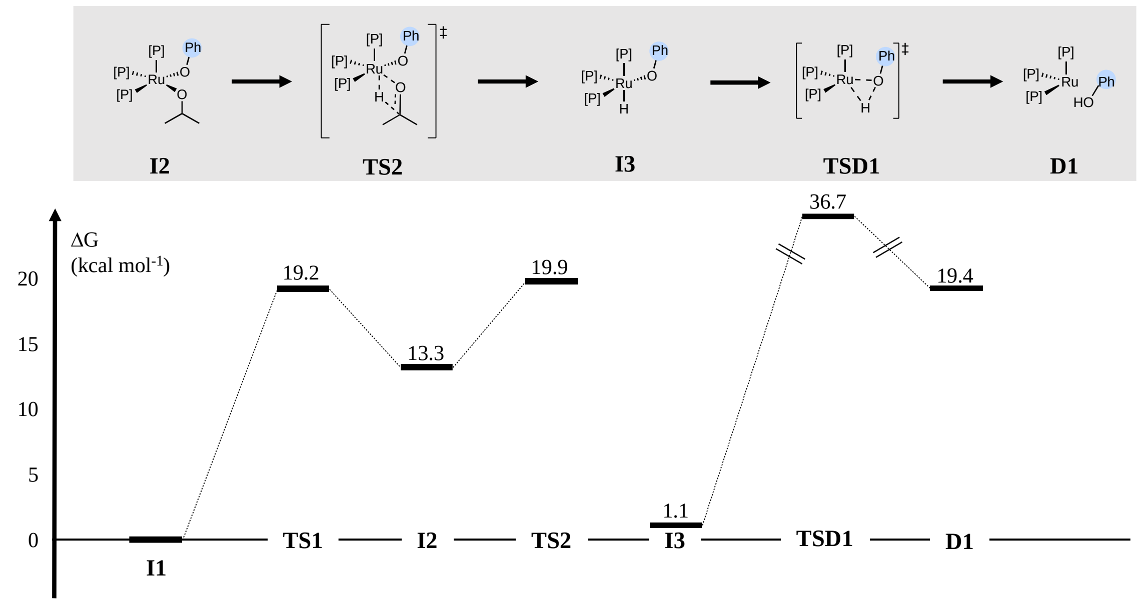

Supplementary Figure 21 | Reductive elimination of phenol from I3 (mechanism D).

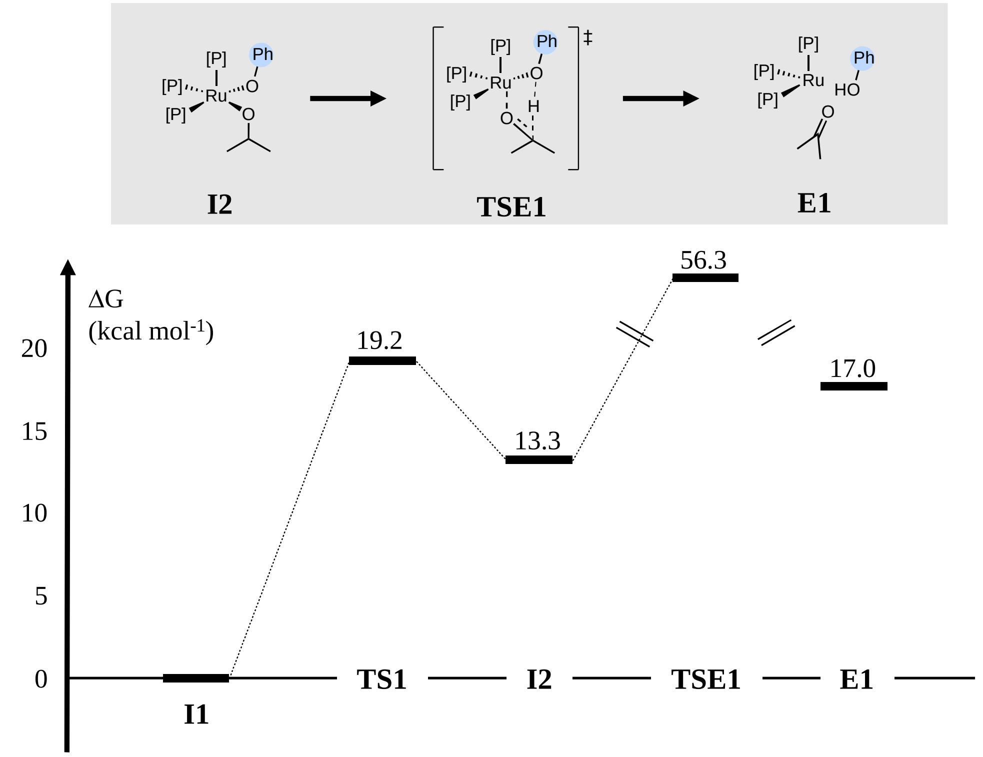

Supplementary Figure 22 | Oxidation of isopropanolate from I2 (mechanism E).

The formation of the ruthenium(0) capable of performing oxidative addition into the C-O bond occurs via the formation of hydrogen gas (Supplementary Figure 23). From ruthenium hydride **I3**, ligand exchange with isopropanol is feasible, leading to **I4**, which can undergo a second oxidisation for the formation of **I7**. The same complex **I7** can be obtained by the reaction of **I3** with **Model S** leading to **I9**, which forms the **Ketone S** and **I7** from an oxidisation reaction.

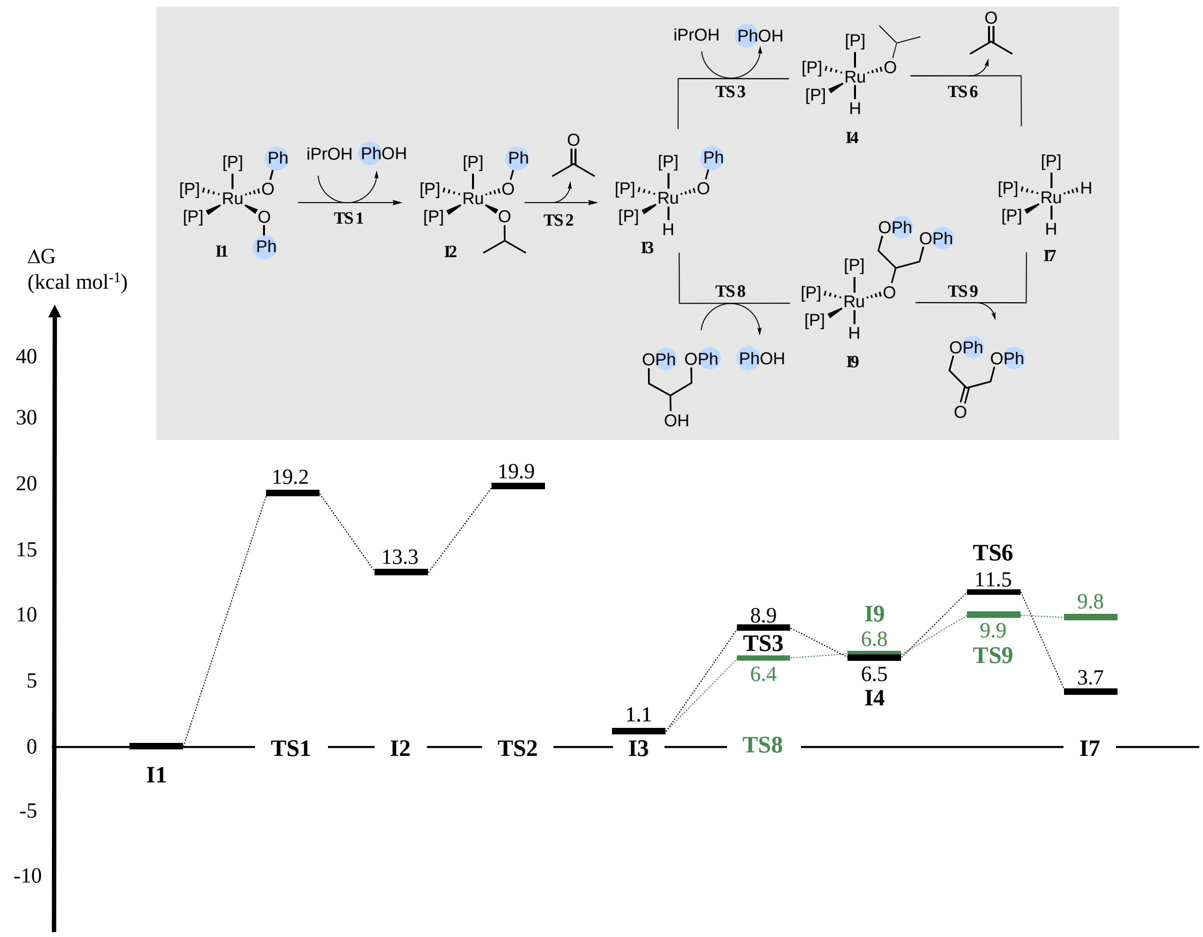

Supplementary Figure 23 | Pathways to the formation of I7.

From **I7** reductive elimination of H_2 is energetically feasible, thus the oxidative addition into both **Model S** and **Ketone S** was computed (Supplementary Figure 24). It is clear that addition into the **Model S** is not feasible at the described reaction conditions ($\Delta G^{\ddagger}$ = 60.1 kcal mol^{-1}), but OA into the C-O bond of **Ketone S** has a barrier of only 25.6 kcal mol^{-1}, leading to **I6**. From **I6**, protonation with a phenol recovers the catalyst.

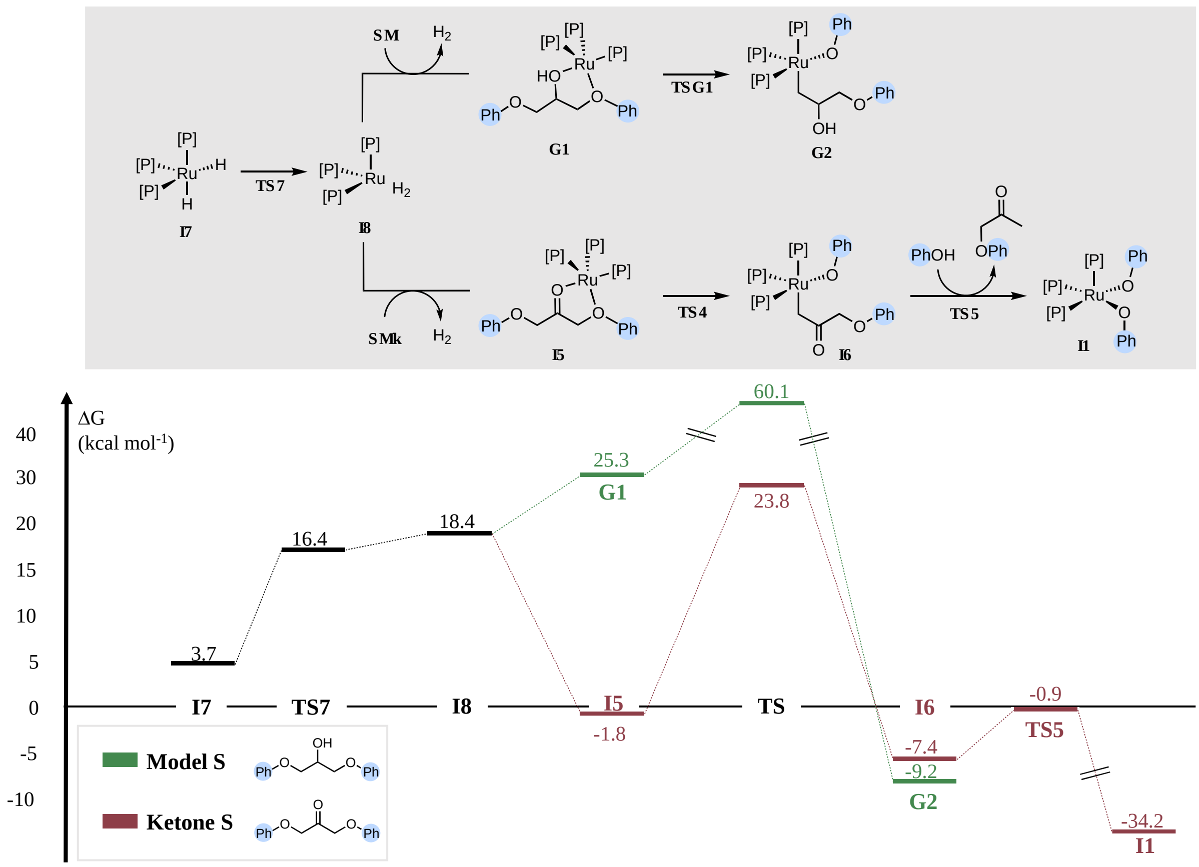

Supplementary Figure 24 Comparison of oxidative addition into Model S and Ketone S.

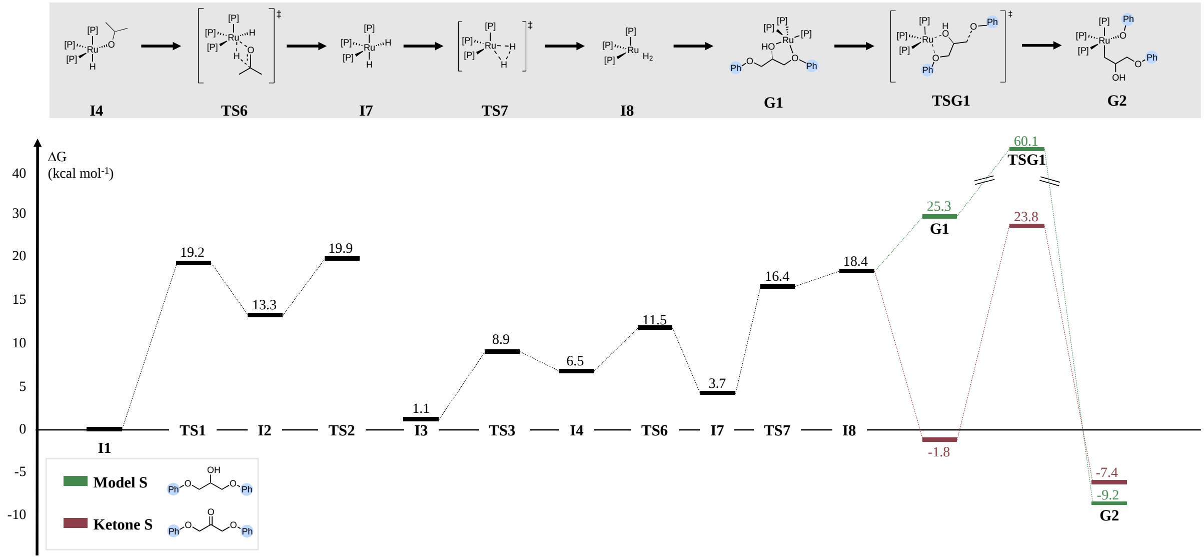

Supplementary Figure 25 | Comparison energy profile mechanisms F and G.

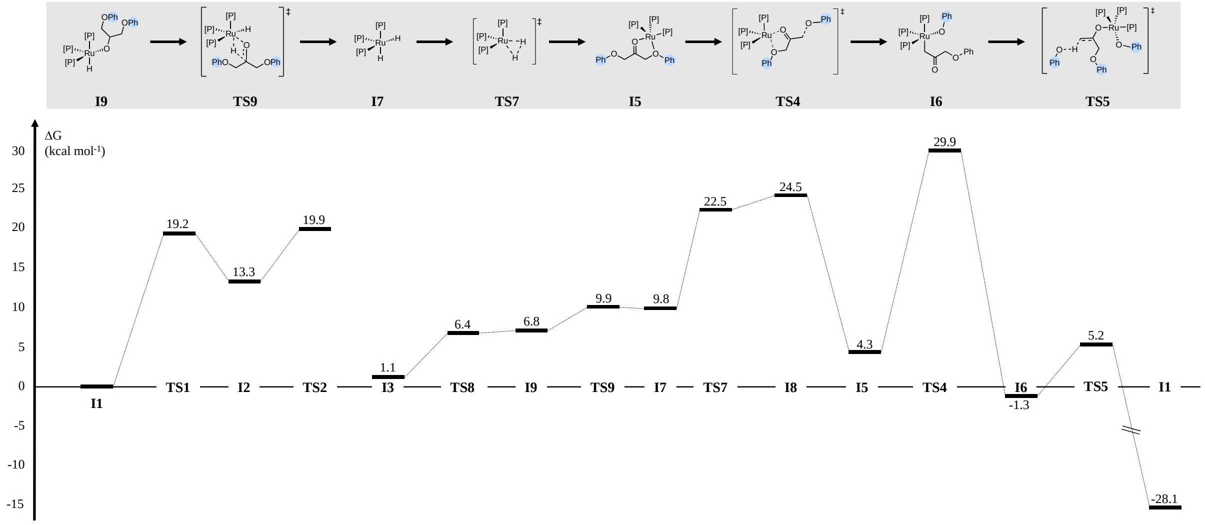

Supplementary Figure 26 | Full energy profile mechanism H.

4.4 Reported data for individual species and cycles

Imaginary frequencies for the transition states:

Transition State	Frequency
TS1	184.8*i*
TS2	290.7*i*
TS3	104.4*i*
TS4	392.3*i*
TS5	1022.3*i*
TS6	133.8*i*
TS7	1021.6*i*
TS7-opt-def2TZVP	1037.3*i*
TS8	426.1*i*
TS9	213.2*i*
TSA1	794.0*i*
TSB1	791.4*i*
TSC1	834.7*i*
TSD1	1287.2*i*
TSE1	426.0*i*
TSG1	638.6*i*
TSAc1	965.6*i*
TSAc2	556.2*i*
TSAc1-ModelS	977.4*i*
TSAc3	51.9*i*
TSAc4	873.0*i*
TSAc5	593.9*i*

Supplementary Table 1: Imaginary frequencies for the transition states

Reported energies:

	G kcal mol^{-1}	H kcal mol^{-1}	E kcal mol^{-1}
Model S	-506038.1671905	-505970.498218	-506166.7620205
Isopropanol	-121885.41457375	-121851.59985375	-121925.13657875
Acetone	-121150.68360875	-121115.97972125	-121174.84424125
Ketone S	-505296.2254975	-505229.040955	-505409.6134925
PhOH	-192841.23031675	-192804.31762925	-192877.85309925
Isobutene	-98573.3375855	-98538.7372355	-98612.726388
TriPhosRuTMM	-1794391.466486	-1794269.181796	-1794799.9319635
AC1	-1916289.7659565	-1916122.4644165	-1916738.9467715
AC2	-1916272.5503685	-1916107.758201	-1916723.5019885
AC1-ModelS	-2300444.805748	-2300251.1686605	-2300989.917508
AC2-ModelS	-2300418.544873	-2300226.7388305	-2300965.556703
AC3	-2038146.4379555	-2037965.276823	-2038657.7211905
AC4	-2038161.594716	-2037978.8447535	-2038670.477136
AC5	-1939598.755833	-1939433.479863	-1940049.278243
AC6	-1916259.91735025	-1916096.56968775	-1916711.91501275
AC7	-1795150.64390975	-1795001.09057975	-1795554.16782475
AC8	-1795143.143716	-1794992.0310485	-1795545.8437235

AC9	-1916272.42580975	-1916107.75098475	-1916723.49414475
TSAc1	-1916257.60325575	-1916097.32532325	-1916710.33948575
TSAc2	-2038106.77198625	-2037929.76804125	-2038617.90524875
TSAc1-ModelS	-2300406.60398725	-2300216.25939225	-2300951.38442725
TSAc3	-1916259.07737875	-1916096.73434375	-1916711.69626625
TSAc4	-1795121.5779215	-1794971.7409615	-1795522.926294
TSAc5	-1916211.46732225	-1916047.34595225	-1916661.14699975
I1	-2081524.29466425	-2081351.71898675	-2081965.89465175
I2	-2010555.15019375	-2010389.34900625	-2011004.57698875
I3	-1889419.6865975	-1889264.6526825	-1889816.8363675
I4	-1818458.5012115	-1818306.4065065	-1818858.526814
I5	-2201875.82646375	-2201694.52539875	-2202356.01832875
I6	-2201881.405629	-2201701.8684665	-2202363.3501015
I7	-1697313.61833825	-1697174.89001075	-1697664.26973075
I8	-1697298.91162075	-1697158.77643825	-1697649.19592575
I9	-2202610.89703175	-2202427.26417675	-2203101.25502425
A1	-2395456.4154325	-2395258.275405	-2396008.514405
A2	-2395443.83537525	-2395242.15624775	-2395993.47329275
B1	-2394713.05916625	-2394510.98157625	-2395245.60335625
B2	-2394702.2495375	-2394499.72454	-2395234.71027
C1	-2323753.3827295	-2323555.461072	-2324290.7109795
C2	-2323741.321552	-2323541.3329095	-2324277.642037
D1	-1889401.37206875	-1889249.54844375	-1889803.00971875
E1	-2010551.48320925	-2010382.57338675	-2010997.63445425
G1	-2202590.6717655	-2202406.6360555	-2203083.4224155
G2	-2202625.15904	-2202443.3465625	-2203119.838625
TS1	-2203388.5839485	-2203203.970311	-2203892.154581
TS2	-2010548.573429	-2010381.8724065	-2010993.7627165
TS3	-2011295.4207915	-2011127.3398965	-2011753.722319
TS4	-2201850.1808525	-2201670.4886975	-2202330.30683
TS5	-2394714.1873485	-2394514.129681	-2395246.609176
TS6	-1818453.43220375	-1818301.39460125	-1818851.06703125
TS7	-1697300.894709	-1697159.9638565	-1697647.531984
TS8	-2395450.689997	-2395253.7836345	-2396001.9280395
TS9	-2202607.89275	-2202422.3591975	-2203093.77604
TSA1	-2395416.7697315	-2395216.383254	-2395964.5257765
TSB1	-2394673.9015975	-2394475.720155	-2395208.800795
TSC1	-2323717.825944	-2323519.6156365	-2324252.837464
TSD1	-1889384.15095875	-1889227.14481125	-1889776.31273125
TSE1	-2010512.12778975	-2010343.64654975	-2010955.27644725
TSG1	-2202555.837107	-2202373.037572	-2203047.3509545
I1+iPrOH	-2203391.5643225	-2203207.0115525	-2203898.1959
I1+PhOH	-2274360.1529535	-2274171.3896585	-2274861.319006
I1+Acetone	-2202657.038048	-2202470.4427655	-2203145.659748
I1+ModelS	-2587550.16059575	-2587333.37566075	-2588145.87583825

I1+KetoneS	-2586805.96655425	-2586592.12835925	-2587389.53276925
I3+iPrOH	-2011300.753914	-2011130.9806515	-2011760.055049
I3+PhOH	-2082255.36153125	-2082082.80844375	-2082710.50547875
I3+Acetone	-2010563.80605425	-2010392.33226675	-2011005.44180925
I3+ModelS	-2395453.4965535	-2395255.028971	-2396006.066151
I3+KetoneS	-2394711.02794875	-2394509.34003625	-2395244.26615375
I4+PhOH	-2011295.266552	-2011125.6489095	-2011753.770762
I7+KetoneS	-2202606.14723325	-2202421.29640075	-2203094.15021825
I7+Acetone	-1818454.133435	-1818300.6827025	-1818851.396155
I7-opt-def2TZVP	-1697315.9237105	-1697303.575138	-1697793.926228
TS7-opt-def2TZVP	-1697428.2698045	-1697288.690587	-1697777.2162795
I8-opt-def2TZVP	-1697427.5956185	-1697287.5602085	-1697778.855686

Supplementary Table 2: G, H and E for reported structures.

	DG kcal mol^{-1}	DH kcal mol^{-1}	DE kcal mol^{-1}
TriPhosRuTMM + 2 x iPrOH	0	0	0
AC1	-14.8	-1.7	-13.9
TSAc1	17.4	23.5	14.7
AC2	2.4	13.0	1.6
AC3	12.1	7.1	-7.5
TSAc2	51.7	42.6	32.3
AC4	-3.1	-6.5	-20.3
AC5	-11.7	0.2	-11.8

Supplementary Table 3: Gibbs free-energy and enthalpy for activation 1.

	DG kcal mol^{-1}	DH kcal mol^{-1}	DE kcal mol^{-1}
TriPhosRuTMM + Model S	0	0	0
AC1-ModelS	-17.1	-11.5	-23.2
TSAc1-ModelS	21.1	23.4	15.3
AC2-ModelS	9.2	12.9	1.1

Supplementary Table 3: Gibbs free-energy and enthalpy for activation 2.

	DG kcal mol^{-1}	DH kcal mol^{-1}	DE kcal mol^{-1}
TriPhosRuTMM + iPrOH	0	0	0
AC1	-14.8	-1.7	-13.9
TSAc1	17.4	23.5	14.7
AC2	2.4	13.0	1.6
TSAc5	63.5	73.4	63.9
AC9	2.6	13.0	1.6

Supplementary Table 3: Gibbs free-energy and enthalpy for activation 3.

	DG kcal mol^{-1}	DH kcal mol^{-1}	DE kcal mol^{-1}
TriPhosRuTMM + iPrOH + PhOH	0	0	0

AC1	-14.8	-1.7	-13.9
TSAc1	17.4	23.5	14.7
AC2	2.4	13.0	1.6
TSAc3	15.9	24.0	12.5
AC6	15.1	24.2	13.2
AC7	-24.4	3.7	-3.9
TSAc4	4.6	33.1	27.3
AC8	-16.9	12.8	4.4
D1	-7.3	20.8	12.3
TSD1	9.9	43.2	39.0
I3	-25.6	5.7	-1.5

Supplementary Table 4: Gibbs free energy and enthalpy for activation 4.

	DG kcal mol^{-1}	DH kcal mol^{-1}	DE kcal mol^{-1}
I1 + IprOH + Model S	0	0	0
TS1 + Model S	19.2	-0.7	-1.1
I2 + Model S + PhOH	13.3	9.6	8.6
TS2 + Model S + PhOH	19.9	17.1	19.4
I3 + Model S + Acetone + PhOH	1.1	17.5	21.5
A1 + Acetone + PhOH	0.7	-5.7	-3.4
TSA1 + Acetone + PhOH	40.3	36.2	40.6
A2 + Acetone + PhOH	13.2	10.5	11.6

Supplementary Table 5: Gibbs free energy and enthalpy for mechanism A.

	DG kcal mol^{-1}	DH kcal mol^{-1}	DE kcal mol^{-1}
I1 + IprOH + Ketone S	0	0	0
TS1 + Ketone S	19.2	-0.7	-1.1
I2 + Ketone S + PhOH	13.3	9.6	8.6
TS2 + Ketone S + PhOH	19.9	17.1	19.4
I3 + Ketone S + Acetone + PhOH	1.1	17.5	21.5
B1 + Acetone + PhOH	2.1	0.2	2.4
TSB1 + Acetone + PhOH	41.2	35.4	39.2
B2 + Acetone + PhOH	12.9	11.4	13.3

Supplementary Table 5: Gibbs free energy and enthalpy for mechanism B.

	DG kcal mol^{-1}	DH kcal mol^{-1}	DE kcal mol^{-1}
I1 + 2 x IprOH + Ketone S	0	0	0
TS1 + Ketone S	19.2	-0.7	-1.1
I2 + Ketone S + PhOH	13.3	9.6	8.6
TS2 + Ketone S + PhOH	19.9	17.1	19.4
I3 + Ketone S + Acetone + PhOH	1.1	17.5	21.5
TS3 + Ketone S + Acetone + PhOH	8.9	6.3	9.8
I4 + 2 x PhOH + Acetone + Ketone S	6.5	22.9	27.1
C1 + 2 x PhOH + Acetone	5.9	2.9	4.6
TSC1 + 2 x PhOH + Acetone	41.5	38.7	42.4
C2 + 2 x PhOH + Acetone	18.0	17.0	17.6

Supplementary Table 6: Gibbs free energy and enthalpy for mechanism C.

	DG kcal mol^{-1}	DH kcal mol^{-1}	DE kcal mol^{-1}
I1 + IprOH	0	0	0
TS1	19.2	-0.7	-1.1
I2 + PhOH	13.3	9.6	8.6
TS2 + PhOH	19.9	17.1	19.4
I3 + Acetone + PhOH	1.1	17.5	21.5
TSD1 + Acetone + PhOH	36.7	55.0	62.0
D1 + Acetone + PhOH	19.4	32.6	35.3

Supplementary Table 7: Gibbs free energy and enthalpy for mechanism D.

	DG kcal mol^{-1}	DH kcal mol^{-1}	DE kcal mol^{-1}
I1 + IPrOH	0	0	0
TS1	19.2	-0.7	-1.1
I2 + PhOH	13.3	9.6	8.6
TSE1 + PhOH	56.3	55.3	57.9
E1 + PhOH	17.0	16.4	15.6

Supplementary Table 8: Gibbs free energy and enthalpy for mechanism E.

	DG kcal mol^{-1}	DH kcal mol^{-1}	DE kcal mol^{-1}
I1 + 2 x IprOH +Ketone S	0	0	0
TS1 + Ketone S + IPrOH	19.2	-0.7	-1.1
I2 + Ketone S + PhOH + IPrOH	13.3	9.6	8.6
TS2 + Ketone S + PhOH + IPrOH	19.9	17.1	19.4
I3 + Ketone S + Acetone + PhOH + IPrOH	1.1	17.5	21.5
TS3 + Ketone S + Acetone + PhOH	8.9	6.3	9.8
I4 + 2 x PhOH + Acetone + Ketone S	6.5	22.9	27.1
TS6 + 2 x PhOH + Acetone + Ketone S	11.5	27.9	34.6
I7 + 2 x PhOH + 2 x Acetone + Ketone S	3.7	37.6	46.6
TS7 + 2 x PhOH + 2 x Acetone + Ketone S	16.4	52.5	63.3
I8 + 2 x PhOH + 2 x Acetone + Ketone S	18.4	53.7	61.6
I5 + 2 x PhOH + 2 x Acetone + H_2	-1.8	22.1	30.2
TS4 + 2 x PhOH + 2 x Acetone + H_2	23.8	46.1	55.9
I6 + 2 x PhOH + 2 x Acetone + H_2	-7.4	14.8	22.8
TS5 + PhOH + 2 x Acetone + H_2	-0.9	6.8	17.4
I1 + PhOH + 2 x Acetone + H_2 + I10	-34.2	-3.9	5.3

Supplementary Table 9: Gibbs free energy and enthalpy for mechanism F.

	DG kcal mol^{-1}	DH kcal mol^{-1}	DE kcal mol^{-1}
I1 + 2 x IprOH + Model S	0	0	0
TS1 + Model S + IPrOH	19.2	-0.7	-1.1
I2 + Model S + PhOH + IPrOH	13.3	9.6	8.6
TS2 + Model S + PhOH + IPrOH	19.9	17.1	19.4
I3 + Model S + Acetone + PhOH + IPrOH	1.1	17.5	21.5
TS3 + Model S + Acetone + PhOH	8.9	6.3	9.8
I4 + 2 x PhOH + Acetone + Model S	6.5	22.9	27.1
TS6 + 2 x PhOH + Acetone + Model S	11.5	27.9	34.6
I7 + 2 x PhOH + 2 x Acetone + Model S	3.7	37.6	46.6
TS7 + 2 x PhOH + 2 x Acetone + Model S	16.4	52.5	63.3
I8 + 2 x PhOH + 2 x Acetone + Model S	18.4	53.7	61.6
G1 + 2 x PhOH + 2 x Acetone + H_2	25.3	51.5	59.9
TSG1 + 2 x PhOH + 2 x Acetone + H_2	60.1	85.1	96.0
G2 + 2 x PhOH + 2 x Acetone + H_2	-9.2	14.7	23.5

Supplementary Table 10: Gibbs free energy and enthalpy for mechanism G.

	DG kcal mol^{-1}	DH kcal mol^{-1}	DE kcal mol^{-1}
I1 + IprOH + Model S	0	0	0
TS1 + Model S	19.2	-0.7	-1.1
I2 + Model S + PhOH	13.3	9.6	8.6
TS2 + Model S + PhOH	19.9	17.1	19.4
I3 + Model S + Acetone + PhOH	1.1	17.5	21.5
TS8 + Acetone + PhOH	6.4	-1.2	3.2

I9 + 2 x PhOH + Acetone	6.8	21.0	26.0
TS9 + 2 x PhOH + Acetone	9.9	25.9	33.5
I7 + 2 x PhOH + Acetone + Ketone S	9.8	44.4	53.4
TS7 + 2 x PhOH + Acetone + Ketone S	22.5	59.3	70.1
I8 + 2 x PhOH + Acetone + Ketone S	24.5	60.5	68.5
I5 + 2 x PhOH + Acetone + H_2	4.3	28.9	37.0
TS4 + 2 x PhOH + Acetone + H_2	29.9	52.9	62.7
I6 + 2 x PhOH + Acetone + H_2	-1.3	21.5	29.7
TS5 + PhOH + Acetone + H_2	5.2	13.6	24.3
I1 + PhOH + Acetone + H_2 + I10	-28.1	2.8	12.1

Supplementary Table 11: Gibbs free energy and enthalpy for mechanism H.

4.5 Intrinsic reaction coordinate (IRC) calculations for computed transition states

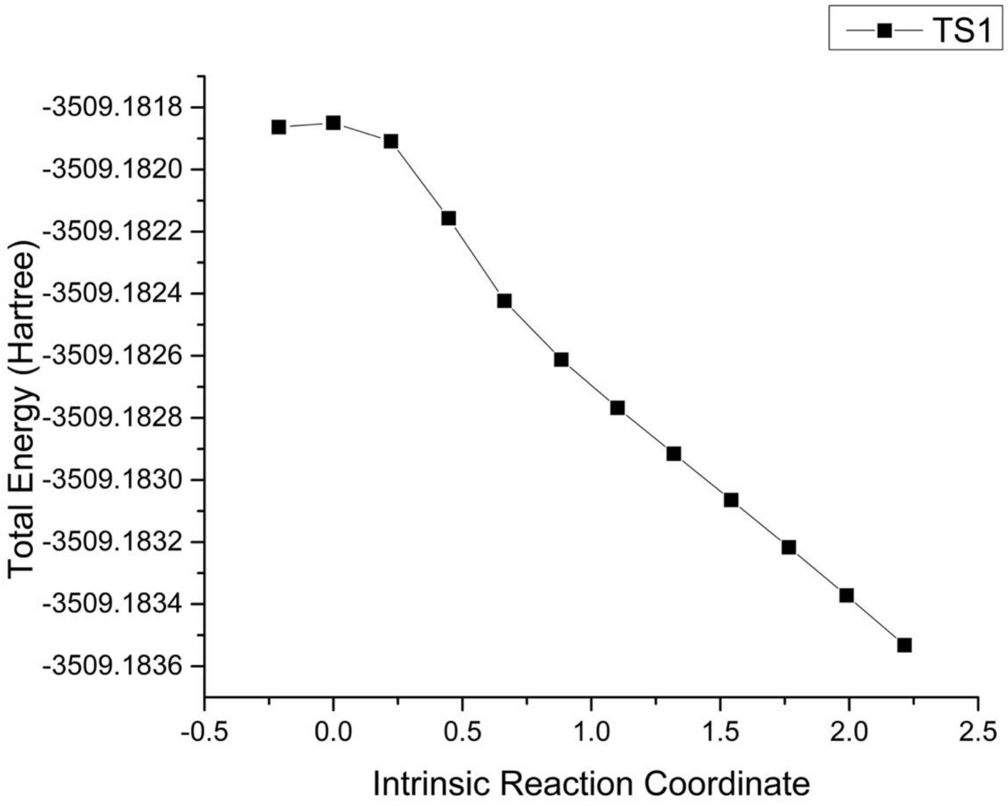

Supplementary Figure 27 | IRC calculation for TS1.

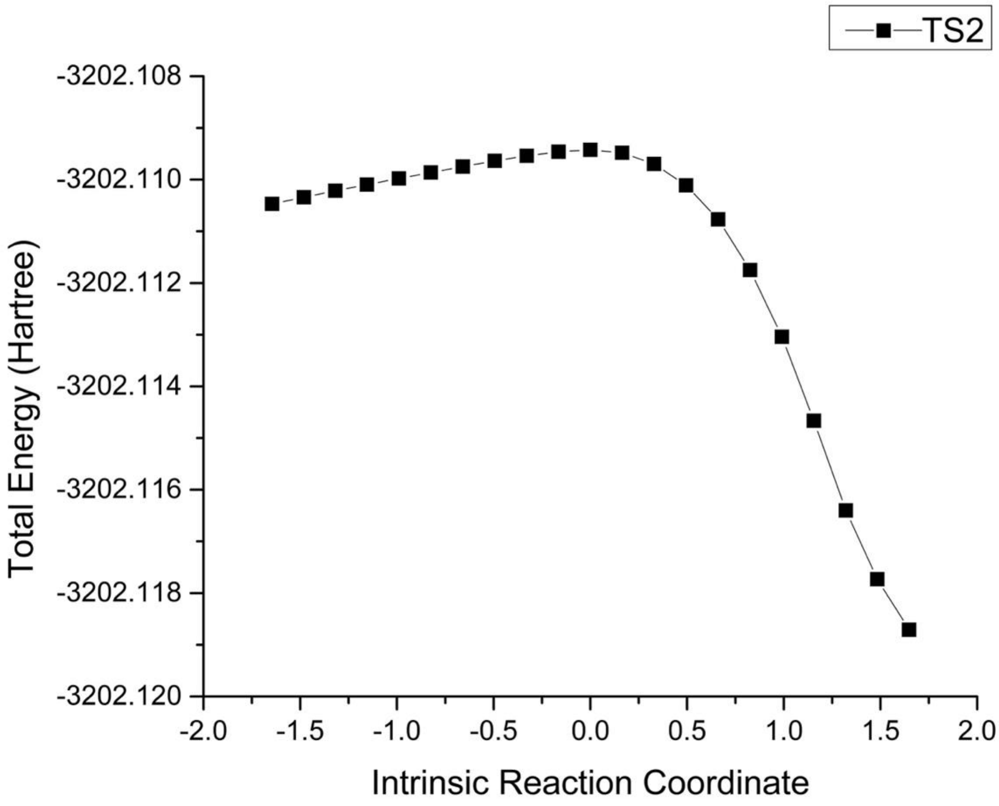

Supplementary Figure 28 | IRC calculation for TS2.

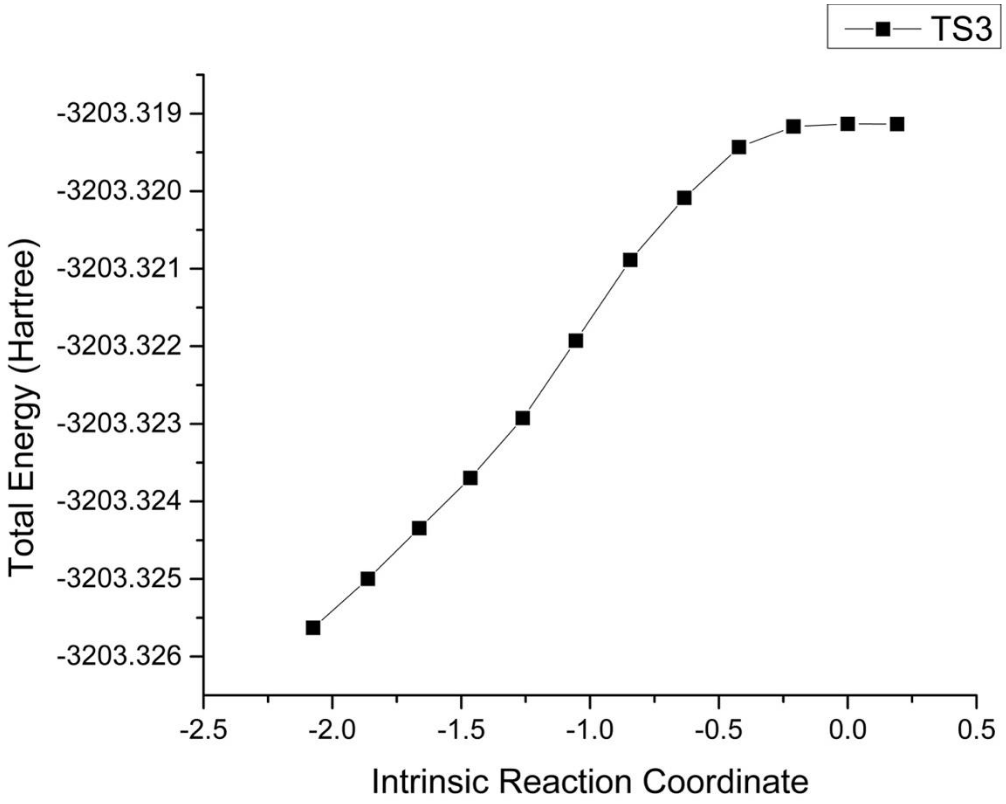

Supplementary Figure 29 | IRC calculation for TS3.

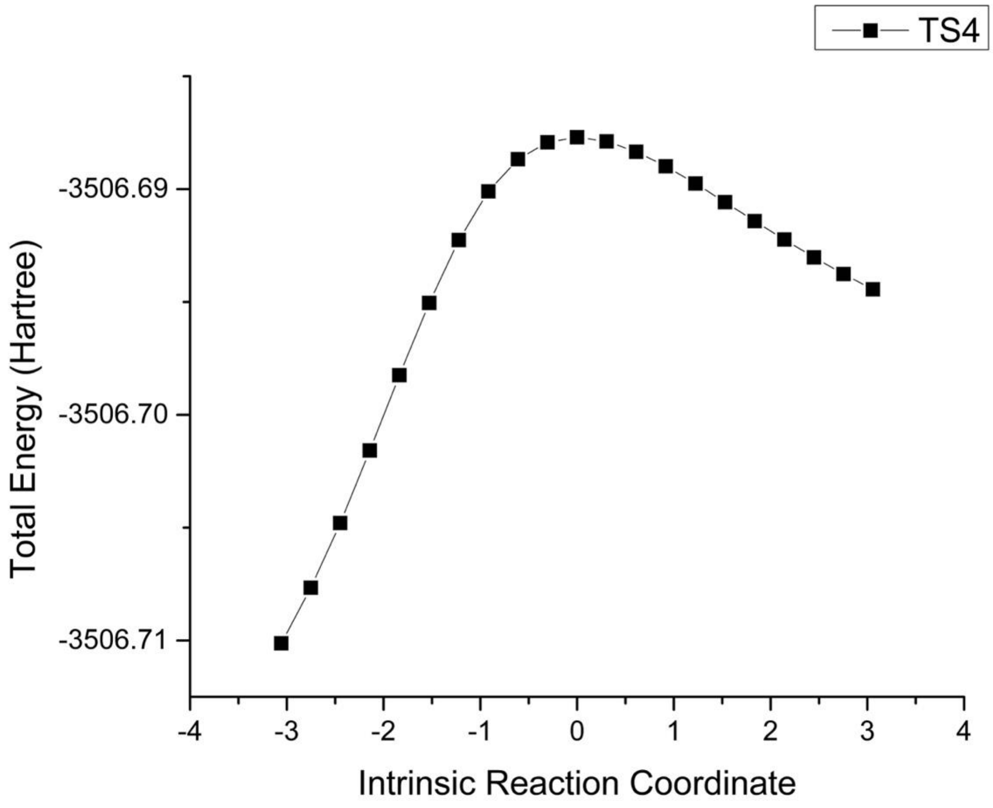

Supplementary Figure 30 | IRC calculation for TS4.

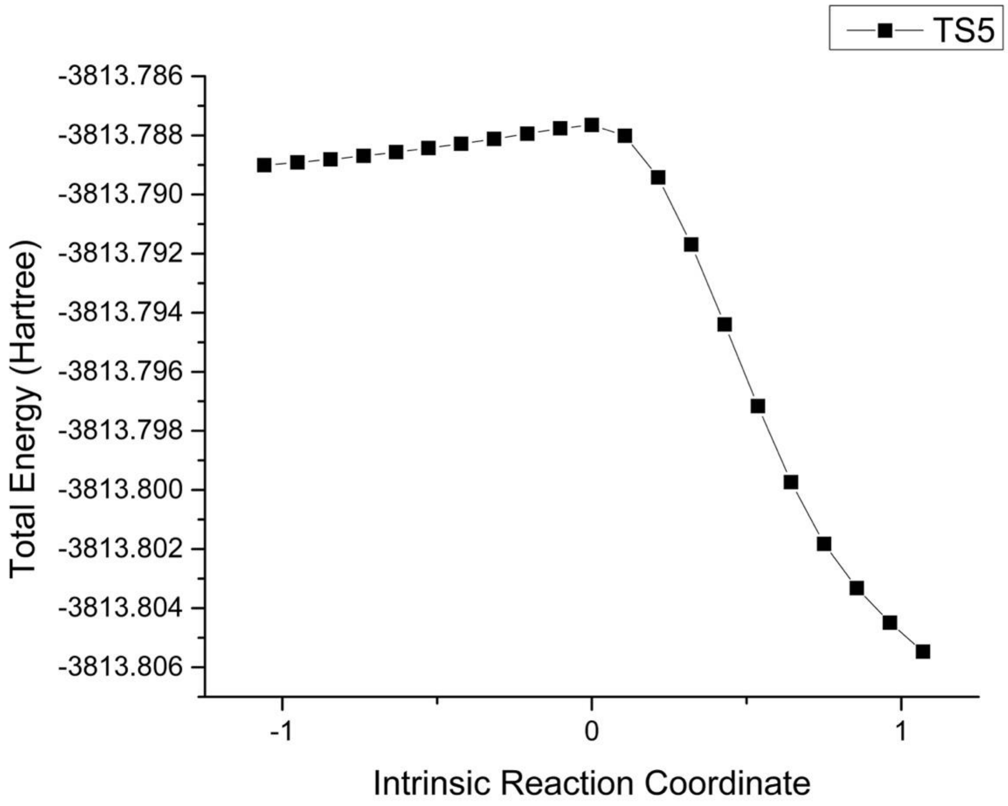

Supplementary Figure 31 | IRC calculation for TS5.

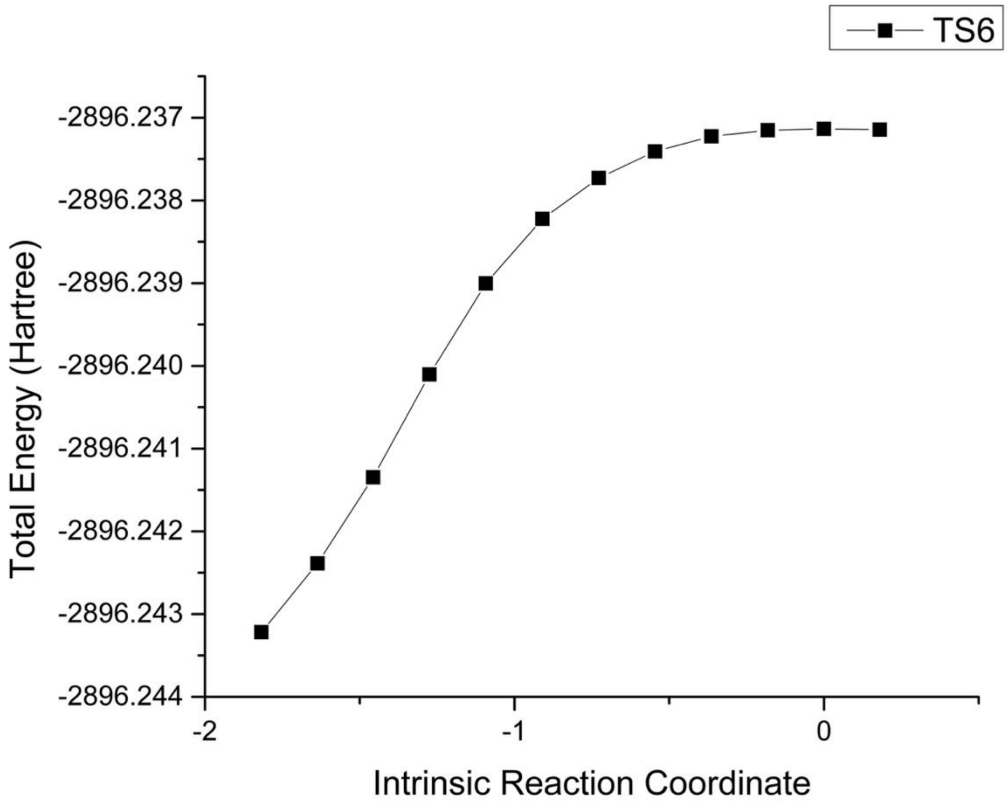

Supplementary Figure 32 | IRC calculation for TS6.

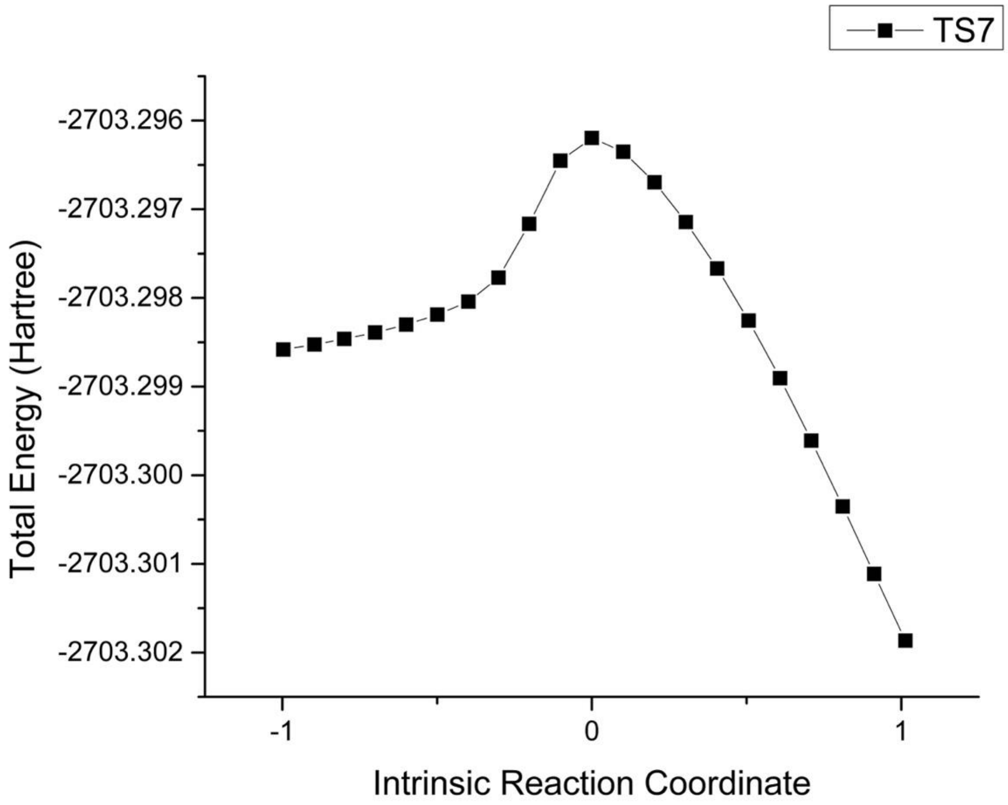

Supplementary Figure 33 | IRC calculation for TS7.

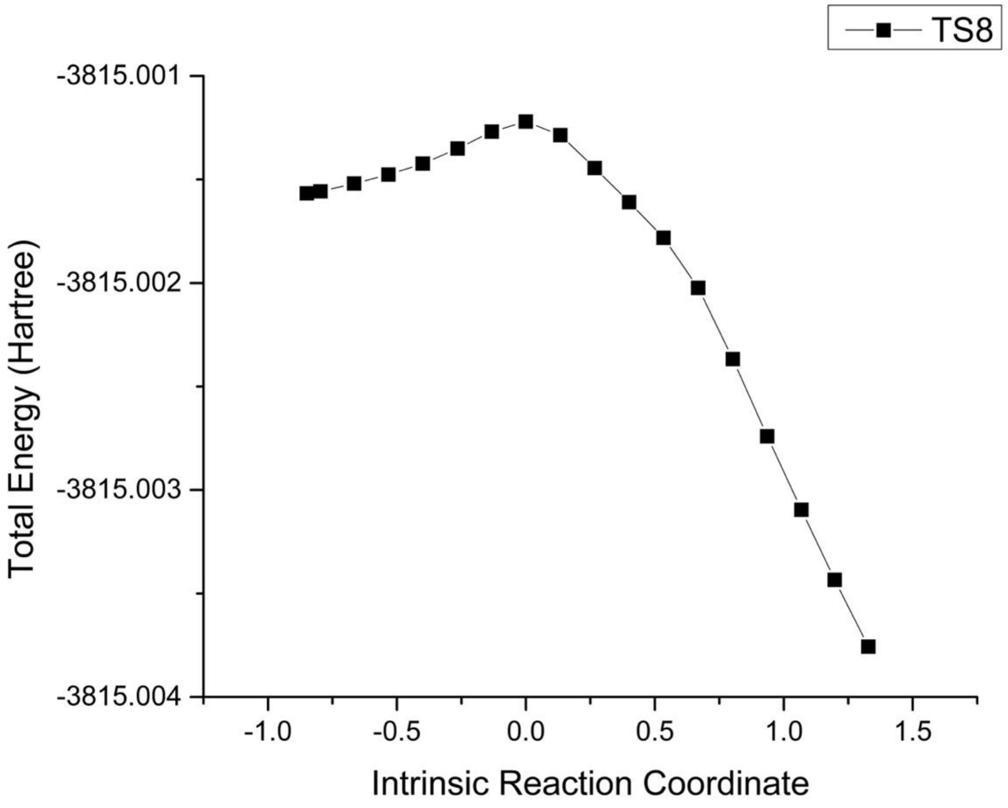

Supplementary Figure 34 | IRC calculation for TS8.

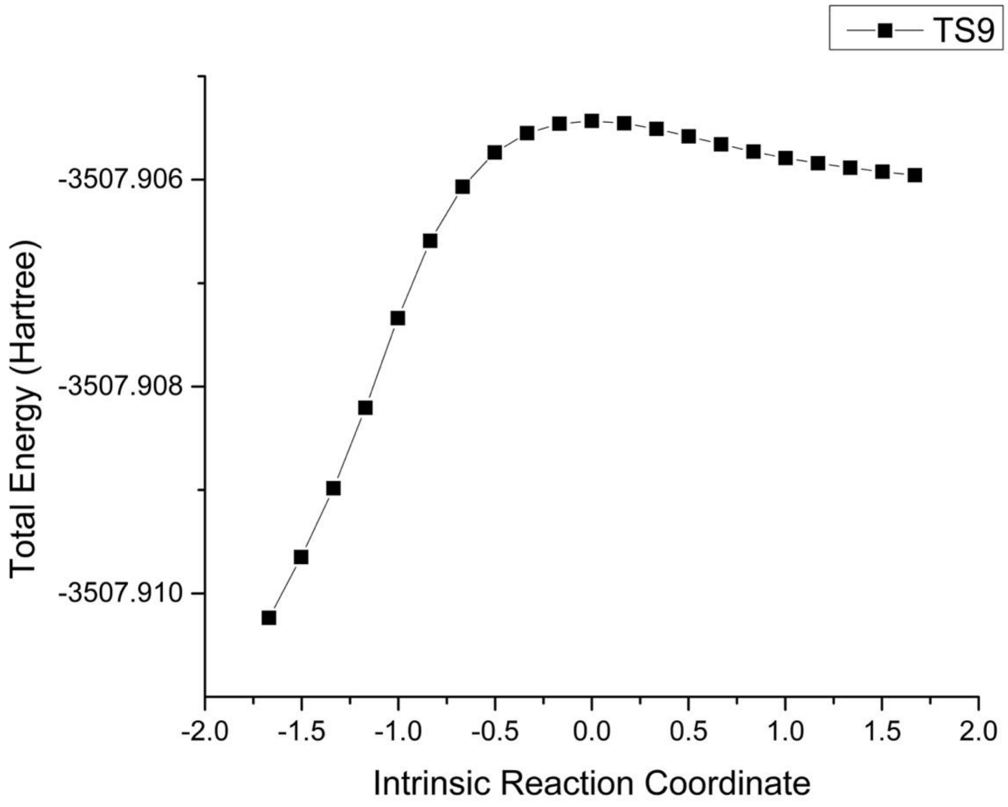

Supplementary Figure 35 | IRC calculation for TS9.

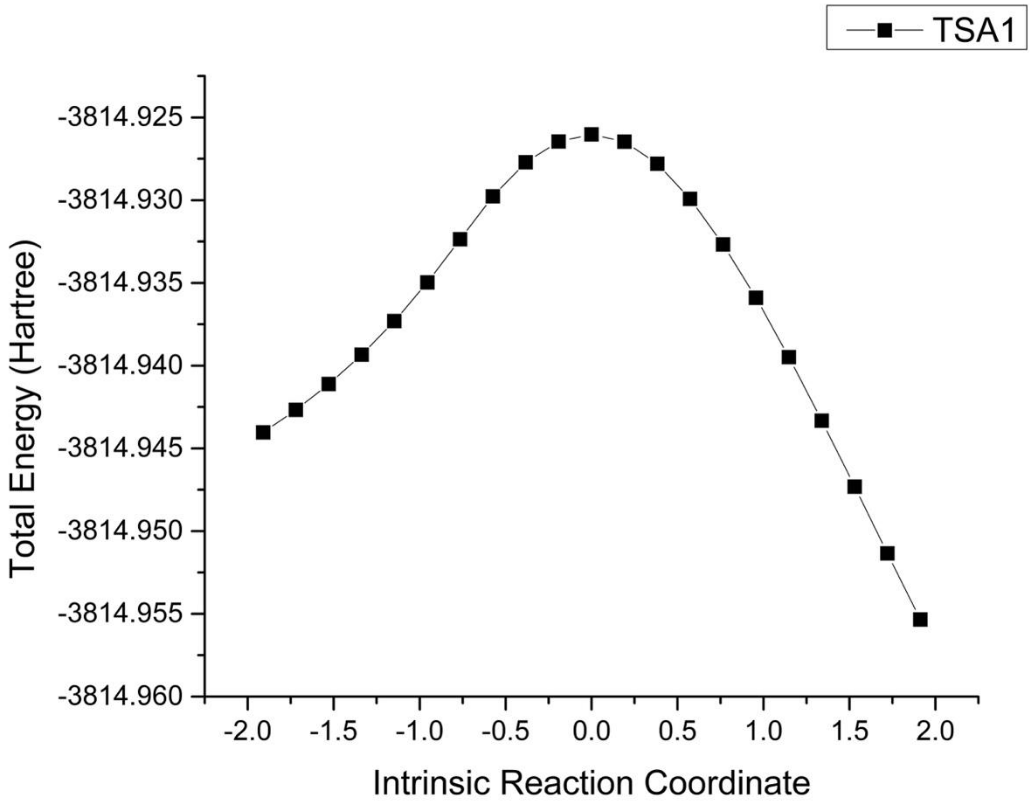

Supplementary Figure 36 | IRC calculation for TSA1.

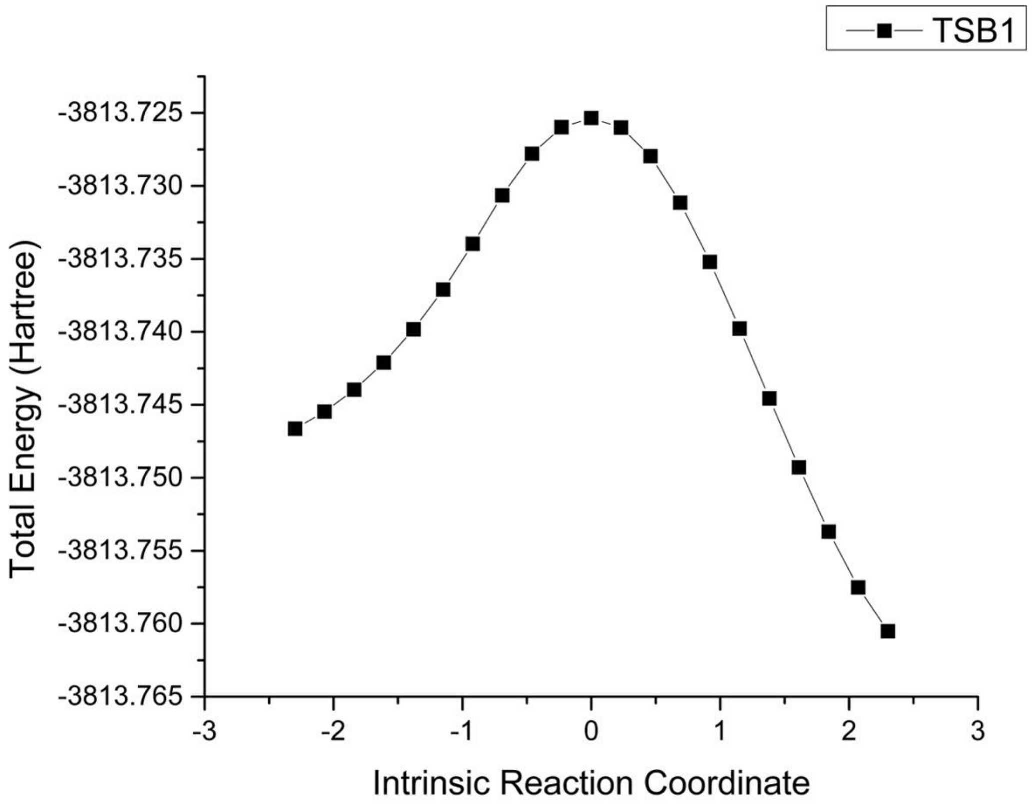

Supplementary Figure 37 | IRC calculation for TSB1.

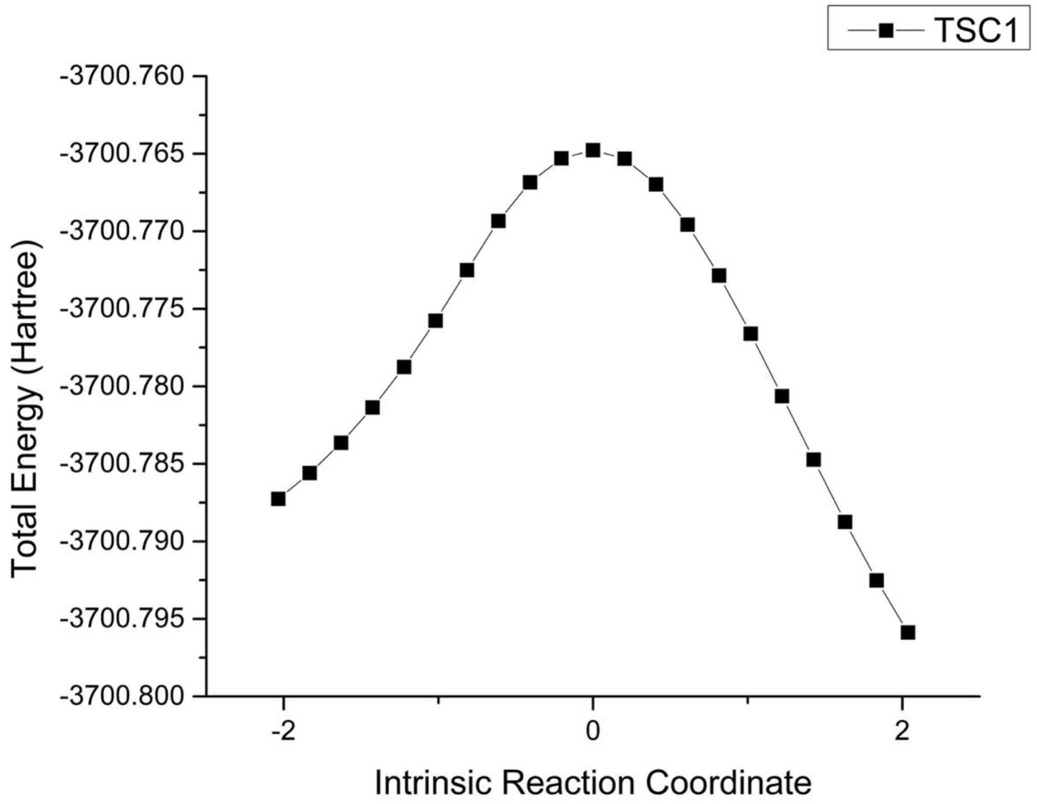

Supplementary Figure 38 | IRC calculation for TSC1.

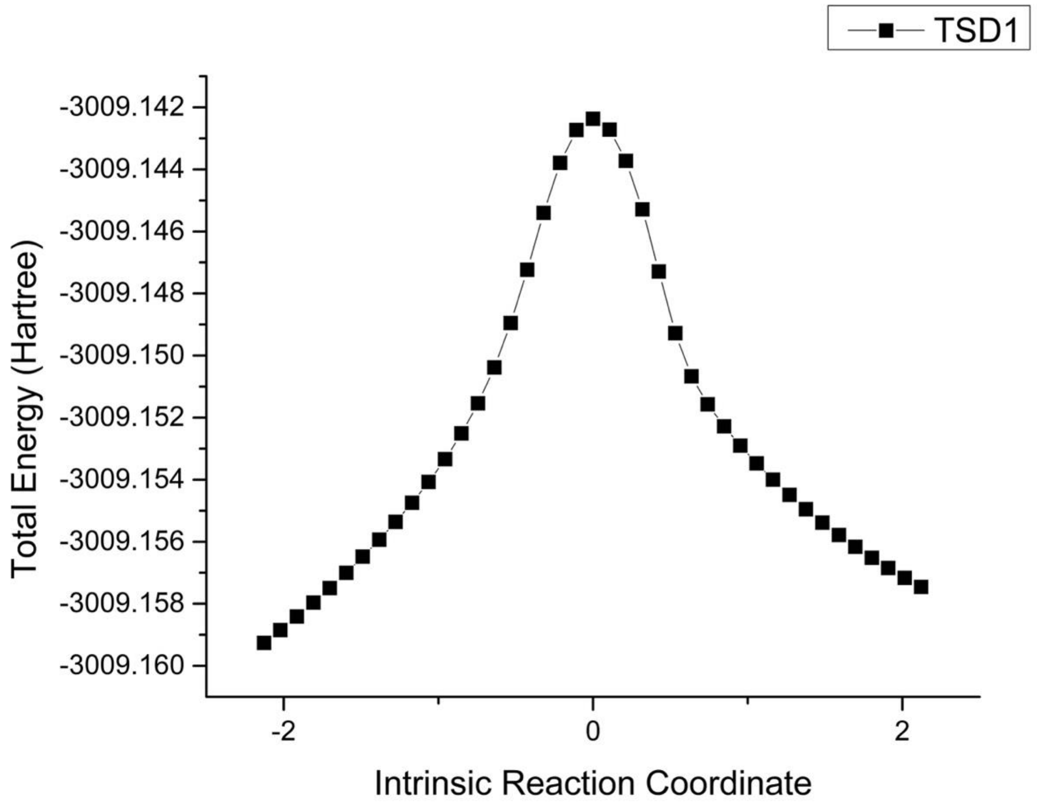

Supplementary Figure 39 | IRC calculation for TSD1.

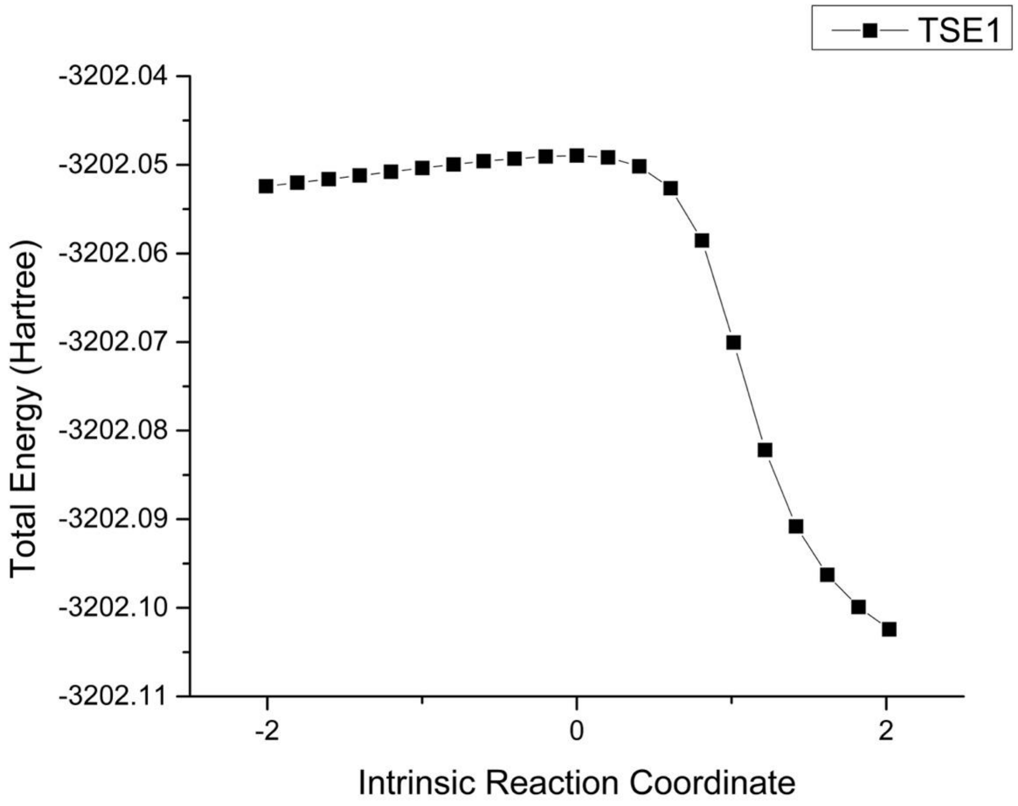

Supplementary Figure 40 | IRC calculation for TSE1.

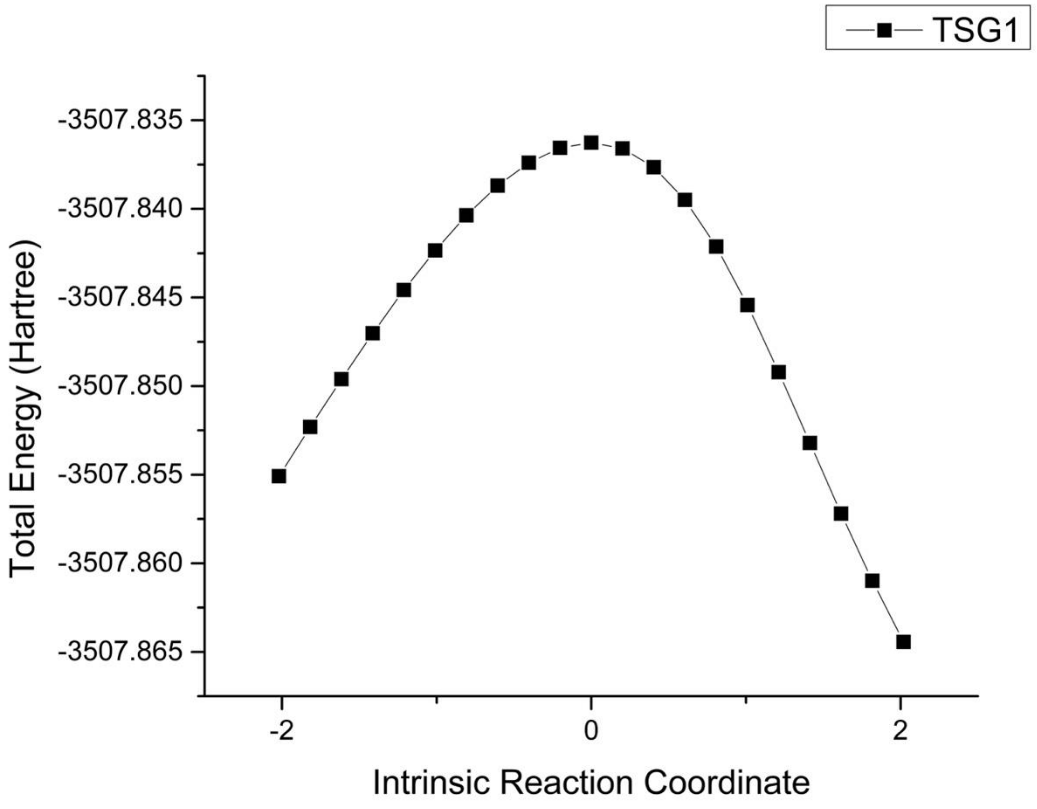

Supplementary Figure 41 | IRC calculation for TSG1.

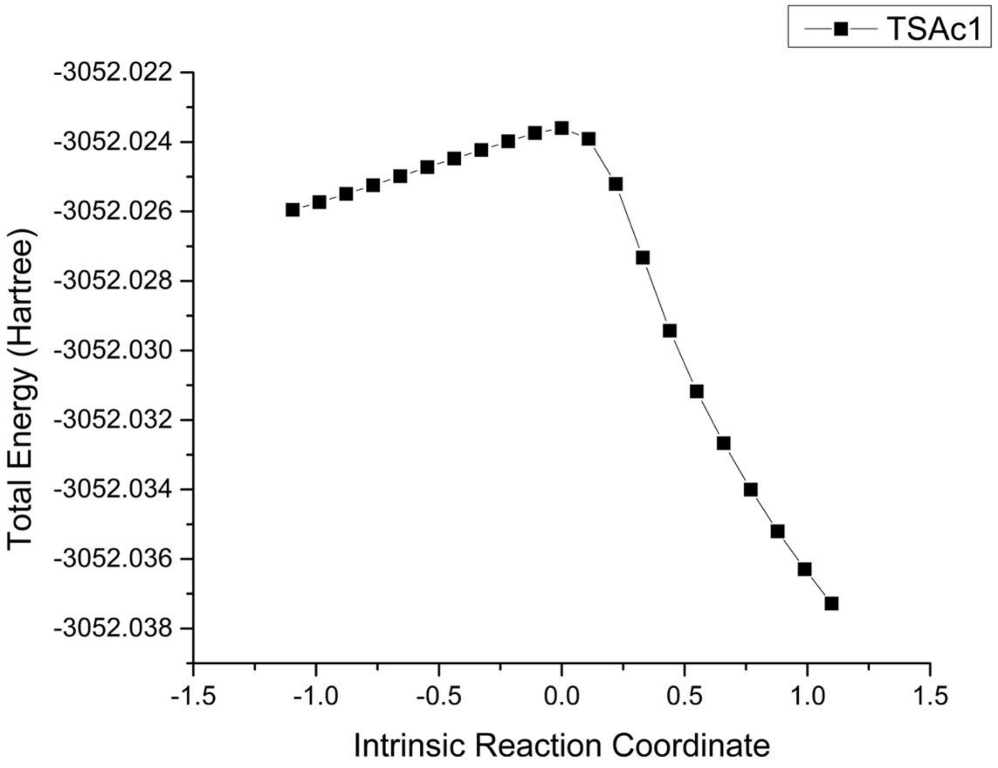

Supplementary Figure 42 | IRC calculation for TSAc1.

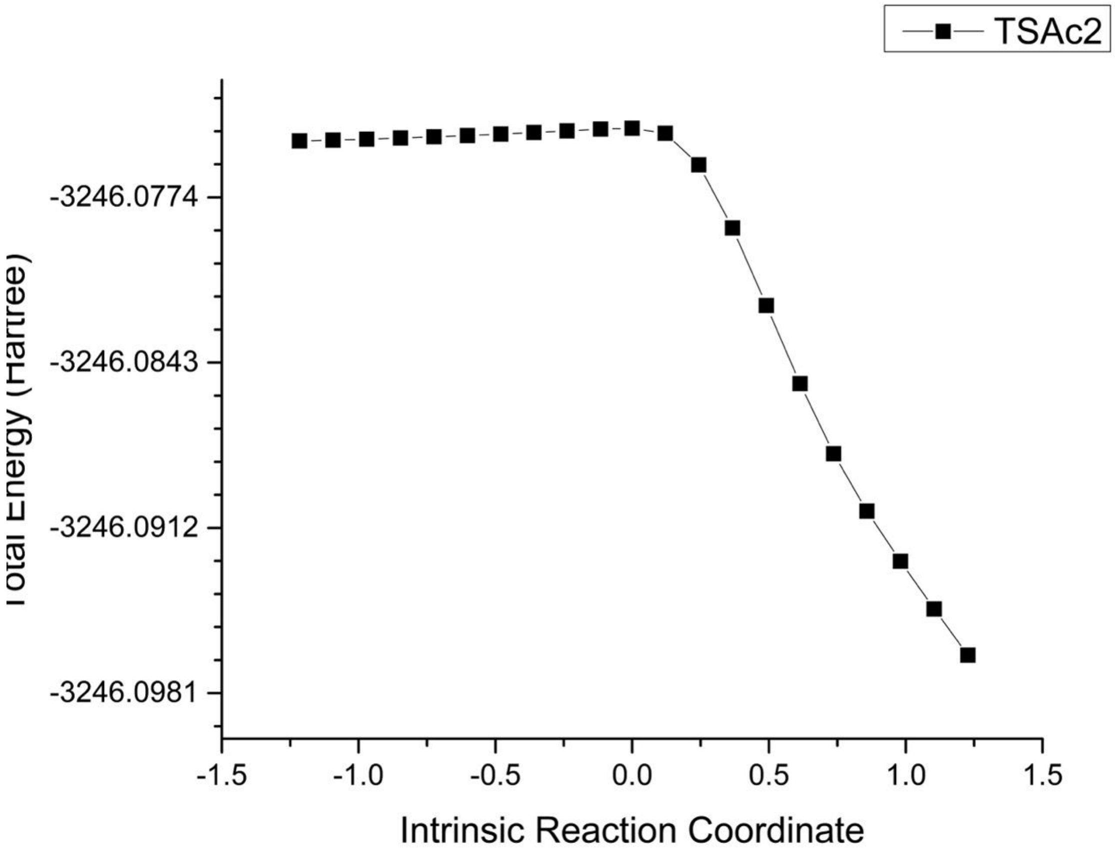

Supplementary Figure 43 | IRC calculation for TSAc2.

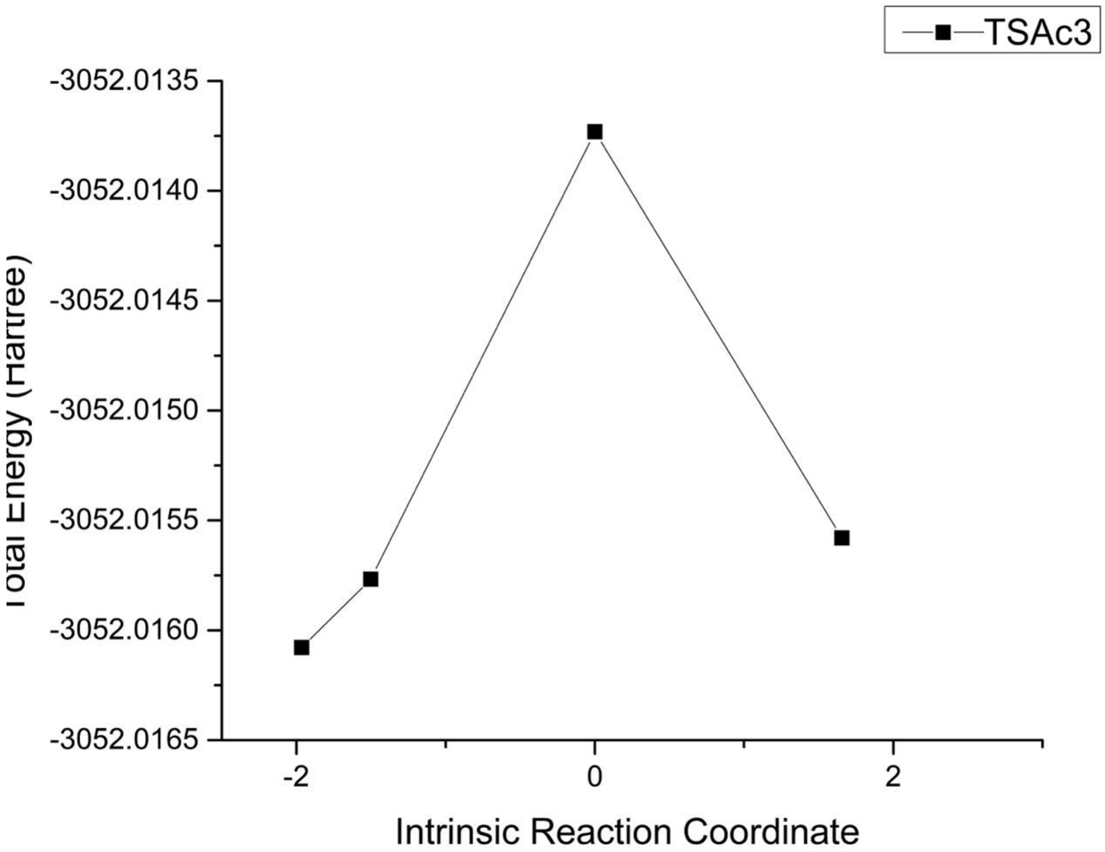

Supplementary Figure 44 | IRC calculation for TSAc3.

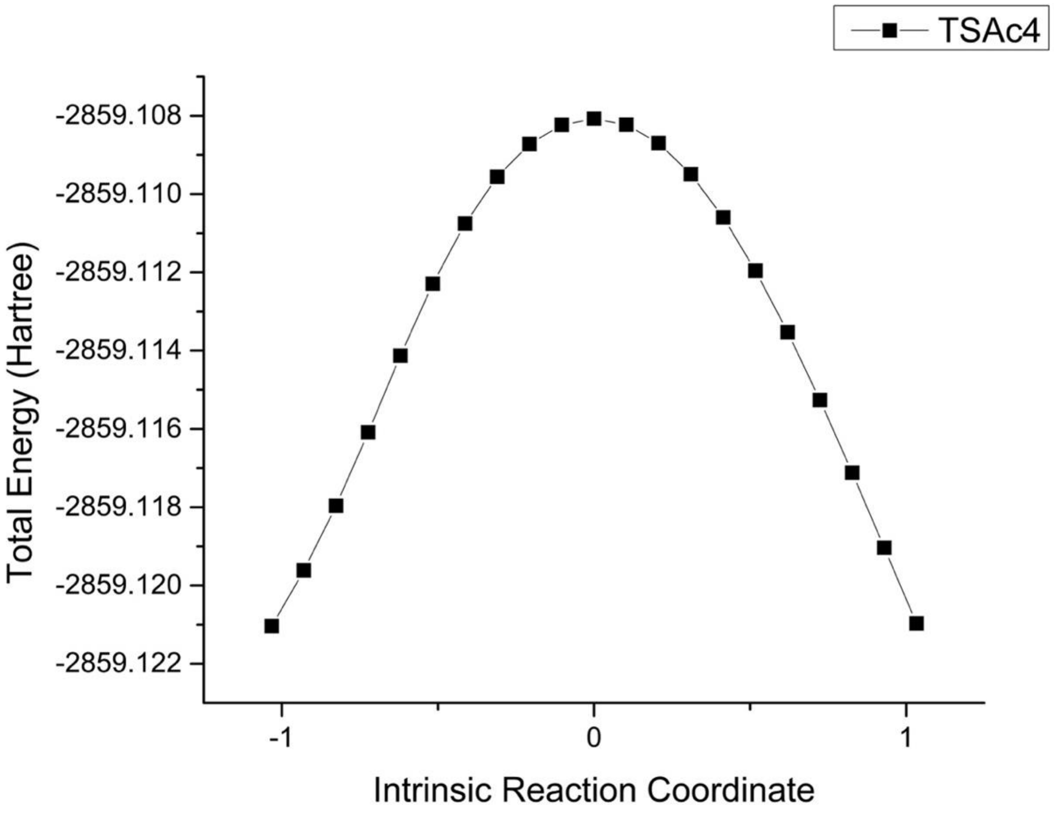

Supplementary Figure 45 | IRC calculation for TSAc4.

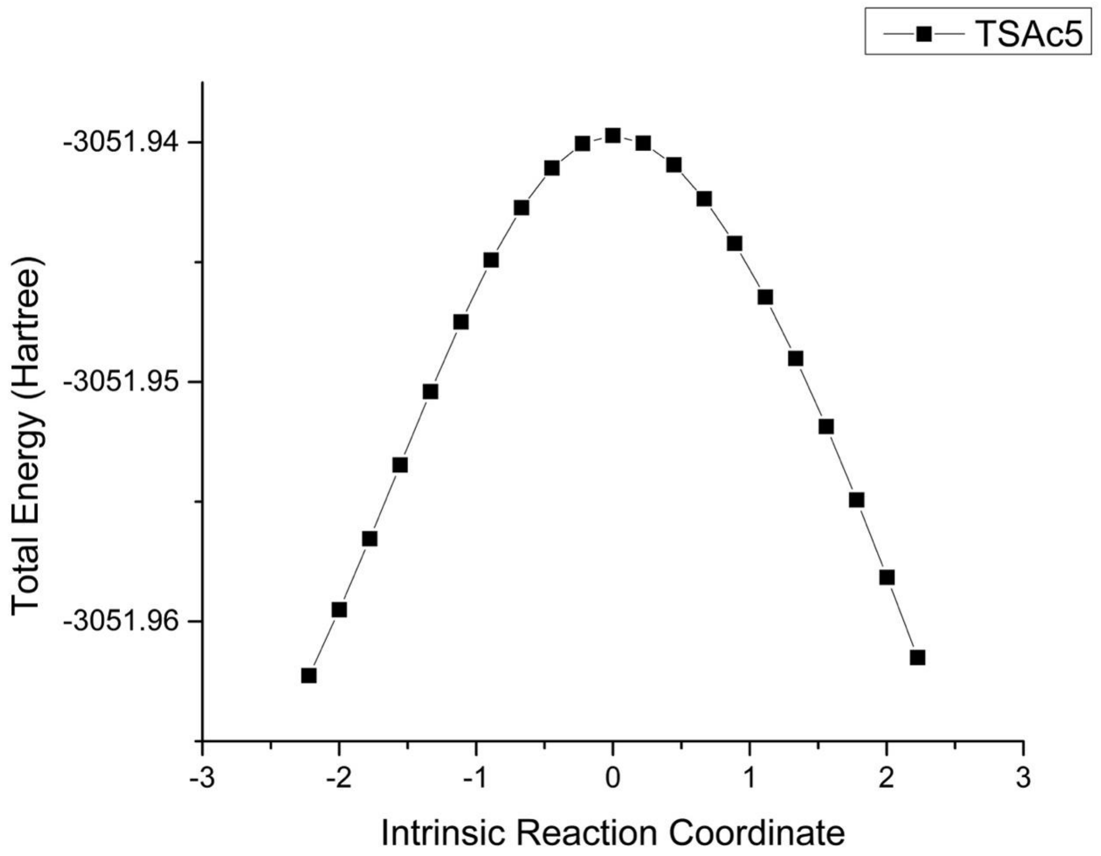

Supplementary Figure 46 | IRC calculation for TSAc5.

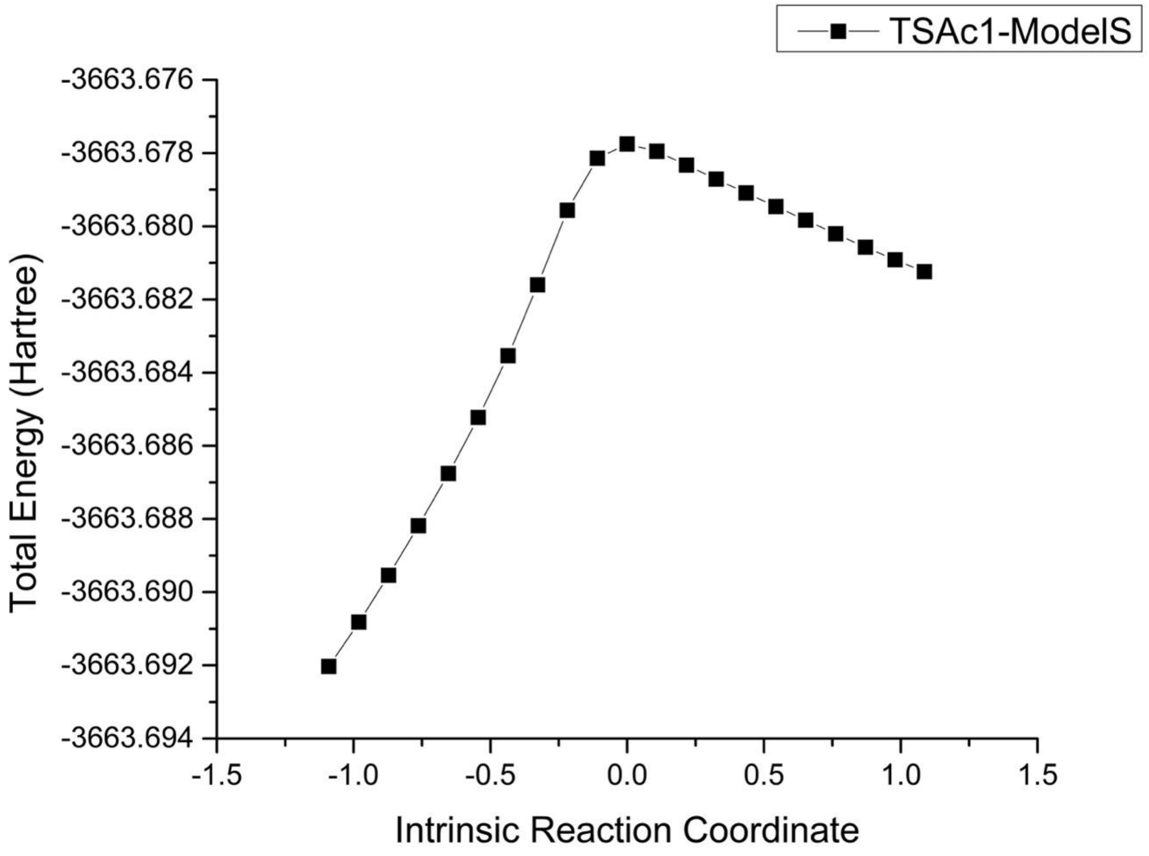

Supplementary Figure 47 | IRC calculation for TSAc1-ModelS.

5. X-Ray crystallographic section

The intensities were empirically corrected for absorption using SCALE3 ABSPACK implemented in CrysAlisPRO (CrysAlisPRO, Oxford Diffraction /Agilent Technologies UK Ltd, Yarnton, England). The unit cell parameters were determined, and the Bragg intensities were integrated using CrysAlisPRO. The structure was solved and refined with SHELXT in Olex2.[17-19]

CCDC 2289877 (Ru-1)

Error PLAT230_ALERT_2_B for F4−C76 caused by failed Hirshfeld Rigid-Bond Test, however no way of correcting this could be found. Error PLAT971_ALERT_2_B for C80 caused larger than expected residual density maximum. No way of amending this could be found. Error PLAT910_ALERT_3_B probably caused by experimental setup. As the crystals had decomposed no new data set with more adequate setup was made.

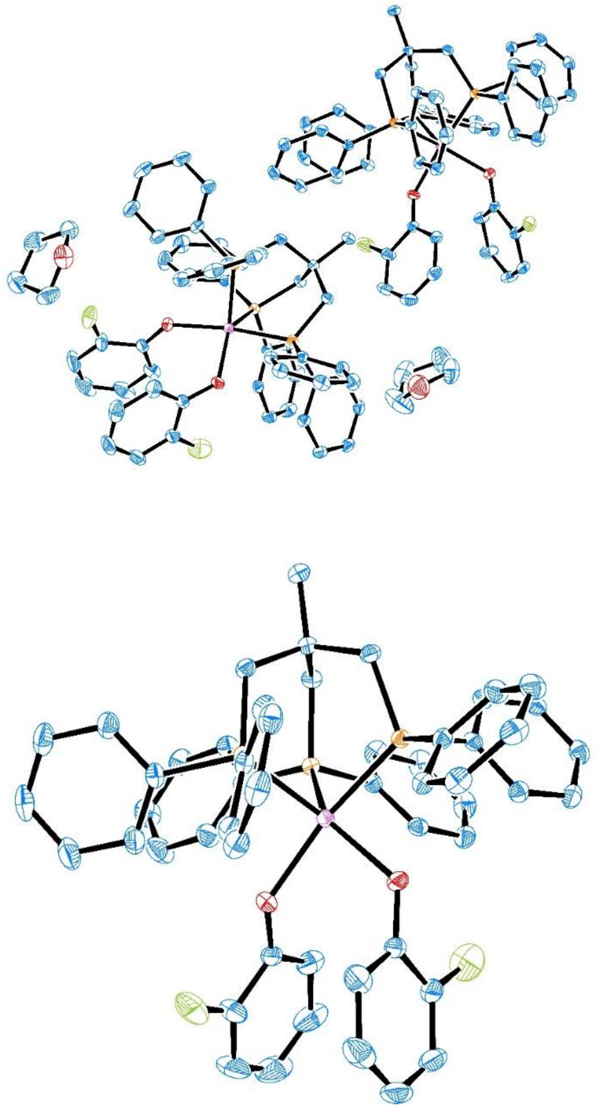

Supplementary Figure 48 | Ortep representation of **Ru-1** with ellipsoids at 50% probability. Blue = Carbon; Pink = Ruthenium; Orange = Phosphine; Red = Oxygen; Lime green = Fluorine. Hydrogens have been omitted for clarity.

Hirshfeld Rigid-Bond Test error for C76−F4, however, attempts at rectifying this were unsuccesful. Test for large positive calculated residual density for C80, however, attempts at rectifying this were unsuccesful. Test for number of missing reflections below Theta(Min) might arise from large unit cell.

Item	value
Molecular formula	$C_{114}H_{110}F_4O_6P_6Ru_2$
Formula weight	2039.97
Crystal system	monoclinic
Space Group	$P2_1/n$
a (Å)	18.2484(3)
b (Å)	17.0077(3)
c (Å)	33.8555(5)
α (°)	90
β (°)	103.959(2)
γ (°)	90
Volume (Å^3)	10197.2(3)
Z	4
T (K)	100
ρ (g cm^{-1})	1.329
λ (Å)	0.71073
μ (mm^{-1})	0.452
# measured refl	164000
# unique refl	25857
R_{int}	0.0559
# parameters	1199
R(F^2), all refl	0.0536
Rw(F^2), all refl	0.1064
Goodness of fit	1.057

CCDC 2289791 (Ru-propionate)

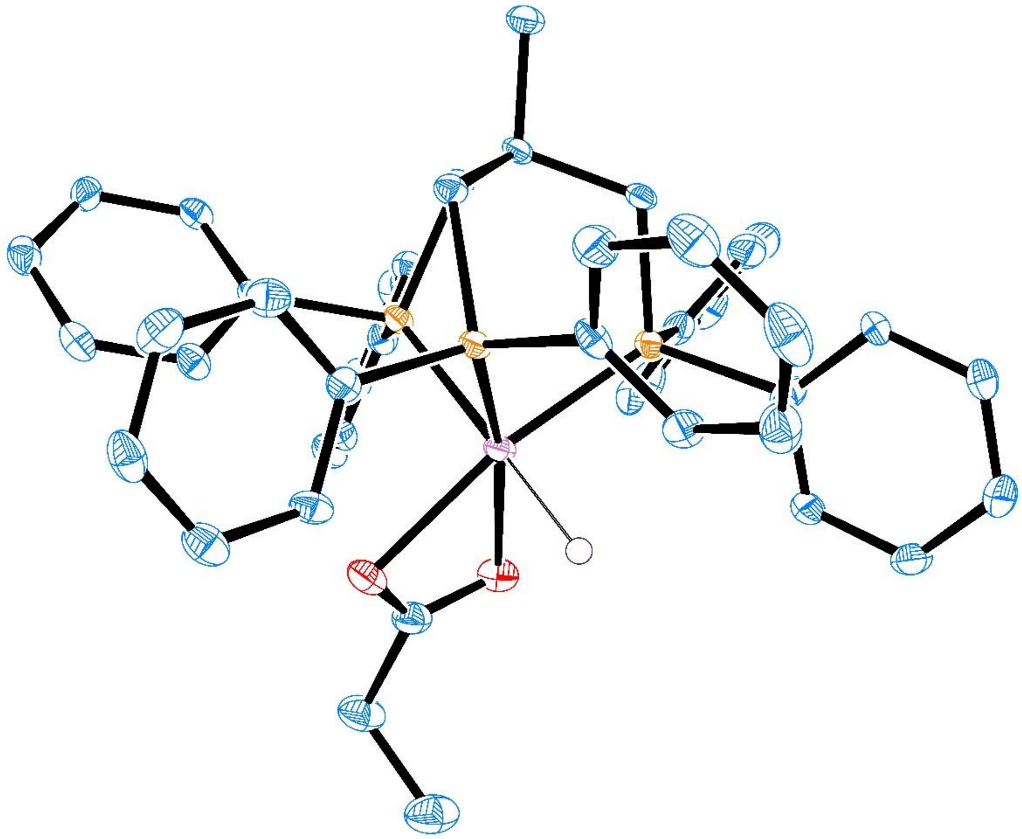

Supplementary Figure 49 | Ortep representation of Ru-propionate with ellipsoids at 50% probability. Blue = Carbon; Pink = Ruthenium; Orange = Phosphine; Red = Oxygen; White = Hydrogen. Non-transition metal hydrogens have been omitted for clarity.

Ru-propionate

Item	value
Molecular formula	$C_{44}H_{45}O_2P$ Ru
Formula weight	799.78
Crystal system	monoclinic
Space Group	Cc
a (Å)	15.0760(4)
b (Å)	13.7616(4)
c (Å)	18.0744(6)
α (°)	90
β (°)	97.833(3)
γ (°)	90
Volume (Å^3)	3714.91(19)
Z	4
T (K)	100
ρ (g cm^{-1})	1.430

λ (Å)	0.71073
μ (mm^{-1})	0.589
# measured refl	38948
# unique refl	8771
R_{int}	0.0649
# parameters	457
R(F^2), all refl	0.0439
Rw(F^2), all refl	0.0951
Goodness of fit	1.028

CCDC 2303540 (triphos-Ru-methylallyl-CO)

Error PLAT971_ALERT_2_B arising from larger than expected residual density for C00N. Effect might be an effect caused by near-proximity to ruthenium metal centre. No way of amending this could be found.

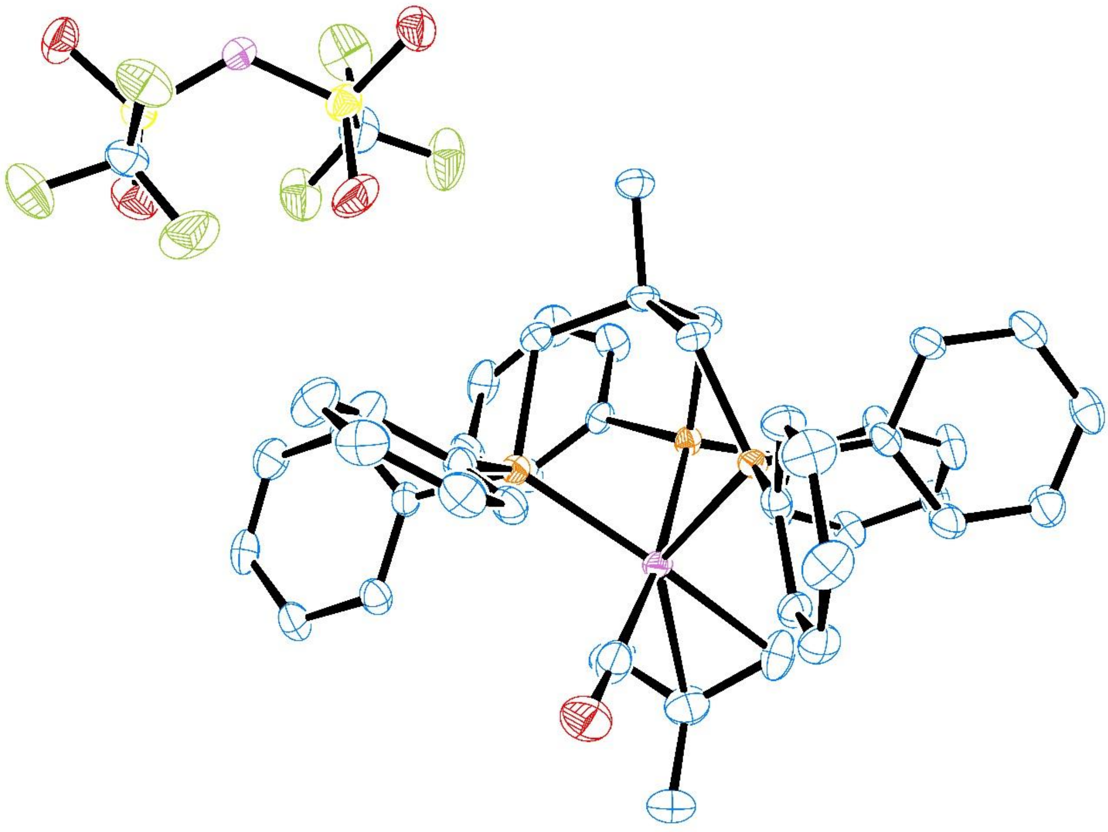

Supplementary Figure 50 | *Ortep representation of Ru-CO with ellipsoids at 50% probability. Blue = Carbon; Pink = Ruthenium; Orange = Phosphine; Red = Oxygen; Yellow = Sulphur; Lemon green = Fluorine. Hydrogens have been omitted for clarity.*

Ru-CO

Item	value
Molecular formula	C_{46} $H_{46}OP_3Ru$, $C_2F_6NO_4S_2$
Formula weight	1088.96
Crystal system	monoclinic
Space Group	P21/n
a (Å)	10.01650(10)
b (Å)	24.2639(3)
c (Å)	19.1591(3)
α (°)	90
β (°)	94.3700(10)
γ (°)	90
Volume (Å^3)	4642.88(10)
Z	4
T (K)	100
ρ (g cm^{-1})	1.558
λ (Å)	0.71073
μ (mm^{-1})	0.604
# measured refl	127102
# unique refl	12557
R_{int}	0.0498
# parameters	597
R(F^2), all refl	0.0697
Rw(F^2), all refl	0.1445
Goodness of fit	1.058

6. References

1 Ahrens, A. et al. Catalytic disconnection of C–O bonds in epoxy resins and composites. *Nature* **617**, 730-737 (2023).

2 Meuresch, M., Westhues, S., Leitner, W. & Klankermayer, J. Tailor-Made Ruthenium-Triphos Catalysts for the Selective Homogeneous Hydrogenation of Lactams. *Angew. Chem. Int. Ed.* **55**, 1392-1395 (2016).

3 Luo, N. et al. Visible-Light-Driven Self-Hydrogen Transfer Hydrogenolysis of Lignin Models and Extracts into Phenolic Products. *ACS Catal.* **7**, 4571-4580 (2017).

4 Schmidbaur, H. & Schier, A. Aurophilic interactions as a subject of current research: an up-date. *Chem. Soc. Rev.* **41**, 370-412 (2012).

5 Friis, S. D., Lindhardt, A. T. & Skrydstrup, T. The Development and Application of Two-Chamber Reactors and Carbon Monoxide Precursors for Safe Carbonylation Reactions. *Acc. Chem. Res.* **49**, 594-605 (2016).

6 Jordan, A. Y. & Meyer, T. Y. C–H activation of pendant alkoxides by tungsten imide complexes. *J. Organomet. Chem.* **591**, 104-113 (1999).

7 Moonen, M. J. H., Westphal, A. H., Rietjens, I. M. C. M. & van Berkel, W. J. H. Enzymatic Baeyer–Villiger Oxidation of Benzaldehydes. *Adv. Synth. Catal.* **347**, 1027-1034 (2005).

8 Rhodes, L. F., Sorato, C., Venanzi, L. M. & Bachechi, F. Ruthenium(II) solvento complexes containing the tripod-like ligands $MeC(CH_2EPh_2)_3$ (E = P or As) and their reactions with carbon monoxide. Crystal and molecular structure of $[Ru_2(\mu\text{-}Cl)_3(MeC(CH_2PPh_2)_3)_2][BPh_4]$. *Inorg. Chem.* **27**, 604-610 (1988).

9 Sun, H. et al. Practical Gas Cylinder-Free Preparations of Important Transition Metal-Based Precatalysts Requiring Gaseous Reagents. *Org. Process Res. Dev.* **25**, 2300-2307 (2021).

10 Frisch, M. J. et al. Gaussian 16, Revision B.01. Gaussian, Inc.,: Wallingford CT 2016.

11 Zhao, Y. & Truhlar, D. G. The M06 suite of density functionals for main group thermochemistry, thermochemical kinetics, noncovalent interactions, excited states, and transition elements: two new functionals and systematic testing of four M06-class functionals and 12 other functionals. *Theor. Chem. Acc.* **120**, 215-241 (2008).

12 Barone, V. & Cossi, M. Quantum Calculation of Molecular Energies and Energy Gradients in Solution by a Conductor Solvent Model. *J. Phys. Chem. A* **102**, 1995-2001 (1998).

13 McLean, A. D. & Chandler, G. S. Contracted Gaussian basis sets for molecular calculations. I. Second row atoms, Z=11–18. *J. Chem. Phys.* **72**, 5639-5648 (2008).

14 Krishnan, R., Binkley, J. S., Seeger, R. & Pople, J. A. Self-consistent molecular orbital methods. XX. A basis set for correlated wave functions. *J. Chem. Phys.* **72**, 650-654 (2008).

15 Blaudeau, J.-P., McGrath, M. P., Curtiss, L. A. & Radom, L. Extension of Gaussian-2 (G2) theory to molecules containing third-row atoms K and Ca. *J. Chem. Phys.* **107**, 5016-5021 (1997).

16 Pracht, P., Bohle, F. & Grimme, S. Automated exploration of the low-energy chemical space with fast quantum chemical methods. *Phys. Chem. Chem. Phys.* **22**, 7169-7192 (2020).

17 Dolomanov, O. V., Bourhis, L. J., Gildea, R. J., Howard, J. A. K. & Puschmann, H. OLEX2: a complete structure solution, refinement and analysis program. *J. Appl. Crystallogr.* **42**, 339-341 (2009).

18 Sheldrick, G. A short history of SHELX. *Acta Crystallogr. A* **64**, 112-122 (2008).

19 Sheldrick, G. Crystal structure refinement with SHELXL. *Acta Crystallogr. C* **71**, 3-8 (2015).
